# The Applied Sport Science and Medicine of Powerlifting and Para Powerlifting: A Systematic Scoping Review with Recommendations for Future Research

**DOI:** 10.1007/s40279-025-02305-3

**Published:** 2025-09-09

**Authors:** Kade Silverthorne, Matthew Morrison, Nicholas Cowley, Gabriella Munteanu, Mark W. Creaby, Ryan G. Timmins, Chieh-Ying Chiang, Jonathon Weakley

**Affiliations:** 1 School of Behavioural and Health Sciences, Australian Catholic University, McAuley at Banyo, Brisbane, Australia; 2 Sports Performance, Recovery, Injury and New Technologies (SPRINT) Research Centre, Australian Catholic University, Brisbane, QLD Australia; 3 Centre for Human Factors and Sociotechnical Systems, University of the Sunshine Coast, Sippy Downs, QLD Australia; 4 Department of Sports Training Science - Combats, National Taiwan Sport University, Taoyuan, Taiwan; 5 Taiwan Institute of Sports Science, Kaohsiung City, Taiwan; 6 Carnegie Applied Rugby Research (CARR) Centre, Carnegie School of Sport, Leeds Beckett University, Leeds, UK

## Abstract

**Background:**

Powerlifting is a strength sport featuring some of the world’s strongest athletes. Recent decades have seen an exponential increase in research into the applied sport science and medicine of powerlifting and its Paralympic counterpart, para powerlifting. A scoping review of the area would provide athletes, coaches, policymakers, and researchers with an overview of the existing evidence to support performance, reduce injury, and foster further growth of these sports.

**Objectives:**

The primary objectives were to identify the current research into the applied sport science and medicine of powerlifting and para powerlifting, analyse the characteristics of the research, provide a brief summary of the research in each area of sport science and medicine, identify gaps in the current literature, and provide recommendations for future research.

**Methods:**

Systematic searches of SPORTDiscus, CINAHL, MEDLINE, and Scopus were performed from the earliest record to June 2025 (Open Science Framework registration: https://osf.io/fkjsz), and the reference lists of several pre-existing systematic reviews were manually searched. Studies were eligible for inclusion if they investigated powerlifting or para powerlifting as a sport or the applied sport science of powerlifters or para powerlifters from a performance or injury perspective.

**Results:**

A total of 2117 articles were identified in the database search, with three additional eligible studies discovered through other sources. In total, 218 studies met the inclusion criteria and were ultimately included in the review. The most researched sport science and medicine topic was physical qualities (*n* = 48), followed by competition (*n* = 45), training (*n* = 38), biomechanics (*n* = 36), nutrition and supplementation (*n* = 25), injury (*n* = 18), and psychology (*n* = 8). More than half of the included studies were published in 2020 or later, and researchers from the USA were the most prolific with 57 publications. Para powerlifting was investigated in 45 studies, which mostly originated from Brazil (*n* = 31). Participants represented varying levels of competition, powerlifting divisions, and age categories, although many studies did not clearly report these characteristics. Only seven studies investigated female athletes exclusively.

**Conclusion:**

This scoping review summarises the current literature investigating powerlifting and para powerlifting and can be used to enhance the applied sport science and medicine within the sports. While the amount of research has grown considerably in recent years, it is evident that certain demographics and areas remain under-investigated (e.g., injury mechanisms) or warrant updated examination (e.g., the prevalence of performance-enhancing drug use, which was last reported in 2003 and is currently unknown). Thus, this review highlights several areas for future research based on the gaps in the existing literature and provides a range of recommendations that can be implemented to improve reporting, transparency, and interpretation.

**Supplementary Information:**

The online version contains supplementary material available at 10.1007/s40279-025-02305-3.

## Key Points

**Table:** 

The amount of research investigating powerlifting or para powerlifting has more than doubled since 2020. Studies have spanned a range of demographics and levels of competition, although only 3% of studies solely investigated females.
The most researched area of sport science and medicine is physical qualities, while psychology is the least researched topic. Despite the breadth of the existing evidence, several gaps have been identified and should be investigated further, including the mechanisms behind common powerlifting injuries and the use of performance-enhancing drugs.
Clearer reporting of participant characteristics is suggested to aid in the interpretation of future powerlifting research. Additional recommendations include the use of standard powerlifting terminology and greater emphasis on scientific rigour.

## Introduction

Powerlifting is a strength sport featuring some of the world’s strongest athletes. The sport requires competitors to complete the barbell back squat, bench press, and deadlift to achieve the heaviest summated ‘total’. Similarly, para powerlifting is an adapted version of powerlifting for athletes with disabilities in which only the bench press is performed. The USA held the first national powerlifting championships in 1964; the International Powerlifting Federation (IPF), which was established in 1972, is now considered the premier governing body for drug-tested powerlifting, with affiliated national federations in over 100 countries [1, 2]. Additionally, since the earliest research in the mid-1970s [3–5], the scientific literature underpinning powerlifting and para powerlifting performance has grown substantially across a range of areas.

In powerlifting competitions, athletes perform a one-repetition maximum (1RM) in each of the three lifts. Competitions are separated into divisions based on the permitted supportive equipment: ‘raw’, where only neoprene sleeves can be worn on the knees during the squat; ‘wraps’, where elastic knee wraps are used during the squat; and ‘equipped’, where assistive lifting suits are worn [6, 7]. Additionally, competitions may be ‘tested’ for performance-enhancing drugs (PEDs) or ‘untested’ [8]. In competition, lifters are separated into ‘flights’ of up to 10 athletes, usually according to weight class or age category. Starting with the squat, each competitor within the first flight performs their self-selected opening attempt (or ‘opener’) one-by-one in ascending order of weight on the barbell, and a second and third attempt is performed the same way with self-selected increments in weight. Subsequent flights perform their three squat attempts according to the same protocol; this entire process is then repeated for the bench press and the deadlift. Three referees judge each attempt as a good lift (‘white light’) or no lift (‘red light’) based on adherence to that lift’s specific rules [7]. While these rules differ slightly between powerlifting federations, see van den Hoek et al. [9] for a summary of each lift’s typical requirements. Finally, each competitor’s heaviest successful squat, bench press, and deadlift are summated to obtain their total, upon which the winners are determined within each weight class. An overall male and female winner may also be decided by using a standardised coefficient to normalise results relative to body mass. The once-prevalent Wilks formula [10] is one such example, while contemporary systems such as IPF GL Points [11] and DOTS score [12] now predominate.

Para powerlifting is a distinct sport for athletes with disabilities. Its governing body is World Para Powerlifting, and it has been included in the Paralympic Games since 1984 [13]. Eligible athletes may have spinal cord injuries, short stature, neuromuscular disorders, or other impairments [14]. Para powerlifters compete to achieve the heaviest bench press across three attempts, with minor technical differences compared with powerlifting such as the allowance of straps to secure athletes to the bench where required [15].

There has been substantial growth in the applied sport science and medicine of strength sports (e.g., powerlifting and para powerlifting [16–18], Olympic weightlifting [17, 19, 20], strongman [21, 22], and CrossFit [23, 24]) in recent years. Powerlifting requires unique skills [25, 26], physical qualities [27, 28], preparation [29, 30], and competition strategies [31, 32] which warrant sport-specific scientific evidence to support performance. Additionally, due to the physical demands placed upon the athletes, powerlifting has a unique occurrence of injuries that commonly affect the shoulder, lower back, and knee [17, 33, 34], with an estimated incidence of 1.0–4.4 injuries per 1000 h of training [17]. Furthermore, para powerlifters have been shown to sustain more injuries than other Paralympic athletes [35, 36], possibly due to the highly repetitive nature of the sport and the heavy loads placed upon the upper limbs [36]. Despite the unique demands and requirements of these sports, a comprehensive review of the applied sport science and medicine of powerlifting and para powerlifting is yet to be performed. While previous reviews have been conducted, they have been limited to specific topics (e.g., raw powerlifting performance [16]). Therefore, a broader overview of the literature across a range of domains is warranted to consolidate the existing evidence and inform future research directions. The aim of this scoping review was thus to provide an overview of the existing research on the applied sport science and medicine of powerlifting and para powerlifting. Our four primary objectives were to (1) conduct a systematic search of the published literature, (2) analyse the characteristics of the research, (3) provide a summary of the research in each area of sport science and medicine, and (4) identify gaps in the current literature and provide recommendations for future research.

## Methods

### Design and Search Strategy

A scoping review was performed in accordance with the Preferred Reporting Items for Systematic reviews and Meta-Analyses extension for Scoping Reviews (PRISMA-ScR) [37] and was registered with the Open Science Framework (registration: https://osf.io/fkjsz). A systematic search of electronic databases (SPORTDiscus, CINAHL, MEDLINE, and Scopus) was executed from the earliest record to 26 June 2025. The search query, shown in Table 1, was limited to search within the fields ‘authors’, ‘subjects’, ‘keywords’, ‘title information’, and ‘abstracts’ (SPORTDiscus, CINAHL, and MEDLINE) or ‘article title’, ‘abstract’, and ‘keywords’ (Scopus). Results were filtered further to include only ‘Academic Journals’ (SPORTDiscus, CINAHL, and Medline) or ‘Articles’ (Scopus). Additionally, three previous systematic reviews of powerlifting or para powerlifting research were identified [16, 18, 38], and their reference lists were manually searched for any other potentially eligible studies. However, no additional articles that met our inclusion criteria were discovered during this process.

**Table 1:** Search terms used in the search query

Term 1	Term 2
Powerlift* OR power-lift* OR parapowerlift* OR para-powerlift* OR parapower-lift* OR para-power-lift*	Demand* OR characteristic* OR competition* OR perform* OR skill* OR techni* OR strateg* OR attempt* OR physical OR test* OR qualit* OR total* OR anthropometr* OR composition OR hypertroph* OR “muscle mass” OR “lean mass” OR neuromuscular OR strength OR “rep* max*” OR velocity OR speed OR power OR fitness OR jump OR physiolog* OR train* OR load OR exposure OR program* OR periodi* OR fatigu* OR rest* OR recover* OR “muscle damage” OR develop* OR intervention* OR injur* OR health OR wellness OR wellbeing OR risk* OR incidence OR safe* OR psycholog* OR mental OR biomechanic* OR kinetic* OR kinematic* OR joint* OR torque* OR nutrition* OR diet OR food* OR supplement*

Search terms 1 and 2 were combined with the Boolean operator ‘AND’

### Study Selection

Search results were exported from each database and imported into a systematic review management tool (Covidence, Veritas Health Innovation, Melbourne, Australia). Duplicates were removed, and titles and abstracts were screened independently by two researchers (KS, NC) against the inclusion criteria. Disagreements were resolved through discussion or via a third researcher (JW). Articles which could not be eliminated by the title or abstract were retrieved and evaluated for eligibility via a full-text review.

Studies were eligible for inclusion if they investigated powerlifting or para powerlifting as a sport or the applied sport science or medicine of powerlifters or para powerlifters from a performance or injury perspective. Biomechanics studies were only included if they analysed a powerlifting exercise (i.e., the free-weight barbell back squat, bench press, or deadlift) with loads ≥ 85% of participants’ 1RM to ensure the findings are relevant to the near-maximal loads lifted in competition.

Studies were excluded from the review if they used powerlifters as subjects but did not investigate powerlifting as a sport or the applied sport science or medicine of powerlifters. It should be noted that studies investigating physical qualities or outcomes of powerlifting that are not directly related to performance (e.g., genetic [39], cardiovascular [40–43], or bone [44, 45] characteristics) were not included, and psychology studies that investigated powerlifters from a clinical perspective (e.g., body image perceptions [46], muscle dysmorphia [47], exercise dependence [48], or disordered eating [49]) were excluded since they also may not directly relate to performance. For the same reason, interventions that used powerlifters as participants but did not investigate outcomes that directly impact powerlifting performance (e.g., changes in blood biochemistry following supplement consumption [50] or strength endurance but not maximum strength [51]) were excluded. Case studies investigating a single participant were also excluded since their findings may not apply to a wider population of powerlifters. Finally, biomechanics studies were excluded if participants used non-powerlifting equipment (e.g., the Smith machine [52]) or if the aim was to compare a powerlifting exercise with an exercise that uses non-powerlifting equipment (e.g., comparing the traditional and hexagonal barbell deadlifts [53]). Studies that investigated para powerlifting or included para powerlifters were evaluated according to the above criteria. These restrictions were applied to ensure that all included studies are relevant to individuals or organisations aiming to support powerlifting or para powerlifting performance or reduce injury. Conference proceedings and review articles were excluded and papers from all languages were eligible but excluded if translation to English could not be made. When authors could not be contacted to retrieve full texts where required, studies were excluded.

### Data Extraction

To establish the categories for classification, the research team reviewed the included studies and discussed the overarching topics of applied sport science and medicine. Studies were categorised into the agreed-upon topics based on their primary aim and outcome measures, with sub-categories identified where appropriate. Studies were only placed into a single category, even if secondary aims spanned multiple research topics. The title and year of publication, country of origin according to the lead author’s first affiliation, cohort investigated, and sample size of each study were extracted. Where specified by each study, data relating to the participants’ physical characteristics (sex, age, and body mass) and competitive characteristics (powerlifting or para powerlifting, level of competition, division, weight class, age category, and testing status) were also extracted. Finally, each study’s aims, outcome measures, and key findings relevant to the purpose of this review were extracted.

### Data Synthesis

Given that the purpose of a scoping review is to (1) map the extent, range, and nature of the literature on a topic, (2) summarise findings that are heterogeneous, and (3) identify gaps in the literature [37], no meta-analysis was performed. Study characteristics, aims, outcome measures, and findings were summarised with data presented as mean ± standard deviation (SD) where appropriate.

## Results and Discussion

**Fig. 1:**
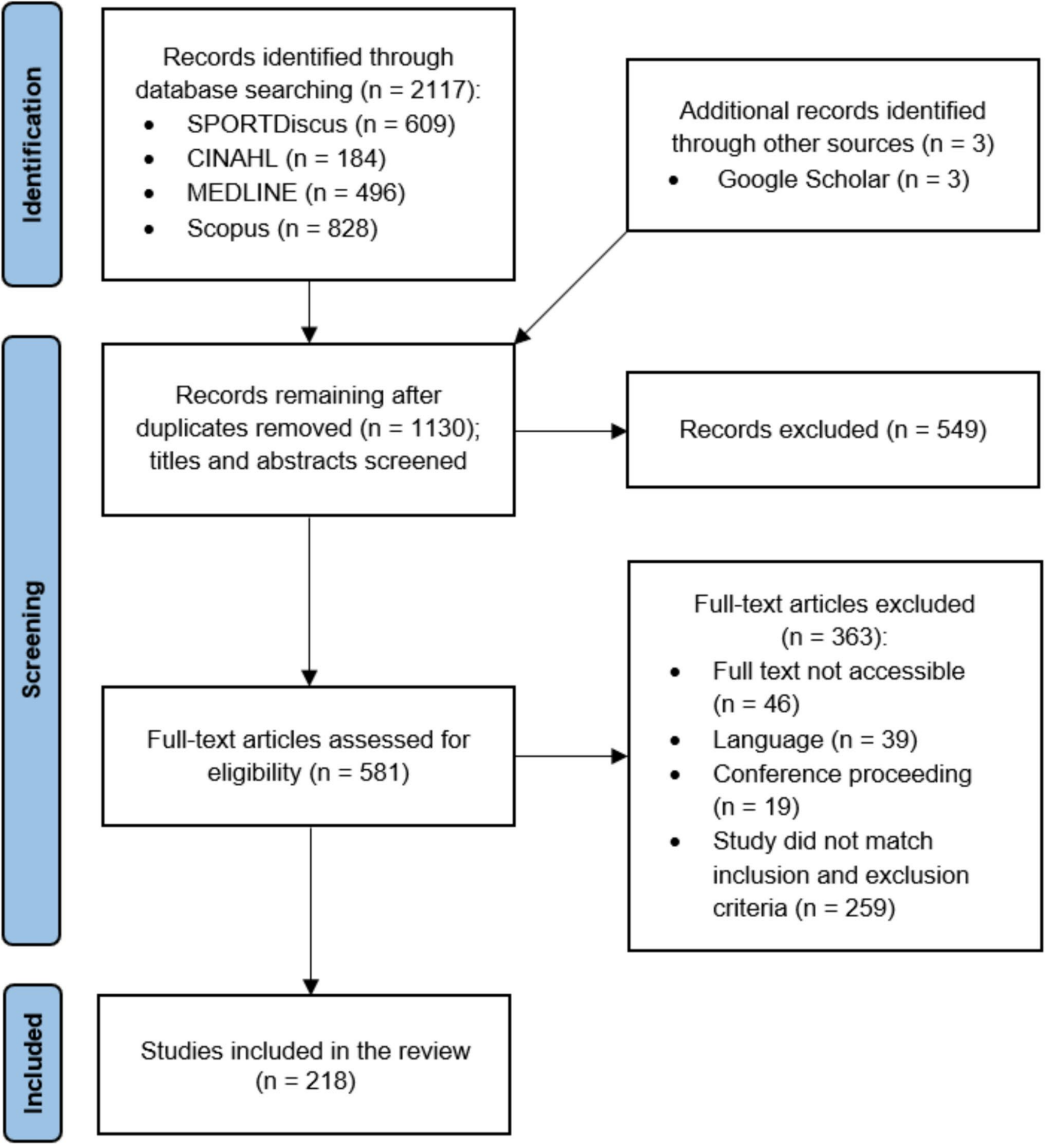
Flowchart of the selection process for studies eligible for inclusion

The database search identified 2117 articles, which were screened for eligibility after duplicates were removed. No additional eligible studies were identified through the manual searches of previous powerlifting reviews’ reference lists; however, three additional eligible studies were discovered through other sources during the writing process and were thus included [54–56]. The flow of articles from identification to inclusion is shown in Fig. 1, with 218 studies ultimately included in this review.

### General Characteristics of the Studies

#### Research Topics and Year of Publication

**Fig. 2:**
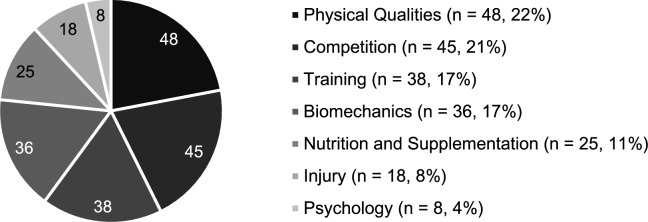
Sport science and medicine topics of the included studies

Seven distinct sport science and medicine categories were identified across the 218 studies included in this review: biomechanics (*n* = 36, 17%), training (*n* = 38, 17%), competition (*n* = 45, 21%), physical qualities (*n* = 48, 22%), injury (*n* = 18, 8%), psychology (*n* = 8, 4%), and nutrition and supplementation (*n* = 25, 11%) (Fig. 2).

**Fig. 3:**
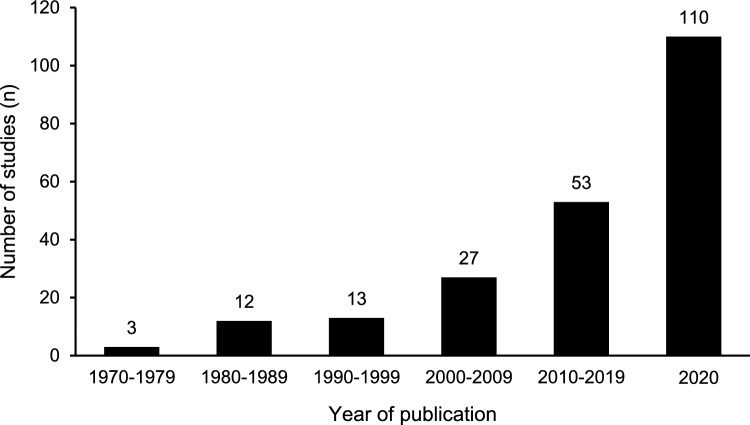
The number of publications from each decade of powerlifting research from the earliest records to June 2025

The exponential increase in powerlifting and para powerlifting literature in the last half-century is illustrated in Fig. 3, with 50% of studies (*n* = 110) published in 2020 or later. Additionally, Table 2 contains a breakdown of publications over time within each sport science and medicine category. Biomechanics and physical qualities were the earliest areas of research, comprising 100% of studies published prior to 1980 [4, 5, 57]. Notably, the first investigation of powerlifters’ training was not published until 2009 [58]; in the 2020s so far, training has been the second most studied area (*n* = 24, 22%), with competition (*n* = 26, 24%) and physical qualities (*n* = 19, 17%) placing first and third, respectively. Finally, every topic has had more publications in 2020 or later than in any previous decade, except for psychology, which was investigated four times in the 2010s [59–62] but only twice since 2020 [63, 64].

**Table 2:** The publication year of studies within each category

Research topic	Studies (n) per decade of publication					
	1970–1979	1980–1989	1990–1999	2000–2009	2010–2019	≥ 2020
Biomechanics	2	6	3	4	5	16
Training	0	0	0	1	13	24
Competition	0	0	2	6	11	26
Physical qualities	1	4	2	9	13	19
Injury	0	1	1	2	5	9
Psychology	0	0	1	1	4	2
Nutrition and supplementation	0	1	4	4	2	14

#### Geography of Studies and Bibliographic Network

**Fig. 4:**
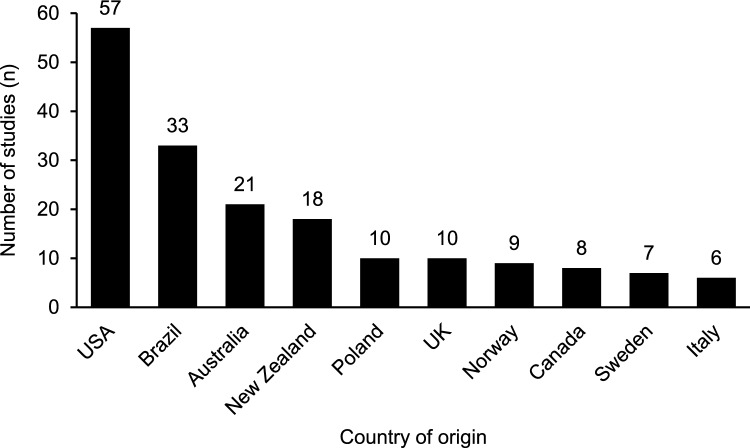
The number of publications originating from the top 10 most prolific countries in powerlifting research (articles are attributed to the first affiliation of the lead author)

**Fig. 5:**
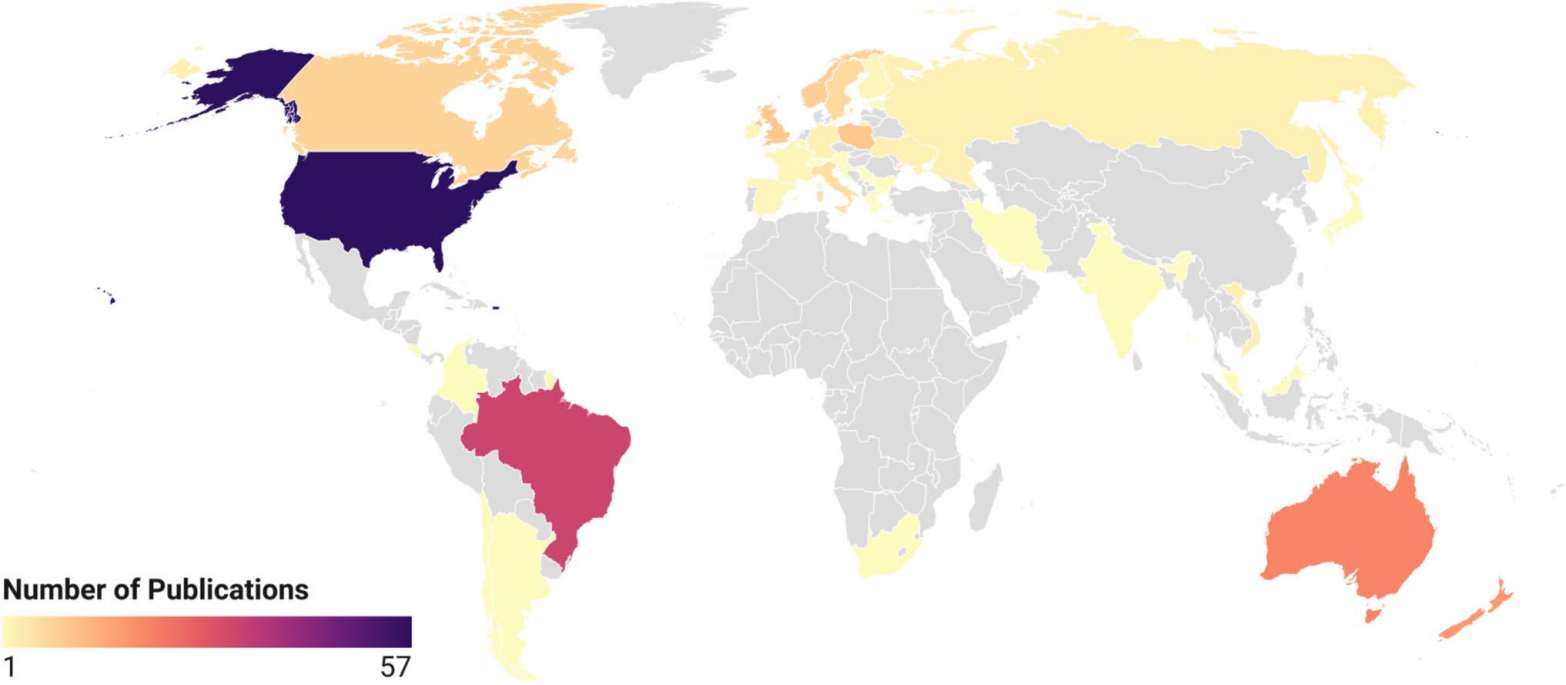
Choropleth of articles originating from each country (articles are attributed to the first affiliation of the lead author)

**Fig. 6:**
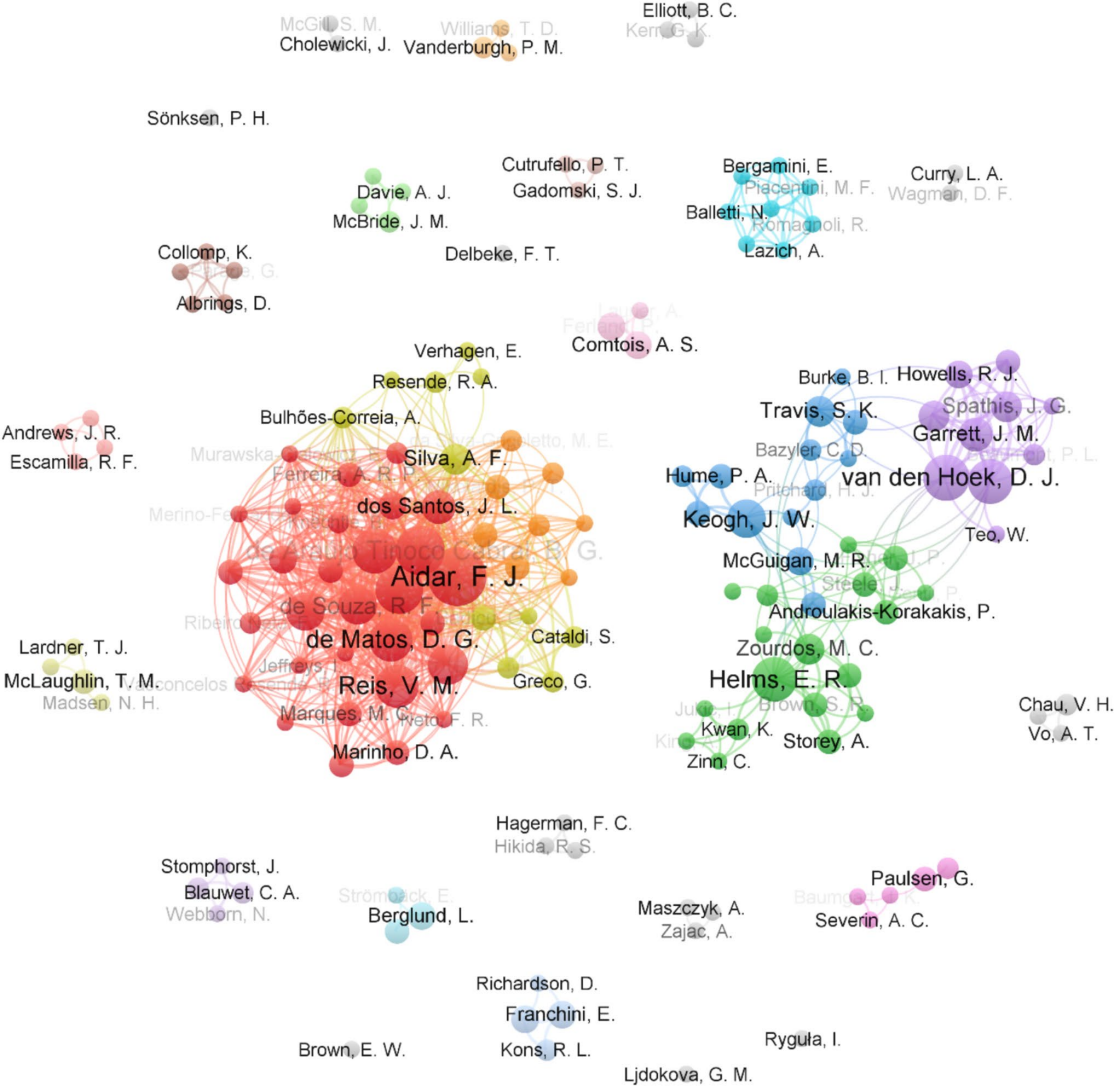
Co-authorship network of researchers who had two or more publications included in this review. Nodes and names are scaled relative to that researcher’s number of powerlifting publications, with the names of authors who had more publications presented at the front (i.e., greater opacity); nodes are linked if those authors have previously collaborated; and closer node proximity represents a greater number of collaborations between those authors

Investigations from researchers who listed their primary research institute as being in the USA published the most studies of those included in this review (*n* = 57, 26%). Brazil was the second most prolific country (*n* = 33, 15%), of which all but two publications [65, 66] investigated para powerlifting. Figure 4 shows the 10 countries with the most articles included in this review, rounded out by Australia (*n* = 21, 10%), New Zealand (*n* = 18, 8%), Poland (*n* = 10, 5%), the UK (*n* = 10, 5%), Norway (*n* = 9, 4%), Canada (*n* = 8, 4%), Sweden (*n* = 7, 3%), and Italy (*n* = 6, 3%). Figure 5 provides an illustration of the geographical origin of publications included in this review. Additionally, Fig. 6 is a network of authors active in powerlifting or para powerlifting research, allowing for visualisation of the most prolific researchers and their collaborative interactions [67].

#### Cohorts Investigated

Of the 218 articles included in this review, 173 studies (79%) did not include para powerlifters, 43 studies (20%) investigated para powerlifters exclusively, and two studies compared powerlifting and para powerlifting athletes [68, 69]. Additionally, 90 studies (41%) investigated a mixed cohort of males and females; 48 studies (22%) did not state the sex of participants; 73 studies (33%) only investigated male participants; and notably, only seven studies (3%) investigated female participants exclusively [56, 70–75].

While many studies did not specify participants’ level of competition (*n* = 99, 45%), 40 studies (18%) stated that they investigated national-level athletes, and 22 studies (10%) stated that they investigated world-class athletes competing internationally. Furthermore, although this term may not have a widely agreed-upon definition, 13 studies (6%) simply stated that participants were ‘elite’. One study investigated high school students at an interscholastic competition [76], six studies (3%) investigated collegiate athletes [26, 61, 77–80], and 37 studies (17%) stated that they investigated a mixed cohort including athletes from multiple levels of competition. Additionally, most studies did not specify the age category of participants (*n* = 185, 85%). Of those that did, five studies stated that they investigated teenage athletes [76, 81–84]; three stated that they investigated ‘junior’ athletes [85–87] (19–22 years of age in the IPF [7]); four investigated athletes in the ‘open’ category [31, 88–90] (23–39 years of age); two investigated ‘masters’ athletes [91, 92] (40 years of age or older); and 19 studies (9%) investigated a mixed cohort composed of multiple age categories.

Most powerlifting studies did not specify participants’ competitive division (i.e., raw, wraps, or equipped; *n* = 132). While no studies stated that all participants competed in the wraps or equipped divisions, 34 studies specified that participants were in the raw division, and seven studies stated that they investigated a mixed cohort of multiple divisions. Finally, of the 218 studies included in this review, most did not explicitly disclose participants’ drug-use status or indicate that they competed in federations with exclusively drug-tested competitions (*n* = 183, 84%). Thirty-five studies did state that participants were not using PEDs or that they competed in tested federations like the IPF (although one of these studies was an exploration of steroid use by tested powerlifters [93]); however, no studies stated that participants were not drug-tested in competition.

### Biomechanics

Thirty-six of the included studies investigated the biomechanics of one or more of the powerlifting lifts as their primary aim (Supplementary Table S1, see electronic supplementary material [ESM]). Biomechanics studies analysed kinematics (e.g., joint angles), kinetics (e.g., ground reaction forces), electromyography (EMG), or a combination of these outcome measures. Most studies exclusively examined either the powerlifting squat, bench press, or deadlift, or the para powerlifting bench press. However, one study compared bench press biomechanics between powerlifters and para powerlifters [69]; one study compared the kinematics of the squat and the conventional deadlift during competition [25]; and Helms et al. [94] established relationships between rating of perceived exertion (RPE) and barbell velocity in the squat, bench press, and deadlift. The majority of biomechanics studies only included male participants (*n* = 20, 56%), while the remaining studies included a mixed-sex cohort (*n* = 4, 11%) or did not specify the sex of participants (*n* = 12, 33%). Eight studies (22%) did not report participants’ level of competition; those that did, analysed the biomechanics of national-level athletes (*n* = 12, 33%) [5, 57, 91, 92, 95–102], world-class athletes (*n* = 3, 8%) [103–105], ‘elite’ athletes (*n* = 4, 11%) [106–109], or a mixed-level cohort (*n* = 9, 25%).

#### Squat

Eight of the included biomechanics studies investigated the squat exclusively. McLaughlin et al. produced both a kinematic [5] and a kinetic [57] model of squat performance during a national-level competition. Two studies compared the low-bar and high-bar back squats and observed greater quadriceps activation in the high-bar position [103, 110]. Additionally, one study compared biomechanical parameters between varying stance widths and found greater knee and hip moments during squats with a wide stance compared with a narrow stance [91]. Pürzel et al. [101] modelled muscle force dynamics during squats from 70 to 90% 1RM, finding that the gluteus maximus and semitendinosus exhibited the greatest relative increase in force with increasing intensity. Significant kinematic differences have been observed between novice, high school, and collegiate powerlifters’ squats [111]. Finally, supportive equipment (i.e., an elastic squat suit) has been shown to increase squat velocity and power [112].

#### Bench Press

Six studies analysed the biomechanics of the powerlifting bench press only. The ‘sticking point’ (the moment of slowest upward velocity during the concentric phase) seems to be of particular interest in bench press research: Madsen and McLaughlin first identified this phenomenon in 1984 [113]; the mechanisms responsible for the sticking point were investigated further in 1989 using EMG [107]; and three grip widths were later shown to all produce a clear sticking point at different joint angles [98]. One study investigated the biomechanical and structural determinants of 1RM bench press performance, finding that anthropometric measures (e.g., lean body mass) were better predictors of performance than technical factors (e.g., bar path or joint angles) [114]. One study characterised the kinematics and kinetics of the bench press performed by heavyweight powerlifters [109]. Lastly, a study compared kinematic and kinetic parameters between submaximal and maximal bench press loads, observing that the bar path and force profile differed significantly [115]. Notably, four of the included studies on the powerlifting bench press were produced in the 1980s [107, 109, 113, 115]; since then, only two studies have been published [98, 114].

#### Deadlift

Six of the included studies solely investigated deadlift biomechanics. Two studies aimed to compare the conventional and sumo style deadlifts: one found significantly different ankle and knee (but not hip) moments between styles [92], while the other found postural and mechanical differences between styles but no difference in where the sticking point occurred [116]. One study characterised the biomechanics of the deadlift when performed by adolescent powerlifters in competition [82], while the remaining three studies focused on the lower back during maximal deadlifts. Two of these studies estimated the loads placed upon the lumbar spine in competition, observing vertebral compressive forces ranging from 18.4 to 36.2 kN [104] and showing that the sumo deadlift reduced shear force compared with the conventional deadlift [95]. Finally, Cholewicki and McGill [117] found that the posterior longitudinal ligament does not contribute strongly to resisting the trunk flexion moment during the deadlift.

#### Para Powerlifting

Of the included studies, 13 exclusively investigated para powerlifters’ bench press biomechanics. Many of these studies compared biomechanical outcome measures between participants or techniques, including different grip widths [97]; different eccentric tempo durations [102]; regional- versus national-level athletes [118]; the arched versus flat-back bench press [119]; partial versus full range of motion [99]; and athletes with spinal cord injuries versus other disabilities [120, 121]. Additionally, two studies compared strength indicators between tethered (where athletes are fixed to the bench with stabilising leg straps) and untethered conditions [108, 120]. One study investigated the sticking point in para powerlifters [122], another characterised muscle activation during each phase of the bench press [96], and two methods of 1RM prediction based on the force–velocity relationship have been assessed [106]. Lastly, asymmetrical elbow extension has been observed in national-level para powerlifters [100], while world-class athletes have been found to exhibit symmetrical upper limb muscle activation but asymmetrical external oblique activation during maximal lifts [105].

#### Biomechanics Summary and Applications

Studies have characterised the kinematics, kinetics, and muscle activity associated with the powerlifting squat, bench press, and deadlift, as well as the bench press performed by para powerlifters. These studies have included participants of various weight classes, ages, and levels of competition, but no biomechanics studies have investigated female athletes exclusively. Biomechanical outcome measures have been compared between lifts (e.g., squat versus deadlift [25]), lift variations (e.g., conventional versus sumo style deadlifts [92, 116]), lifting techniques (e.g., grip width in the bench press [97, 98]), equipment conditions, and intensities. These comparisons can inform the training and competition practices employed by athletes and coaches to achieve desired outcomes and maximise performance. Additionally, research areas of particular interest have included lumbar spine mechanics during the deadlift [95, 104, 117] and the bench press sticking point in both powerlifters [98, 107, 113] and para powerlifters [122]. However, updated research on the powerlifting bench press is warranted due to a lack of recent publications.

### Training

Thirty-eight studies characterised the training of powerlifters or para powerlifters or investigated the effects of a training intervention (Supplementary Table S2, see ESM). Several studies used questionnaires to collect self-reported descriptions of training practices from participants, while others evaluated changes across a range of outcome measures (e.g., biomechanical, physical, psychological, or performance measures) in response to various short-term training practices or long-term training programmes. Additionally, three studies investigated the process of ‘tapering’ (the final stage of competition preparation where overall load is reduced to achieve peak performance). Specifically, one study tracked bench press strength throughout overload and taper microcycles [123], one compared ‘step’ and ‘exponential’ tapering methods [124], and one compared 2 days and 4 days of training cessation after a taper [12]. Helms et al. found that powerlifters can accurately self-select loads to achieve a target RPE [125] and that this may allow for effective autoregulation of training volume within a periodised programme [126]. Finally, a study investigated the relationships between online coaching characteristics, athlete satisfaction, and performance, observing a positive association between the duration of the coach–athlete relationship and the athlete’s powerlifting total [127]. Seven of the included studies (18%) did not specify participants’ sex, 16 studies (42%) had a mixed-sex cohort, and 14 studies (37%) solely included males. Only one study investigated female athletes’ training exclusively [74]. Nine of the included studies (24%) investigated para powerlifting training [128–136]. Seven studies (18%) stated that all participants competed in drug-tested competitions [80, 125–127, 137–139], while the rest did not specify whether participants were tested or untested. Training studies either did not report participants’ level of competition (*n* = 15, 39%), included a mixed-level cohort (*n* = 7, 18%), or investigated collegiate (*n* = 3, 8%) [78–80], national-level (*n* = 9, 24%) [130–134, 136, 138, 140, 141], international-level (*n* = 1, 3%) [58], or ‘elite’ athletes (*n* = 3, 8%) [65, 142, 143].

#### Training Practices

Six studies used questionnaires to characterise training practices employed by powerlifters. Half of these studies (*n* = 3) explored tapering strategies, all similarly observing reductions in training volume and varied manipulations of training intensity during the tapering period [140, 143, 144]. Two studies investigated ‘contemporary’ training practices employed by powerlifters, with considerable proportions of British [58] and Norwegian athletes [29] reporting the use of elastic bands (39% and 76%, respectively), chains (57% and 63%), and Olympic weightlifting exercises (69% and 33%). Finally, Spence et al. [139] characterised the stretching practices of powerlifters competing in the IPF. Static or dynamic stretching was reported by 52% of participants, with 78% of them stating that they stretch before training, 44% stretch after training, and 54% stretch outside of training. Notably, none of the included studies characterised training practices employed by para powerlifters.

#### Acute Training Effects

Ten studies evaluated the acute effects of a short-term powerlifting training practice or intervention (i.e., where outcome measures were obtained up to 48 h after each testing session and testing was performed over 3 weeks or less). One study investigated the effects of whole-body vibration on strength parameters [138], while another compared endocrine responses to high intensity squat sets of different volumes [145]. The remaining eight studies examined acute training effects in para powerlifters. Two studies by Resende et al. measured the impacts of different warm-up methods on strength and core temperature [132] or skin temperature [131]. Stieler et al. [134] analysed the relationships between para powerlifting training and sleep. One study measured post-training recovery markers after cold-water immersion, dry needling, and passive recovery [133], while another observed significant increases in strength after cold-water immersion [135]. Lastly, bench press strength and fatigue indicators have been compared when performed with and without elastic bands [128], chains [136], and supramaximal eccentric loads [130].

#### Chronic Training Effects

Sixteen studies investigated the outcomes of a long-term powerlifting training programme or intervention (i.e., one which was implemented over 4 weeks or more). One study measured the effects of a 16-week periodised training programme on maximal strength [65], a longitudinal study conducted yearly follow-ups to determine the effects of powerlifting training on strength and body composition [146], and one study compared physical and performance measures between different training phases [137]. Two studies assessed the efficacy of daily 1RM training with mixed findings [147, 148], while a ‘training methodology’ for female powerlifters was developed in another [74]. One publication explored the minimum effective training dose required to increase maximal strength through interviews, surveys, and structured training interventions [149]. Higher and lower volume and intensity programmes have been assessed in visually impaired powerlifters [141], while one study compared two different weekly configurations of hypertrophy-, strength-, and power-specific training sessions [80]. One study compared the effectiveness of traditional and cluster sets on increasing 1RM performance [150], while two studies investigated blood-flow restriction training with positive results [79, 142]. Ghigiarelli et al. [78] implemented a 6-week bench press programme using a modified barbell, while another study evaluated the effects of bench press training with supramaximal eccentric loads on biomechanical and performance measures [151]. Additionally, low-load high-volume bench press training may be an effective alternative to traditional high-load low-volume protocols for improving strength and structural adaptations [152]. Finally, only one of the included studies investigated the outcomes of a para powerlifting training programme, observing increased strength and mixed impacts on psychological questionnaires measuring stress, recovery, and mood state [129].

#### Training Summary and Applications

Training is the area of powerlifting and para powerlifting research that has grown most rapidly, from the first publication in 2009 [58] to the 38 articles included in this review. Some studies simply characterised powerlifters’ training, although no such studies have investigated para powerlifters. Many of the included studies evaluated the acute effects of various training practices (e.g., warm-up methods [131, 132]), allowing athletes and coaches to target desired short-term physiological responses. Additionally, numerous studies measured the chronic outcomes of different training programmes, further informing athletes and coaches on the most effective interventions to improve powerlifting performance. Interestingly, most studies on acute training effects investigated para powerlifters exclusively, while most chronic training studies only included powerlifters; as such, more research is required to balance the body of evidence around both sports. Finally, tapering strategies have been heavily researched [12, 123, 124, 140, 143, 144], while advanced methods of training (e.g., variable resistance [29, 58, 128, 136]) and recovery (e.g., cold-water immersion [133, 135]) have also been emphasised in the literature.

### Competition

Forty-five studies investigated powerlifting or para powerlifting competitions or the results of these competitions (Supplementary Table S3, see ESM). Most of these studies analysed competition data to identify physical, chronological, or strategic determinants of performance or to allow for comparison between athletes with differing characteristics (e.g., weight class or level of competition). Additionally, two studies by Le Panse et al. [153, 154] measured endocrine responses to powerlifting competitions, observing increased cortisol levels. Another study gathered expert opinions to evaluate the athlete classification systems used in para powerlifting, concluding that these systems do not currently meet Paralympic standards [155]. The majority of studies analysed competition data containing results from both male and female lifters (*n* = 38, 84%), while five studies only included male athletes’ results [77, 156–159] and one study trained a predictive machine learning model using results from female athletes exclusively [73]. Para powerlifting was investigated by 24% of the included studies (*n* = 11), while 34 studies solely analysed powerlifting competitions. Half of the powerlifting studies investigated raw competitions (*n* = 17), while the rest included multiple divisions (*n* = 5) or did not specify the competitive division (*n* = 12). Fifteen studies (33%) stated that the investigated competitions were drug-tested. Studies either did not report the level of competition (*n* = 22, 49%) or investigated competitions at the collegiate level (*n* = 1, 2%) [77], nationally (*n* = 2, 4%) [160, 161], internationally (*n* = 12, 27%), at the ‘elite’ level (*n* = 3, 7%) [153, 154, 162], or at multiple levels of competition (*n* = 5, 11%). Notably, 58% of the included studies (*n* = 26) were published in the 2020s, likely due in part to the recent emergence of open-source databases of competition results [163].

#### Competition Strategies

Six of the included studies investigated the impacts of strategic factors (e.g., weight selection) on competitive success. Travis et al. [90] characterised the weights selected by IPF lifters, observing that the opening attempt was typically around 91% of the third attempt. One study concluded that the weight and outcome of the opening squat was one of the most important correlates of performance [164], while another found that successful completion of eight or nine (out of nine) lifts significantly increased the likelihood of winning [31]. Two studies analysed a range of physical and strategic determinants of performance, with heavier first attempts shown to improve the odds of winning [165] and the number of successful squats and bench presses (but not deadlifts) associated with relative strength [161]. Finally, a study on para powerlifting competitions recommended that athletes should improve their ability to predict their competition 1RM, allowing for appropriate weight selections that increase the likelihood of completing each attempt successfully [32].

#### Performance Determinants

Seventeen studies analysed chronological or physical determinants of powerlifting or para powerlifting performance. Four of these studies aimed to characterise strength changes over the lifespan in powerlifters [166–168] or para powerlifters [162], while van den Hoek et al. [169] tracked powerlifting participation in Australia since 1968 and compared lifters’ results with age-matched strength norms. Four studies investigated performance progression throughout athletes’ competitive careers, comparing strength adaptations between sexes [170–172] or determining correlations between ‘training age’ and performance in para powerlifters [160]. Two studies evaluated the effects of competition frequency on performance, concluding that four competitions per year may be the upper limit for progression in powerlifters [173], while 21–31 weeks may be the optimal interval between competitions for para powerlifters [174]. Age and body mass have been analysed as determinants of relative strength [175], while the factors influencing powerlifting performance have been described as a self-organised complex system [159]. Additionally, three of the included studies were able to successfully predict competition performance based on age, body mass, and previous competitive results using machine learning [73, 156, 157]. Lastly, one study investigated the prevalence and determinants of failed attempts during international para powerlifting competitions [176].

#### Comparison and Interpretation of Competition Results

Nineteen studies compared the results achieved by athletes with different characteristics or assessed systems used to compare powerlifters or para powerlifters between categories. One study evaluated differences in strength performance between novice and elite athletes [177], while another compared male versus female and raw versus equipped lifters [178]. Additionally, supportive equipment has been shown to significantly improve squat and bench press (but not deadlift) world records [6], while knee wraps have been found to only benefit powerlifters in the heaviest weight classes [179]. Two studies primarily aimed to investigate sex differences in powerlifting performance, observing that males in the lightest weight class largely outperform heavier females [180] and that strength differences may be greater in the open division than the teenage division [181]. One study compared relative strength between weight classes, while another presented normative data for the squat, bench press, and deadlift in drug-tested powerlifters based on sex, age, and weight class [9]. Six studies evaluated mathematical methods used to adjust powerlifting results based on body mass or other factors [10, 158, 182–185]. Of these, one study attempted to validate the Wilks formula [185], while another compared the effectiveness of the Wilks formula and the newer IPF formula [10]. Three para powerlifting studies investigated the performance impacts of numerous factors including the origin of impairment (e.g., acquired or congenital), with mixed findings [85, 186, 187], while another compared para powerlifters’ results between different categories [188]. Finally, van den Hoek et al. [68] compared IPF bench press world records with World Para Powerlifting world records, finding that many para powerlifters are stronger than their powerlifting counterparts.

#### Competition Summary and Applications

Research into powerlifting and para powerlifting competitions is accelerating, with more than half of the included studies published in 2020 or later, likely driven by the unprecedented availability of public repositories of competition results [163]. Most studies analysed data from these sources to identify competitive strategies, strength determinants, or differences between athletes, while two articles detailed endocrine responses to powerlifting competitions [153, 154] and another critiqued the athlete classification systems in para powerlifting [155]. Studies determining the most effective competition strategies or the impacts of factors like age, sex, and competition frequency on performance may be of interest to athletes and coaches, while studies comparing the results of lifters with different characteristics allow athletes to gauge their strength relative to others. Finally, studies investigating various methods of normalising results (e.g., the Wilks formula [10, 185]) can assist policymakers to implement evidence-based systems to fairly determine competition winners.

### Physical Qualities

Forty-eight studies characterised the morphological, physiological, or performance-based attributes of powerlifters or para powerlifters or associated these characteristics with performance (Supplementary Table S4, see ESM). Most of these studies primarily aimed to investigate anthropometry (e.g., limb lengths or circumferences), body composition (e.g., lean mass or bone mineral content), muscle characteristics (e.g., architecture or fibre type), or physical performance (e.g., strength, power, or flexibility) in powerlifters or para powerlifters. Additionally, two studies analysed a set of post-competition blood tests taken from 693 athletes in various sports, observing low testosterone levels in the 18 male powerlifters included in this sample [54, 189]. One study characterised powerlifters of different skill levels across a range of structural (e.g., anthropometry) and functional (e.g., upper body strength) parameters [190], while another compared powerlifters, bodybuilders, and wrestlers across structural and functional measures [28]. Two studies aimed to find the set of physical attributes most strongly correlated with powerlifting performance, both identifying the same nine variables that were significantly associated with strength, with chest circumference being the strongest correlate [86, 87]. One study monitored body composition, muscle architecture, and strength indicators over a training period, concluding that increases in lean body mass may dictate improvements in 1RM performance [191]. Finally, two studies investigated stress urinary incontinence in female powerlifters. Wikander et al. [70] found that urinary incontinence was prevalent in this cohort and that women who were confident in performing pelvic floor exercises generally experienced less severe incontinence, while another study aimed to correlate Incontinence Severity Index scores with musculoskeletal examination data [75]. More than half of the studies evaluating physical qualities only included males (*n* = 25, 52%), while 12 studies did not specify the sex of participants (25%), six included a mixed-sex cohort (13%), and five investigated females exclusively (10%) [56, 70–72, 75], comprising over 70% of the female-only studies included in this review. Only three of the studies in this category stated that participants were drug-tested [66, 192, 193]; furthermore, only one study investigated para powerlifters’ physical attributes [194].

#### Anthropometry and Body Composition

Thirteen studies primarily evaluated the anthropometry or body composition of powerlifters or para powerlifters. Ferland et al. investigated the relationships between body composition (obtained via dual-energy X-ray absorptiometry) and strength [195] and the relationships between anthropometric measurements and strength [196]. Three more studies aimed to identify the anthropometric variables most strongly associated with powerlifting performance, finding a range of measurements significantly correlated with strength in the bench press [197] or in all three lifts [198, 199]. Conversely, three similar studies did not find strong relationships between any anthropometric variables (except body mass) and strength in powerlifters [76, 83, 200]. Additionally, one study observed significant correlations between several anthropometric measurements (e.g., arm circumference) and para powerlifting performance [194]. Three studies anthropometrically characterised powerlifters of varying body mass, collectively finding that heavier athletes possessed significantly greater estimated muscle mass [27, 88, 201] (which is strongly associated with powerlifting performance [195]). Finally, Keogh et al. [202] compared the anthropometric profiles of male and female powerlifters, observing sexual dimorphism in absolute measurements like limb girths but less dimorphism in proportional characteristics and adiposity.

#### Muscle Characteristics

Eleven studies analysed specific muscle attributes in powerlifters. Two studies by Prince et al. [4, 203] compared muscle fibre qualities (e.g., fibre type) between powerlifters, long-distance runners, and non-athletic controls, while another study found that the percentage area of type IIA and type IIB fibres contributed strongly to powerlifting performance [204]. Myosin heavy chain composition and creatine analogues have been compared between powerlifters and sedentary controls [205]; greater fascicle lengths have been associated with improved powerlifting performance [206]; and titin characteristics have been investigated in powerlifters, weightlifters, sprinters, and non-athletes [192]. One study compared hip muscle texture between powerlifters and other athletes using magnetic resonance imaging (MRI) [71], while another used MRI to assess training-associated changes in the lower back muscles of powerlifters, endurance athletes, and untrained controls [207]. Sakakibara et al. [208] compared transversus abdominis contractility during regional- and national-level powerlifters’ deadlifts using ultrasound imaging and found no difference. Thigh muscle cross-sectional area has been shown to correlate with squat performance in bodybuilders but not powerlifters or weightlifters, despite the greater strength exhibited by the powerlifters and weightlifters [209]. Finally, one study observed more pronounced protein synthesis signalling responses in the skeletal muscles of powerlifters compared with untrained controls after a bout of resistance training [210].

#### Physical Performance

Fifteen studies primarily investigated muscular performance or flexibility measures in powerlifters. One study compared strength and power indicators between powerlifters, weightlifters, and sprinters [211], while another found that powerlifters had the highest maximal handgrip force values compared with athletes from 10 other sports [212], likely due to the grip strength requirements of the deadlift [213]. Furthermore, grip strength has been shown to correlate more strongly with squat and deadlift performance than bench press performance in female powerlifters [56]; conversely, in another study, seated handgrip strength was most closely associated with bench press 1RM in males [193]. Studies have investigated isokinetic contractions of the knee extensors and shoulder rotators, observing that powerlifters are stronger but fatigue faster than untrained controls during repetitive knee extensions [214] and that powerlifters have stronger shoulder rotators than controls while also exhibiting bilateral asymmetry [66]. One study found that isolated lumbar extension strength does not differ between powerlifters and recreationally trained controls [215], while another observed stronger trunk flexion in powerlifters compared with endurance athletes and inactive controls but found no trunk extension strength differences between powerlifters and endurance athletes [216]. Strength imbalances between agonist and antagonist muscles have been identified at the shoulder and knee in powerlifters [217], while greater bilateral symmetry of lower limb force production has been exhibited by powerlifters compared with field jumpers [218]. One study evaluated knee stability in powerlifters, weightlifters, and untrained controls [219], while two studies observed reduced flexibility across numerous joints in powerlifters compared with control groups [220, 221]. Similarly, Spence et al. found limited flexibility in male powerlifters [222] but concluded in another study that range of motion was not lower in female powerlifters compared with recreationally trained controls [72].

#### Physical Qualities Summary and Applications

Research that primarily investigated physical qualities outnumbered all other topics, with 48 articles included in this review. Studies have mainly assessed the anthropometry, body composition, muscle characteristics, or physical performance of powerlifters, with additional studies evaluating the endocrine profiles of athletes [54, 189] and investigating stress urinary incontinence in females [70, 75]. Para powerlifters were only included in one study, which correlated anthropometric measurements with bench press performance [194]. No studies have investigated muscle characteristics in para powerlifters; in powerlifters, studies have analysed muscle qualities such as fibre types [4, 28, 203–205] and fascicle lengths [206], revealing physiological adaptations associated with powerlifting participation. Similarly, research into the physical performance of powerlifters demonstrates the functional attributes either induced by powerlifting or predisposing participants to the sport, while studies concerning anthropometry and body composition can inform athletes and coaches on which morphological features should be targeted to maximise performance.

### Injury

#### Epidemiology

**Fig. 7:**
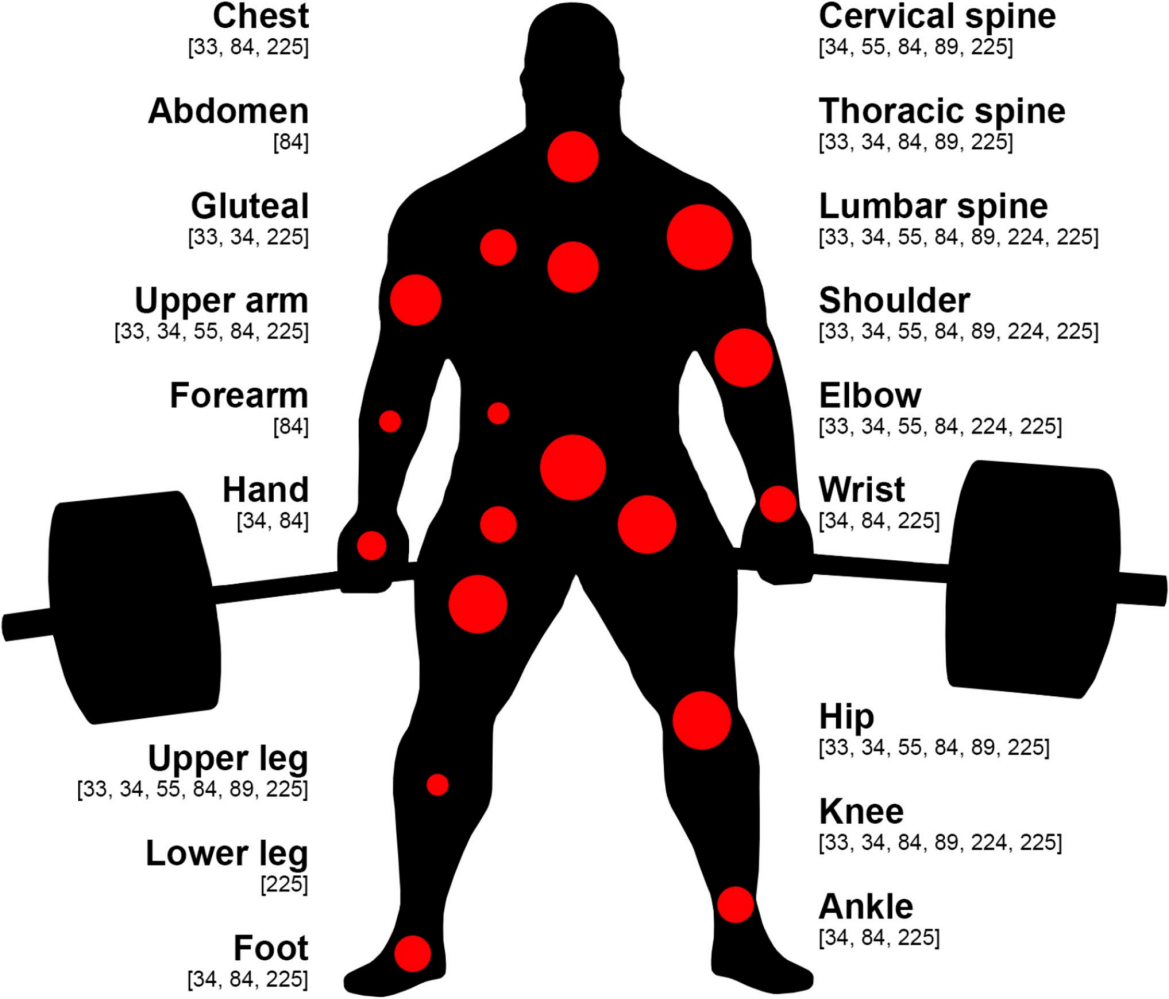
Powerlifting studies included in this review that reported injury rates at each anatomical region. Anatomical region markers are scaled relative to the number of studies that reported injury rates at that site, while the numbers in brackets represent in-text references

Of the 18 studies that primarily focused on injuries (Supplementary Table S5, see ESM), 13 investigated injury epidemiology (e.g., incidence, prevalence, or characteristics). Of these, 11 included a mixed-sex cohort, while one study investigated male athletes exclusively [84] and one did not specify participants’ sex [223]. Seven studies used questionnaires to explore the injury history of powerlifters [33, 34, 55, 84, 89, 224, 225]; Fig. 7 illustrates the body regions at which each of these studies reported injuries. Two of these studies stated that all participants were drug-tested [33, 55], while one only included teenage powerlifters [84], and another assessed injuries in visually impaired athletes [224]. The most commonly injured body regions appear to be the shoulder (up to 73% of lifters [224]), lower back (up to 67% [84]), and knee (up to 39% [34]). Incidence rates have been reported between 1.0 [34] and 4.4 [33] injuries per 1000 h of training. Additionally, six studies investigated para powerlifting injury epidemiology. One of these studies evaluated para powerlifting injuries at the London 2012 Paralympic Games, observing that most were caused by chronic overuse and that the shoulder/clavicle was the most commonly injured site [226]. Furthermore, two studies investigated the Rio 2016 Paralympic Games [35, 227]. One of these found that chronic overuse injuries were the most prevalent and that the shoulder was the most injured region [227]; the other study monitored injuries across all sports, finding that the disciplines most prone to muscle injuries were para powerlifting and athletics, while tendon injuries were most common in para powerlifters [35]. Analysis of Paralympic Games results from 1960 to 2022 showed that para powerlifters with acquired spinal cord injuries win proportionally fewer medals than those with other disabilities [223]. Finally, two studies investigated injuries across multiple sports at Brazilian Paralympic training facilities [36, 228]. Para powerlifting was the sport with the highest injury rate in one of these studies, with the shoulder most commonly affected [36], while Resende et al. [228] found that para powerlifters were 66% less likely to experience sudden-onset injuries than para athletics competitors (possibly due to the highly predictable nature of para powerlifting).

#### Lifting Techniques

Only five of the included studies investigated lifting techniques from the perspectives of injury prevention or risk assessment. Sjöberg et al. [229] developed lifting technique evaluation protocols for the squat and deadlift, involving examination of specific aspects of technique (e.g., movement symmetry or lower back rounding) with promising validity and reliability. Similarly, a clinical assessment protocol for diagnosing lower back and hip pain in powerlifters has been developed and successfully evaluated for feasibility [230]. Aasa and Berglund [231] paired four powerlifters who had lower back pain with four pain-free controls before assessing each participant via physical performance tests and MRI. The lifters with and without lower back pain exhibited similar functional impairments (e.g., range of motion limitations) and patho-anatomical findings (e.g., intervertebral disc degeneration); however, those without pain tended to have a more ‘ideal’ squatting technique according to the aforementioned technique evaluation protocol [229]. Another study compared lumbopelvic movement control between powerlifters with and without lower back pain, finding no significant differences [232]. Additionally, Marotta et al. [233] evaluated the ‘flexion relaxation phenomenon’ as a screening tool for lower back pain risk in powerlifters, concluding that the flexion relaxation ratio might be a useful risk indicator.

#### Injury Summary and Applications

Despite their unique epidemiology, powerlifting and para powerlifting injuries appear under-researched. Powerlifting injuries most commonly affect the shoulder, lower back, and knee [33, 34] at a rate of up to 4.4 injuries per 1000 h of training [33], while tendon injuries may be more prevalent in para powerlifters than other para athletes [35]. Studies have retrospectively investigated the incidence, prevalence, or characteristics of injuries in powerlifters or para powerlifters; however, no prospective injury studies have been completed, providing an opportunity for future research in this area to identify physical or behavioural factors associated with injury. Furthermore, only five articles have evaluated participants’ lifting techniques to identify risk factors [231–233] or develop standardised assessment protocols [229, 230], and no such studies have included para powerlifters. Further research in this area could assist athletes and coaches in achieving optimal movement patterns that maximise performance while avoiding injury.

### Psychology

Eight studies explored the psychology of powerlifters from a performance perspective (Supplementary Table S6, see ESM). Of these, two studies investigated collegiate powerlifters [26, 61], one investigated national-level athletes [234], and two included a mixed-level cohort [62, 64], while the rest did not specify participants’ level of competition. Notably, no studies investigated the psychology of para powerlifters. One study associated the results of an attentional style test with powerlifting performance, finding that less successful lifters scored higher on the ‘Narrowing’ and ‘Broad Internal’ subscales compared with successful lifters [26]. Another study evaluated the effects of competitive trait anxiety on performance, concluding that anxiety may have negatively impacted some participants in competition and that powerlifters may benefit from anxiety-reducing interventions [61]. Two studies by Ljdokova et al. explored the confounding factors influencing powerlifters, determining that training and competition performance are primarily affected by injuries and emotional stress, respectively [59], while the strongest psychological factor influencing both training and competition performance is emotional stress in females and injuries in males [60]. One study investigated the expectancy effects of anabolic steroids by administering a placebo to a group of national-level powerlifters, observing notable improvements in performance despite the lack of physiological changes [234]. Another study assessed through interviews with former powerlifters how ‘situated learning’ (where an individual is immersed in a contextual environment) leads to anabolic steroid use, describing a ‘doping culture’ in the powerlifting community [63]. Finally, two studies used electroencephalography to measure brain activity during the bench press. One of these studies investigated the effects of biofeedback training on motivation and performance [64], while the other found that novices display greater neural activity at relatively light loads compared with elite lifters [62].

#### Psychology Summary and Applications

Psychology is the least researched topic in powerlifters, with eight articles included in this review, of which only two were published in the 2020s [63, 64]. No studies primarily investigating psychology have included para powerlifters. Half of the included studies associated psychological factors such as attentional style [26] and competitive trait anxiety [61] with performance. Of the remaining articles, one investigated the expectancy effects of anabolic steroids by administering a placebo [234]; one assessed environmental factors leading to PED use [63]; and two studies measured brain activity during the bench press using electroencephalography [62, 64]. Future research could expand on the body of evidence surrounding performance psychology, informing athletes and coaches on the most effective psychological strategies (e.g., to improve training adherence or maximise competition performance).

### Nutrition and Supplementation

Twenty-five studies investigated the dietary practices of powerlifters or the use of legal or illegal ergogenic aids (Supplementary Table S7, see ESM). Many of these studies used questionnaires to characterise the nutritional or pharmacological practices employed by athletes, while others assessed performance indicators in response to a nutritional or pharmacological intervention. Five studies (20%) investigated males exclusively [235–239], 10 studies (40%) included a mixed-sex cohort, and 10 studies (40%) did not specify the sex of participants. Additionally, while most studies did not specify participants’ competitive level (*n* = 16, 64%), three studies investigated national-level lifters [93, 240, 241], one investigated world-class athletes [242], three included a mixed-level cohort [236, 243, 244], and two studied ‘elite’ powerlifters [30, 245]. Lastly, four studies (16%) investigated the effects of supplementation or pharmacological interventions in para powerlifters [235, 236, 240, 246].

#### Diet and Ergogenic Supplements

Twenty studies characterised the dietary practices or legal ergogenic supplements used by powerlifters or evaluated the effects of a nutritional or pharmacological intervention. King et al. used a survey to investigate the general nutrition practices [247] and peri-workout nutrition practices [248] of powerlifters, while the effects of nutrition knowledge, sex, and training period (i.e., off-season versus pre-competition) on dietary adequacy have been assessed [244]. Since 2022, five studies have investigated rapid weight loss strategies employed by powerlifters to ‘cut weight’ into a competitive weight class [30, 81, 243, 249, 250]. The amount of weight lost during this process is typically around 3% of body mass [30, 249], and athletes commonly achieve this through water loading, fluid restriction, and gradual dieting [30, 249, 250]. Three of the included studies explored nutritional supplement use across a range of sports, finding that supplementation was most prevalent in powerlifters (employed by 79%) [238], powerlifters were the most frequent users of creatine and caffeine [251], and masters powerlifters had higher zinc intakes compared with other athletes [252]. Additionally, one study measured caffeine concentrations in the urine of athletes from different sports, observing that the greatest concentrations originated from powerlifters [253]. One study found that 74% of Australian powerlifters had used creatine [241], while three studies assessed the performance effects of creatine supplementation in powerlifters [237, 254] or para powerlifters [240] with largely positive results. The use of ammonia inhalants was reported by 49% of the 256 respondents to an international survey, with ammonia more commonly inhaled prior to the deadlift than the squat or bench press [239]. Finally, three studies observed some positive effects of ibuprofen intake on performance and recovery indicators in para powerlifters [235, 236, 246].

#### Performance-Enhancing Drugs

Five studies have investigated the prevalence and characteristics of PED use in powerlifting. In 1988, 15 out of 45 drug-tested powerlifters who were surveyed admitted anabolic steroid use [93], while two-thirds of international-level survey respondents reported anabolic steroid use in both 1995 [242] and 1999 [245]. Drug-testing samples from a range of sports were analysed in 2003, in which powerlifting and bodybuilding had the highest incidence of prohibited substances [255]. Finally, one study investigated the relationships between the knowledge of, attitudes towards, and use of anabolic steroids in athletes from different sports [256]. Of the 32 powerlifters in this sample, 93% reported steroid use. Unsurprisingly, those who used steroids were more knowledgeable about steroids and had more positive attitudes towards steroids.

#### Nutrition and Supplementation Summary and Applications

Studies have investigated the nutritional practices of powerlifters and the effects of ergogenic aids including caffeine [238, 251, 253], creatine [237, 238, 240, 241, 251, 254], and ammonia inhalants [239]. Rapid weight loss strategies (e.g., water loading, fluid restriction, and gradual dieting) have been of particular interest [30, 81, 243, 249, 250]. Notably, studies including para powerlifters have only examined the impacts of creatine [240] and ibuprofen [235, 236, 246] on performance or recovery, justifying further research in this population. Nutrition and supplementation literature can improve athletes’ and coaches’ decision making when implementing dietary strategies, utilising legal supplements, and cutting weight for competitions. Additionally, the use of illegal PEDs in powerlifting has been investigated. While prevalent anabolic steroid use has previously been reported [93, 242, 245, 255, 256], the most recent article on this topic was published in 2003 [255]; thus, up-to-date investigation into the prevalence and determinants of PED use may be valuable for policymakers engaged in anti-doping efforts.

### Gaps in the Literature and Future Recommendations

Despite the substantial breadth of research investigating the applied sport science and medicine of powerlifting and para powerlifting, this review has identified several areas that warrant further investigation (Table 3). These recommendations for future research directions have been made based upon the current available literature, the advancement of scientific practices, and changes in powerlifting and para powerlifting training and performance since the earliest research into the sports. Our suggestions include, but are not limited to, greater inclusivity (i.e., inclusion of under-represented populations identified during this review), better understanding of the relationships between lifting techniques and injury, and investigation into the current prevalence of PEDs along with the systemic factors influencing their use.

**Table 3:** Recommendations for future powerlifting and para powerlifting research directions

Research area	Focus	Topics of interest	Clinical and practical relevance of this research and impact upon performance and policy
All	Under-represented populations	Females; youth; masters	Improve support for under-represented populations
Biomechanics	Analysis using gold-standard technology	Musculoskeletal modelling of forces and muscle contributions; comparison between powerlifters and other athletes or non-athletes; up-to-date bench press investigation	Support athletes and coaches in implementing more targeted training interventions and preventing injuries
Training	Longer interventions that emphasise higher quality outcome measures	Periodisation; autoregulation; advanced training methods; fatigue and recovery; optimal training dose; chronic training effects in para powerlifters	Enhance training interventions and exercise prescription
Competition	Physical demands of competitions	Forces applied to tissues; fatigue and recovery	Allow athletes to adequately prepare for specific competition demands
Physical qualities	Improved assessment of specific physical qualities	Musculoskeletal imaging; physical qualities associated with PED use; longitudinal monitoring; classification of para powerlifters; muscle characteristics of para powerlifters	Understand physical factors influencing performance and long-term implications of participation
Injury	Lifting techniques associated with injury	Shoulder, lower back, and knee injury prevention; high-quality biomechanical analysis; prospective injury investigation; para powerlifting techniques	Optimise lift execution to reduce the risk of injury
Psychology	Performance psychology	Effectiveness of different psychological techniques; broader factors influencing performance from a complex systems perspective; training adherence	Support athletes and coaches in implementing mental strategies to improve performance
Nutrition and supplementation	Ergogenic aids	Efficacy of different nutritional supplements; para powerlifting nutrition; up-to-date doping prevalence; environmental and behavioural determinants of PED use	Support athletes and coaches in reducing risk and implementing effective supplementation

*PED* performance-enhancing drug

A number of recommendations could also be implemented to enhance the quality of future powerlifting and para powerlifting research. While every effort has been made to review the state of the current literature, due to a lack of consistent reporting standards, nonstandard terminology, and varying interpretations of results, further analysis was not possible. Thus, to support future research, guidelines for the reporting of participant characteristics in powerlifting and para powerlifting research are proposed in Table 4. These guidelines include the clear reporting of demographic characteristics, level of competition, powerlifting division, and athlete classification. While these factors may not be perceived as integral for the aims of an individual paper, they may help improve the interpretation of findings and will ensure more consistent reporting standards. Additionally, several general recommendations for future research can be made. First, authors should ensure they use standard powerlifting terminology (e.g., ‘powerlifting’ instead of ‘power lifting’ [33] and ‘deadlift’ instead of ‘ground lift’ [104]). While there are inconsistent definitions between the many powerlifting federations globally, widely accepted nomenclature such as the names of the three lifts and the typical competitive divisions should be understood by researchers and used appropriately; if uncertain, authors should refer to the rulebook of the federation they deem most relevant. Second, authors should uphold rigorous scientific practices (e.g., pre-registration with the Open Science Framework [257] and clear reporting of statistical power). Finally, studies analysing data from public repositories of powerlifting results should clearly report the characteristics of these data, including their origin, the data handling practices employed, and how the integrity of the data has been managed when reporting inconsistencies may have occurred.

**Table 4:** Framework for the reporting of participant characteristics in powerlifting and para powerlifting research

Characteristic	Examples	Existing framework or reference	Rationale and impact on research quality
General characteristics	Age; sex; height; body mass	The rulebook of the federation in which the participant competes (e.g., IPF Technical Rules Book [7]) for specific sex classifications for transgender or non-binary athletes	Many of the included studies did not specify general participant characteristics, including sex; clear reporting would allow readers to evaluate the study’s relevance to different populations
Level of competition	Recreationally active; national level; international level	Participant classification framework by McKay et al. (2021) [258]	Many of the included studies did not specify the level of competition of participants or misused terms like ‘elite’; clear reporting would inform readers of the calibre of athlete to which the findings may apply
Powerlifting division	Raw; wraps; equipped (single-ply or multi-ply)	The rulebook of the federation in which the participant competes	Many of the included studies did not specify participants’ competitive division; clear reporting would allow readers to evaluate the study’s relevance to different competitors
Weight class	59 kg; 93 kg; 120 kg +	The rulebook of the federation in which the participant competes	Many of the included studies did not specify participants’ weight class; while studies should also report the exact body mass of participants, specification of their self-reported competitive weight class would provide more powerlifting-specific insights, especially for athletes who employ rapid weight loss strategies to compete in lighter weight classes
Age category	Sub-junior; junior; open; masters (I, II, etc.)	The rulebook of the federation in which the participant competes	Many of the included studies did not specify participants’ age category; while studies should also report the exact age of participants, clear reporting of the competitive age category would provide more powerlifting-specific insights and allow readers to evaluate the study’s relevance to individuals within different age groups
Testing status	Tested (under WADA or another testing body); untested	The rulebook of the federation in which the participant competes	Many of the included studies did not specify whether participants competed in tested competitions; clear reporting would provide greater context of potential PED use
Para classification	Impairment type (e.g., limb deficiency or short stature); origin of impairment (e.g., acquired or congenital)	The rulebook or classification rules of the federation in which the participant competes (e.g., WPPO Classification Rules and Regulations [15])	It has been suggested that para powerlifting’s current classification systems may be insufficient [155]; in the absence of updated classifications, studies should specify participants’ voluntarily self-reported and de-identified impairment details where possible to allow readers to evaluate the study’s relevance to different para-athletes

*IPF* International Powerlifting Federation, *PED* performance-enhancing drug, *WADA* World Anti-Doping Agency, *WPPO* World Para Powerlifting

## Conclusion

This scoping review identified 218 studies investigating the applied sport science and medicine of powerlifting or para powerlifting, with an exponential increase in literature observed in recent decades and more than half of the included studies published in 2020 or later. Much of this research originated from the USA, while the majority of para powerlifting studies originated from Brazil. Seven primary areas of research were identified, with physical qualities and competition leading as the most studied topics. Furthermore, it is apparent that the areas of nutrition and supplementation, injury, and psychology are relatively under-researched and could therefore benefit from increased attention. Certain populations are also under-represented, including females, who were solely investigated in only 3% of the included studies.

Suggestions for future research directions have been presented based on the gaps in the existing powerlifting and para powerlifting literature. While these concepts and their potential impact are subjective, these recommendations may provide useful starting points for future researchers wishing to expand on the body of evidence surrounding the sports. Additionally, a number of general recommendations around the reporting of participant characteristics, consistent terminology, and scientific rigour have been provided. Future powerlifting and para powerlifting studies should follow these recommendations to enhance their quality and consistency, allowing for improved interpretation by athletes, coaches, policymakers, and other researchers.

## Supplementary Information

Below is the link to the electronic supplementary material.

Supplementary file1 (DOCX 47 KB)

Supplementary file2 (DOCX 49 KB)

Supplementary file3 (DOCX 51 KB)

Supplementary file4 (DOCX 57 KB)

Supplementary file5 (DOCX 35 KB)

Supplementary file6 (DOCX 28 KB)

Supplementary file7 (DOCX 40 KB)

## Declarations

### Availability of Data and Materials

All data and material reported in this review are from peer-reviewed publications.
