# Supplementary material for: The Applied Sport Science and Medicine of Powerlifting and Para Powerlifting: A Systematic Scoping Review with Recommendations for Future Research
Source: Sports Med. 2025 Sep 9;55(11):2849–77. doi: 10.1007/s40279-025-02305-3 (PMC12559058; doi:10.1007/s40279-025-02305-3)
Supplement: Supplementary file 1 — Supplementary file1 (DOCX 47 KB) [file 40279_2025_2305_MOESM1_ESM.docx]

**Supplementary Table S1.** Characteristics, outcomes measures, and key findings of ‘biomechanics’ studies (n = 36)

| **Study** | **Cohort and sample size (n); age; body mass (where specified)** | **Competitive characteristics: para status; division; weight class; age category (where specified)** | **Study aim(s)** | **Outcome measures** | **Key findings** |
| --- | --- | --- | --- | --- | --- |
| Evaluation of strength and muscle activation indicators in sticking point region of national-level Paralympic powerlifting athletes (Aidar et al., 2021) | 12 Paralympic powerlifters; 26.56 ± 5.55 yrs; 77.89 ± 24.6 kg | Para | To evaluate the strength indicators and the sticking point region in different distances of the bar to the chest in elite Powerlifting Paralympic athletes | Maximum dynamic velocity and dynamic time; maximum isometric strength, rate of force development rate, and time to maximum isometric force; surface EMG | The maximum isometric force, rate of force development, time, velocity, and dynamic time had lower values, especially in the initial and intermediate phases in the sticking region |
| Force-velocity relationship in Paralympic powerlifting: two or multiple-point methods to determine a maximum repetition (Aidar et al., 2022a) | 15 elite male Paralympic powerlifters; 27.7 ± 5.7 yrs; 74.0 ± 19.5 kg | Para | To evaluate the precision of the multi-point method using proximal loads compared to the four-point method and the two-point method using distant loads in the bench press | Measurement of minimum velocity limit, load at zero velocity, and force-velocity; 1RM prediction | The multiple-point methods and the two-point method showed a good ability to predict bench press 1RM |
| Does the level of training interfere with the sustainability of static and dynamic strength in Paralympic powerlifting athletes? (Aidar et al., 2022b) | 11 national level (29.25 ± 4.50 yrs; 80.86 ± 15.36 kg) and 12 regional level (26.13 ± 7.22 yrs; 82.80 ± 31.73 kg) Paralympic powerlifters | Para | To analyse mechanical, dynamic, and static indicators of powerlifting performance at different intensities | Mean propulsive velocity; maximum velocity; power; 1RM; maximum isometric force; time to maximum isometric force; rate of force development; fatigue index | Regional athletes performed better in mean propulsive velocity and maximum velocity, but national athletes produced more power |
| Paralympic powerlifting as a sustainable way to improve strength in athletes with spinal cord injury and other disabilities (Aidar et al., 2022c) | 10 male Paralympic powerlifters with spinal cord injury (30.00 ± 4.27 yrs; 79.90 ± 18.91 kg), 10 with other disabilities (28.30 ± 4.92 yrs; 78.80 ± 18.98 kg) | Para | To evaluate mechanical strength indicators at different intensities in attached and non-attached conditions in athletes with spinal cord injury compared to other disabilities, in relation to powerlifting performance | Maximum isometric force, time to maximum isometric force, rate of force development, impulse, variability, fatigue index, mean propulsive velocity, maximum velocity, power | There were no differences between spinal cord injury and other disabilities in dynamic and isometric strength indicators, however there were differences between tethered and untethered conditions in athletes with spinal cord injury |
| Electromyographic and kinematic evaluation of bench press exercise: A case report study on athletes with different impairments and expertise (Bellitto et al., 2023) | An unimpaired athlete and three Paralympic athletes; 22 yrs, 20 yrs, 29 yrs, 40 yrs | Para and non-para | To define and test a setup and a protocol to quantitatively assess the execution of bench press exercise in athletes with different abilities | Muscle activity and kinematic data from the upper body | The instrumented evaluation proposed can assess the bench press exercise |
| Effect of supportive equipment on force, velocity, and power in the squat (Blatnik et al., 2012) | 8 elite or professional level male powerlifters; 25 ± 2.2 years; 106.85 ± 30.4 kg | Non-para | To examine the effect of squatting with and without a squat suit on various kinetic and kinematic parameters | Concentric peak force, concentric peak velocity, concentric peak power, eccentric peak force, eccentric peak velocity, eccentric peak power | Squats with the squat suit elicited higher velocity and power during the concentric portion of the exercise; the squat suit allowed individuals to maintain higher power outputs at lower intensities, and maintain higher velocity outputs at all intensities |
| Kinematics and kinetics of the dead lift in adolescent power lifters (Brown & Abani, 1985) | 10 skilled and 11 unskilled teenage male powerlifters | Non-para; all weight classes; teenage category | To document kinematic and kinetic characteristics of the deadlift performed by teenage powerlifters and to determine relationships among these characteristics | Body segment orientations; vertical bar accelerations; vertical joint reaction forces; segmental angular accelerations; horizontal moment arms of the bar to selected joints; intersegmental resultant moments | More upright posture at lift-off in the skilled group; maximal vertical bar acceleration and trunk angular acceleration tended to occur near lift-off in skilled lifters; the unskilled subjects demonstrated greater variability and magnitude in linear and angular acceleration parameters; maximum vertical force was at the ankle; maximum torque was at the hip; magnitude (not technique) determined the hip moment and ankle, knee, and hip vertical forces |
| Lumbar posterior ligament involvement during extremely heavy lifts estimated from fluoroscopic measurements (Cholewicki & McGill, 1992) | 4 competitive powerlifters; 31-34 yrs, 79.4-104.3 kg | Non-para | To use video fluoroscopy to obtain quantitative measures of vertebral rotation and translation during actual lifts in an attempt to evaluate ligament length and their contribution in resisting flexion moment | Lumbar range of motion; ligament length change; disc deformations | Ligaments did not strain sufficiently to contribute substantial resistance to the trunk flexion moment, relegating this responsibility to the musculature |
| Lumbar spine loads during the lifting of extremely heavy weights (Cholewicki et al., 1991) | 44 male (30.7 ± 5.5 yrs, 86.3 ± 17.4 kg) and 13 female (26.3 ± 3.5 yrs, 60.5 ± 7.4 kg) Canadian Powerlifting Championships competitors | Non-para; all weight classes | To document the extensor moment and the loads placed upon the lumbar spine during the lifting of extremely heavy weights performed by skilled male and female competitive powerlifters, to examine the relationship between lifting style and spinal load, and the relationship between low back moment and performance level | Lifting style; reaction moments at the knee, hip, and L4/L5 joints; compressive and shearing forces on L4/L5 | There is large variability in the pattern of loading joints among national class powerlifters; the sumo deadlift resulted in a reduction in joint moments and forces compared to conventional |
| Electromyographical activity of the pectoralis, triceps, and deltoideus during the sub-phases of bench press in Paralympic powerlifters (da Silva et al., 2022) | 6 male (26.5 ± 8.0 yrs) and 4 female (39.8 ± 11.2 yrs) national level Paralympic powerlifters | Para | To analyse the electromyographic and kinematic variables affecting the subphases of the bench press movement in Paralympic powerlifting | Barbell velocity; EMG signal | The triceps brachii presented high values of the frequency domain variables, especially in the concentric phase of the bench press movement, suggesting a firing rate higher than the other muscles studied |
| Does the grip width affect the bench press performance of Paralympic powerlifters? (dos Santos et al., 2020) | 12 national level Paralympic powerlifters; 25.40 ± 3.30 yrs; 70.30 ± 12.15 kg | Para | To evaluate the muscular strength and electromyographic response with different grip widths during the bench press exercise in Paralympic powerlifting athletes | Maximal dynamic and isometric strength; mean propulsive velocity; EMG analysis | The 1.5x biacromial distance grip width tended to show greater force generation and mean propulsive velocity, and this grip showed faster contractile responses |
| A biomechanical analysis of the sticking region in the bench press (Elliott et al., 1989) | 10 elite male powerlifters | Non-para; raw; 75-125+ kg; open | To identify the mechanisms during the bench press responsible for the previously reported sticking region phenomenon | Barbell kinematics and kinetics; minimum acceleration and velocity; peak acceleration and velocity; maximum displacement; EMG activity | The sticking region did not appear to be caused by an increase in the moment arm of the weight about the shoulder or elbow joints of by a minimisation of muscular activity during this region |
| A three-dimensional biomechanical analysis of the squat during varying stance widths (Escamilla et al., 2001) | 39 national level male powerlifters; 45.7 ± 5.2 yrs; 91.0 ± 25.2 kg | Non-para; masters (40+ yrs) | To compare joint and segment angles and ankle, knee, and hip moments and moment arms between 2D and 3D analyses while performing the squat with varying stance widths | Barbell load; stance width; hand width; barbell velocity; joint angles and velocities; segment angles; joint moments and moment arms | Ankle plantarflexor net muscle moments were generated during the narrow stance, ankle dorsiflexor net muscle moments were produced during the medium stance and wide stance, and knee and hip moments were greater during the wide stance compared with the narrow stance |
| A three-dimensional biomechanical analysis of sumo and conventional style deadlifts (Escamilla et al., 2000) | 24 national level male powerlifters: 12 conventional (46.4 ± 6.1 yrs, 76.8 ± 22.7 kg), 12 sumo (47.4 ± 7.3 yrs, 71.6 ± 10.8 kg) | Non-para; masters (40+ yrs) | To compare joint and segment angles and ankle, knee, and hip moments and moment arms using three-dimensional (3-D) analyses during sumo and conventional deadlifts | Barbell load; barbell velocity; joint angles and velocity; segment angles; joint moments and moment arms | Hip extensor, knee extensor, and ankle dorsiflexor moments were generated in sumo, whereas hip extensor, knee extensor, knee flexor, and ankle plantarflexor moments were generated in conventional. Ankle and knee moments and moment arms were significantly different, but hip moments and moment arms were not |
| The high-bar and low-bar back-squats: a biomechanical analysis (Glassbrook et al., 2019) | 6 international level male powerlifters (21-33 yrs; 87.1 ± 8.0 kg), 6 national level male weightlifters (22-30 yrs; 83.1 ± 13.0 kg), 6 recreationally trained male athletes (23-33 yrs; 87.9 ± 15.3 kg) | Non-para | To compare and contrast the differences in joint angles and vertical ground reaction force of the high-bar back squat and low-bar back squat, up to and including maximal efforts | Joint angles; vertical ground reaction forces | The low-bar back squat places more emphasis on the hip musculature and allows greater loads to be lifted; the high-bar back squat places more emphasis on the knee musculature and replicates upright torso movements like the snatch and clean |
| The effects of grip width on sticking region in bench press (Gomo & Van Den Tillaar, 2016) | 12 national level male powerlifters; 27.73 ± 8.82 yrs; 91.85 ± 15.41 kg | Non-para | To examine the occurrence of the sticking region by examining how three different grip widths affect the sticking region in bench press performance of experienced powerlifters | Joint angles and moment arms; timing; barbell position and velocity | All participants showed a clear sticking region with all three grip widths, but this sticking region was not found to occur at the same joint angles in all three grip widths |
| The loads on the lumbar spine during extreme weight lifting (Granhed et al., 1987) | 8 international level Swedish male powerlifters; 23-40 yrs; 59-93 kg | Non-para | To calculate the load on the lumbar spine during deadlifts, and to find to what extent training for such extreme weight lifting would affect the incorporation of bone mineral and thus the strength of the lumbar vertebrae | L3 load; L3 bone mineral content | The loads on L3 in the lifters ranged between 18.8 and 36.4 kN; the bone mineral content values were extremely high and closely correlated to the amount of weight lifted during training |
| Are sEMG, velocity and power influenced by athletes’ fixation in Paralympic powerlifting (Guerra et al., 2022) | 15 elite Paralympic powerlifters; 22.27 ± 10.30 yrs; 78.50 ± 21.67 kg | Para | To analyse the variations in surface EMG, maximum velocity, mean propulsive velocity, and muscle power of Paralympic powerlifters during the bench press with various loads performed with legs tied and untied on the bench | Surface EMG; maximum velocity; mean propulsive velocity; power | The tied condition seems to favour muscle activation, surface EMG, and velocity over the untied condition |
| Kinematic analysis of the powerlifting style squat and the conventional deadlift during competition: is there a cross-over effect between lifts? (Hales et al., 2009) | 25 regional level male powerlifters; 28 ± 8 yrs; 93.63 ± 30.9 kg | Non-para | To compare and contrast biomechanical parameters between the conventional style deadlift and the back squat performed by 25 lifters competing in a regional powerlifting championship | Hip, knee, and ankle kinematics; segment lengths; vertical barbell velocity | Significant vertical barbell velocity differences exist between the squat and the deadlift; differences were found for angular position of the hip, knee, and ankle; the back squat is a synergistic movement, while the deadlift is a sequential movement |
| RPE and velocity relationships for the back squat, bench press, and deadlift in powerlifters (Helms et al., 2017) | 12 male and 3 female powerlifters; 28.4 ± 8.5 yrs; 82.1 ± 18.9 kg | Non-para; tested | To assess both reps in reserve-based RPE and average velocity in competitive male and female powerlifters, on all 3 powerlifts, in competition order and to determine any relationships between RPE and average velocity | Squat, bench press, and deadlift 1RM; RPE; average concentric velocity; anthropometric measurements | The average concentric velocity showed strong and very strong inverse relationships with RPE and percentage 1RM on each lift, respectively; RPE may be a useful tool for prescribing intensity for squat, bench press, and deadlift in powerlifters, in addition to traditional methods such as percentage of 1RM |
| Kinematic factors influencing performance and injury risk in the bench press exercise (Madsen & McLaughlin, 1984) | 17 novice and 19 national or international level male powerlifters | Non-para | To investigate the kinematics and kinetics of the barbell during the bench press | Barbell kinematics and kinetics | The kinematic factors that have been identified as potentially important in the bench press are the possible existence of a ‘sticking point’; the position of the bar path relative to the shoulders; the sequence of bar movements used in raising the bar; the degree of control maintained in lowering the bar; and the role of grip spacing |
| Biomechanical analysis of the deadlift (McGuigan & Wilson, 1996) | 29 male New Zealand Powerlifting Federation powerlifters | Non-para | To investigate whether there is a preferred deadlifting technique among elite competitive powerlifters and where the sticking region occurs | Segment and joint angles and range of motion; vertical barbell velocity; bar path | Conventional lifters had a significantly greater range of knee extension prior to liftoff; sumo lifters maintained a more upright posture at liftoff; the distance the barbell had to travel was significantly reduced in sumo; sumo reduced resistance lever arm distances |
| A kinematic model of performance in the parallel squat by champion powerlifters (McLaughlin et al., 1977) | 24 national level powerlifters | Non-para | To investigate the kinematic factors that describe the movements in the parallel squat with the intent to formulate a model of performance and to utilise this model to contrast performances of high and less-skilled subjects | Horizontal and vertical velocities and accelerations for bar, hip, and knee; absolute angular velocities and accelerations for trunk, thigh, and shank | Although there was some variability in most kinematic parameters, vertical bar velocity was very similar mong parameters |
| Kinetics of the parallel squat (McLaughlin et al., 1978) | 12 national level powerlifters | Non-para | To determine the joint forces and muscular torques during performance of the parallel squat for some of the best national and world-class powerlifters | Muscle torques; joint forces | Results indicated that high-skilled subjects minimized trunk torques by maintaining a more erect trunk position and also demonstrated more extensor-dominant thigh torques |
| Bench press techniques of elite heavyweight powerlifters (McLaughlin & Madsen, 1984) | 9 elite male powerlifters | Non-para; 242 lbs (n = 3), 275 lbs (n = 3), super heavyweight (n = 3) weight classes | To examine the kinematic and kinetic factors influencing performance and injury risk for a group of elite heavyweight powerlifters | Force exerted on bar; torque produced at shoulder; rate of work done on bar; horizontal distance from shoulder to bar at various instants; angle of displacement with horizontal angle of bar from instant to instant; bar accelerations; time | Power outputs were greater for heavy subjects; torques about the shoulder can be expected to be larger for bigger athletes in the bench press |
| Effects of bench press eccentric tempo modification on dynamic and static strength performance in powerlifting athletes with disabilities (Méndez-DelCanto et al., 2025) | 16 national level male Paralympic powerlifters; 29.1 ± 7.8 yrs; 79.1 ± 24.2 kg | Para | To investigate the effects of three different eccentric tempo conditions (normative, slower, or faster than normative eccentric tempo) over dynamic strength performance during bench press, and static strength performance after the same bench press set in experienced para powerlifting athletes | Maximum velocity; mean propulsive velocity; power; maximal isometric force; rate of force development; impulse; isometric force variability; maximal isometric force over one second | The normally accustomed execution proved to be more effective than both slower and faster rhythms in enhancing neuromuscular outcomes in experienced para powerlifting athletes; slower and faster cadences appear to reduce dynamic and static strength indicators, suggesting that experienced lifters may have an optimized eccentric execution |
| Force production and muscle activation during partial vs. full range of motion in Paralympic powerlifting (Mendonça et al., 2021) | 12 national level male Paralympic powerlifters; 28.60 ± 7.60 yrs; 71.80 ± 17.90 kg | Para | To compare the fatigue index, the maximum isometric force, the time to maximum isometric force, the rate of strength development, the muscle thickness and the activation of the muscles involved in partial vs. full range of motion in Paralympic powerlifting | Fatigue index; maximum isometric force; time to maximum isometric force; rate of strength development; muscle thickness; EMG analysis | Post-exercise fatigue indicators were higher with full range of motion (as given by a greater fatigue index and by a greater loss of maximal isometric force); training with partial ROM enables handling of higher workloads with lower loss of muscle function. |
| A biomechanical analysis of the squat between competitive collegiate, competitive high school, and novice powerlifters (Miletello et al., 2009) | 9 competitive collegiate powerlifters (19.78 ± 1.39 yrs; 77.42 ± 21.85 kg), 9 competitive high school powerlifters (17.22 ± 0.67 yrs; 66.50 ± 11.81 kg), 11 novice powerlifters (20.09 ± 1.38 yrs; 74.71 ± 11.61 kg) | Non-para | To measure and analyse kinematic differences between competitive collegiate powerlifters, competitive high school powerlifters, and novice powerlifters during a squat performed at maximum weight for 1 repetition to determine the effect of skill level on performance | Descent, ascent, and total lift times; knee angle magnitude; knee angular velocity; knee angular acceleration | The high school and novice groups accumulated several significant differences in normalised time to peak during the ascending phase; the major finding between the 3 groups was in the rate of acceleration upward after coming out of the hole |
| Muscle force dynamics across increasing squat intensity conditions in elite powerlifters (Pürzel et al., 2025) | 16 male and 13 female national level Austrian powerlifters; 26.1 ± 5.4 yrs; 83.1 ± 19.4 kg | Non-para | To determine how increasing intensities from 70% to 90% of 1RM impacts muscle forces during the squat in elite powerlifters | Lower limb muscle forces | Muscle forces significantly changed with increased intensity, particularly in the gluteus maximus and semitendinosus, which showed the greatest relative increase in muscle force; the vastii muscles exhibited the highest absolute muscle forces; the hamstrings, calf, and vastii muscle forces barely increased during the deepest and most challenging region of the squat (the sticking region) with increasing intensity |
| Factors underlying bench press performance in elite competitive powerlifters (Reya et al., 2021) | 13 national or international level male powerlifters; 26 ± 9 yrs; 93.8 ± 9.9 kg | Non-para; tested | To investigate a number of possible determinants of the 1RM bench press performance collectively among competitive powerlifters who are the athletes that likely exhibit the highest performance in this exercise | Anthropometric measurements; muscle cross-sectional area; bench press 1RM; EMG analysis; kinematic and kinetic variables; maximal isokinetic strength | The highest degree of association was shown for structural followed by neuromuscular factors, whereas technical factors did not correlate with 1RM bench press performance; lean body mass, the agonist cross-sectional area, brachial index, and strength of the elbow and shoulder flexors were the best predictors of performance |
| Performance differences between the arched and flat bench press in beginner and experienced Paralympic powerlifters (Ribeiro Neto et al., 2022) | 20 beginner and 23 experienced Paralympic powerlifters; 35.0 ± 8.2 yrs; 53.2-80.4 kg | Para | To verify the differences of the total load, the trajectory of the barbell in the sagittal plane, and the mean velocity of the barbell between the arched and flat techniques of the bench press in beginner and experienced Paralympic powerlifters | Bench press 1RM; sagittal plane barbell trajectory; barbell mean velocity | The total load, trajectory of the barbell in the sagittal plane, and mean velocity of the barbell were not significantly different between the arched and flat techniques for experienced and beginner powerlifters during both the eccentric and concentric phase of the movement |
| Variability analysis of muscle activation symmetry to identify indicators of individual motor strategy: a case series on elite Paralympic powerlifters (Rum et al., 2023) | 5 international level Paralympic powerlifters; 30.0 ± 5.1 yrs; 74.1 ± 17.1 kg | Para | To explore muscle activation symmetry and its intra- and inter-individual variability to determine the muscles mostly related to individual motor strategies in elite Paralympic powerlifters | Muscle activation symmetry | The highest variability and asymmetry in abdominal muscle activation among athletes emphasize the importance of personalized training approaches for targeting these muscles due to their role in individualizing motor strategies |
| An analysis of the barbell motion depending on its weight in disabled powerlifting (Seidel & Zurowska, 2014) | 29 disabled athletes from the National Powerlifting Team; 23.9 ± 6.1 yrs | Para | To assess the symmetry of flexion and extension movements in upper limbs in disabled powerlifters during bench press with different barbell loads | Angular velocities, movement onset, time point of maximum angular velocity, degree of movement asymmetry | Elbow joint movement onset and maximal angular velocity did not change significantly with the increased barbell load, suggesting most movements were performed symmetrically |
| Static and dynamic strength indicators in Paralympic power-lifters with and without spinal cord injury (Teles et al., 2021) | 9 male Paralympic powerlifters with spinal cord injuries (30.57 ± 4.20 kg; 81.29 ± 21.68 kg), 10 with other deficiencies (25.67 ± 4.52 yrs; 73.89 ± 17.56 kg) | Para | To analyse mechanical, dynamic and static indicators of strength, at different intensities, on performance in athletes with spinal cord injury and other deficiencies of Paralympic powerlifting | Maximum isometric force; time to maximum isometric force; rate of force development; impulse; variability and fatigue index; mean propulsive velocity; maximum velocity; power; EMG analysis | There were differences in relation to mean propulsive velocity and maximum velocity; there were no differences in the static force indicators; the triceps tend to be more activated in the spinal cord injury group and the deltoids in the other deficiencies group |
| The effects of barbell placement on kinematics and muscle activation around the sticking region in squats (van den Tillaar et al., 2020) | 10 competitive male powerlifters; 26.1 ± 1.2 yrs; 90.2 ± 18.3 kg | Non-para | To investigate whether bar position (low bar and high bar) affects the back squat sticking region within the sticking region performed with the same absolute external load on kinematics, muscle activity, and joint angles | Barbell and joint kinematics; EMG analysis | When depth and stance width were matched, the low bar technique was associated with lower erector spinae and quadriceps activity than the high bar technique; thus, when the goal is to maximally activate knee extensors and the external load is matched, high bar placement would appear preferable |
| Bar path and force profile characteristics for maximal and submaximal loads in the bench press (Wilson et al., 1989) | 10 state, national, or international level male powerlifters | Non-para; 75-125+ kg weight classes | To analyse the bar movement characteristics of 10 elite powerlifters while bench pressing a maximum load and a submaximal load in a simulated competition using high-speed cinematography | Barbell kinematics and kinetics | The movement pattern adopted during the performance of an 81% maximum load was not specific to that which was utilized during the maximal load; based upon the concepts of specificity of training and testing, the use of the popular 1RM test to quantify strength changes derived from submaximal training appear invalid |

**References**

Aidar, F. J., Brito, C. J., de Matos, D. G., de Oliveira, L. A. S., de Souza, R. F., de Almeida-Neto, P. F., de Araújo Tinoco Cabral, B. G., Neiva, H. P., Neto, F. R., Reis, V. M., Marinho, D. A., Marques, M. C., Clemente, F. M., & Nobari, H. (2022a). Force-velocity relationship in Paralympic powerlifting: Two or multiple-point methods to determine a maximum repetition. *BMC Sports Science, Medicine and Rehabilitation*, *14*(1), 1-15. <https://doi.org/10.1186/s13102-022-00552-9>

Aidar, F. J., Cataldi, S., Badicu, G., Silva, A. F., Clemente, F. M., Bonavolontà, V., Greco, G., Getirana-Mota, M., & Fischetti, F. (2022b). Does the level of training interfere with the sustainability of static and dynamic strength in Paralympic powerlifting athletes? *Sustainability*, *14*(9). <https://doi.org/10.3390/su14095049>

Aidar, F. J., Cataldi, S., Badicu, G., Silva, A. F., Clemente, F. M., Latino, F., Greco, G., & Fischetti, F. (2022c). Paralympic powerlifting as a sustainable way to improve strength in athletes with spinal cord injury and other disabilities. *Sustainability*, *14*(4). <https://doi.org/10.3390/su14042017>

Aidar, F. J., Clemente, F. M., de Matos, D. G., Marçal, A. C., de Souza, R. F., Moreira, O. C., de Almeida-Neto, P. F., Vilaça-Alves, J., Garrido, N. D., dos Santos, J. L., Jeffreys, I., Neto, F. R., Reis, V. M., de Araújo Tinoco Cabral, B. G., Rosemann, T., & Knechtle, B. (2021). Evaluation of strength and muscle activation indicators in sticking point region of national-level Paralympic powerlifting athletes. *Journal of Functional Morphology and Kinesiology*, *6*(2), 1-12. <https://doi.org/10.3390/jfmk6020043>

Bellitto, A., Marchesi, G., Comini, M., Massone, A., Casadio, M., & De Luca, A. (2023). Electromyographic and kinematic evaluation of bench press exercise: A case report study on athletes with different impairments and expertise. *Sport Sciences for Health*, *19*(2), 723-732. <https://doi.org/10.1007/s11332-022-00949-6>

Blatnik, J. A., Skinner, J. W., & McBride, J. M. (2012). Effect of supportive equipment on force, velocity, and power in the squat. *Journal of Strength and Conditioning Research*, *26*(12), 3204-3208. <https://doi.org/10.1519/JSC.0b013e3182736641>

Brown, E. W., & Abani, K. (1985). Kinematics and kinetics of the dead lift in adolescent power lifters. *Medicine and Science in Sports and Exercise*, *17*(5), 554-563.

Cholewicki, J., & McGill, S. M. (1992). Lumbar posterior ligament involvement during extremely heavy lifts estimated from fluoroscopic measurements. *Journal of Biomechanics*, *25*(1), 17-28. <https://doi.org/10.1016/0021-9290(92)90242-s>

Cholewicki, J., McGill, S. M., & Norman, R. W. (1991). Lumbar spine loads during the lifting of extremely heavy weights. *Medicine and Science in Sports and Exercise*, *23*(10), 1179-1186.

da Silva, B. G., Miziara, I. M., Furtado, D. A., dos Santos, S. S., Fidale, T. M., & Pereira, A. A. (2022). Electromyographical activity of the pectoralis, triceps, and deltoideus during the sub-phases of bench press in paralympic powerlifters. *Sports Engineering*, *25*(1), 1-10. <https://doi.org/10.1007/s12283-022-00377-2>

dos Santos, M. D. M., Aidar, F. J., de Souza, R. F., dos Santos, J. L., da Silva de Mello, A., Neiva, H. P., Marinho, D. A., & Marques, M. C. (2020). Does the grip width affect the bench press performance of Paralympic powerlifters? *International Journal of Sports Physiology and Performance*, *15*(9), 1252-1259. <https://doi.org/10.1123/ijspp.2019-0784>

Elliott, B. C., Wilson, G. J., & Kerr, G. K. (1989). A biomechanical analysis of the sticking region in the bench press. *Medicine and Science in Sports and Exercise*, *21*(4), 450-462.

Escamilla, R. F., Fleisig, G. S., Lowry, T. M., Barrentine, S. W., & Andrews, J. R. (2001). A three-dimensional biomechanical analysis of the squat during varying stance widths. *Medicine and Science in Sports and Exercise*, *33*(6), 984-998. <https://doi.org/10.1097/00005768-200106000-00019>

Escamilla, R. F., Francisco, A. C., Fleisig, G. S., Barrentine, S. W., Welch, C. M., Kayes, A. V., Speer, K. P., & Andrews, J. R. (2000). A three-dimensional biomechanical analysis of sumo and conventional style deadlifts. *Medicine and Science in Sports and Exercise*, *32*(7), 1265-1275. <https://doi.org/10.1097/00005768-200007000-00013>

Glassbrook, D. J., Brown, S. R., Helms, E. R., Duncan, S., & Storey, A. G. (2019). The high-bar and low-bar back-squats: A biomechanical analysis. *Journal of Strength and Conditioning Research*, *33 Suppl 1*, S1-S18. <https://doi.org/10.1519/JSC.0000000000001836>

Gomo, O., & Van Den Tillaar, R. (2016). The effects of grip width on sticking region in bench press. *Journal of Sports Sciences*, *34*(3), 232-238. <https://doi.org/10.1080/02640414.2015.1046395>

Granhed, H., Jonson, R., & Hansson, T. (1987). The loads on the lumbar spine during extreme weight lifting. *Spine*, *12*(2), 146-149. <https://doi.org/10.1097/00007632-198703000-00010>

Guerra, I., Aidar, F. J., Greco, G., de Almeida-Neto, P. F., De Candia, M., de Araújo Tinoco Cabral, B. G., Poli, L., Filho, M. M., Carvutto, R., Silva, A. F., Clemente, F. M., Badicu, G., Cataldi, S., & Fischetti, F. (2022). Are sEMG, velocity and power influenced by athletes' fixation in Paralympic powerlifting? *International Journal of Environmental Research and Public Health*, *19*(7). <https://doi.org/10.3390/ijerph19074127>

Hales, M. E., Johnson, B. F., & Johnson, J. T. (2009). Kinematic analysis of the powerlifting style squat and the conventional deadlift during competition: Is there a cross-over effect between lifts? *Journal of Strength and Conditioning Research*, *23*(9), 2574-2580. <https://doi.org/10.1519/JSC.0b013e3181bc1d2a>

Helms, E. R., Storey, A., Cross, M. R., Brown, S. R., Lenetsky, S., Ramsay, H., Dillen, C., & Zourdos, M. C. (2017). RPE and velocity relationships for the back squat, bench press, and deadlift in powerlifters. *Journal of Strength and Conditioning Research*, *31*(2), 292-297. <https://doi.org/10.1519/JSC.0000000000001517>

Madsen, N., & McLaughlin, T. (1984). Kinematic factors influencing performance and injury risk in the bench press exercise. *Medicine and Science in Sports and Exercise*, *16*(4), 376-381.

McGuigan, M. R. M., & Wilson, B. D. (1996). Biomechanical analysis of the deadlift. *Journal of Strength and Conditioning Research*, *10*(4), 250-255.

McLaughlin, T. M., Dillman, C. J., & Lardner, T. J. (1977). A kinematic model of performance in the parallel squat by champion powerlifters. *Medicine and Science in Sports and Exercise*, *9*(2), 128-133. <https://doi.org/10.1249/00005768-197709020-00011>

McLaughlin, T. M., Lardner, T. J., & Dillman, C. J. (1978). Kinetics of the parallel squat. *Research Quarterly. American Alliance for Health, Physical Education and Recreation*, *49*(2), 175-189. <https://doi.org/10.1080/10671315.1978.10615522>

McLaughlin, T. M., & Madsen, N. H. (1984). Bench press techniques of elite heavyweight powerlifters. *Strength and Conditioning Journal*, *6*(4), 44.

Méndez-DelCanto, R., Aidar, F. J., Banja, T., dos Santos, J. L., da Silva-Grigoletto, M. E., Marçal, A. C., Aedo-Muñoz, E., Brito, C. J., & Nikolaidis, P. T. (2025). Effects of bench press eccentric tempo modification on dynamic and static strength performance in powerlifting athletes with disabilities. *Sport Sciences for Health*, 1-9. <https://doi.org/10.1007/s11332-025-01417-7>

Mendonça, T. P., Aidar, F. J., Matos, D. G., Souza, R. F., Marçal, A. C., Almeida-Neto, P. F., Cabral, B. G., Garrido, N. D., Neiva, H. P., Marinho, D. A., Marques, M. C., & Reis, V. M. (2021). Force production and muscle activation during partial vs. full range of motion in Paralympic powerlifting. *PloS one*, *16*(10), e0257810. <https://doi.org/10.1371/journal.pone.0257810>

Miletello, W. M., Beam, J. R., & Cooper, Z. C. (2009). A biomechanical analysis of the squat between competitive collegiate, competitive high school, and novice powerlifters. *Journal of Strength and Conditioning Research*, *23*(5), 1611-1617. <https://doi.org/10.1519/JSC.0b013e3181a3c6ef>

Pürzel, A., Kaufmann, P., Koller, W., Pöhlmann, L., Baca, A., & Kainz, H. (2025). Muscle force dynamics across increasing squat intensity conditions in elite powerlifters. *Scandinavian Journal of Medicine and Science in Sports*, *35*(5), 1-12. <https://doi.org/10.1111/sms.70058>

Reya, M., Škarabot, J., Cvetičanin, B., & Šarabon, N. (2021). Factors underlying bench press performance in elite competitive powerlifters. *Journal of Strength and Conditioning Research*, *35*(8), 2179-2186. <https://doi.org/10.1519/jsc.0000000000003097>

Ribeiro Neto, F., Dorneles, J. R., Luna, R. M., Spina, M. A., Gonçalves, C. W., & Gomes Costa, R. R. (2022). Performance differences between the arched and flat bench press in beginner and experienced Paralympic powerlifters. *Journal of Strength and Conditioning Research*, *36*(7), 1936-1943. <https://doi.org/10.1519/JSC.0000000000003736>

Rum, L., Romagnoli, R., Lazich, A., Sciarra, T., Balletti, N., Piacentini, M. F., Boraschi, A., & Bergamini, E. (2023). Variability analysis of muscle activation symmetry to identify indicators of individual motor strategy: A case series on elite Paralympic powerlifters. *Frontiers in Sports and Active Living*, *5*, 1290964. <https://doi.org/10.3389/fspor.2023.1290964>

Seidel, W., & Zurowska, A. (2014). An analysis of the barbell motion depending on its weight in disabled powerlifting. *Baltic Journal of Health and Physical Activity*, *6*(3), 193-198. <https://doi.org/10.2478/bjha-2014-0017>

Teles, L. J. L., Aidar, F. J., Matos, D. G., Marçal, A. C., Almeida-Neto, P. F., Neves, E. B., Moreira, O. C., Ribeiro Neto, F., Garrido, N. D., Vilaça-Alves, J., Díaz-de-Durana, A. L., Clemente, F. M., Jeffreys, I., Cabral, B., & Reis, V. M. (2021). Static and dynamic strength indicators in Paralympic power-lifters with and without spinal cord injury. *International Journal of Environmental Research and Public Health*, *18*(11). <https://doi.org/10.3390/ijerph18115907>

van den Tillaar, R., Knutli, T. R., & Larsen, S. (2020). The effects of barbell placement on kinematics and muscle activation around the sticking region in squats. *Frontiers in Sports and Active Living*, *2*, 604177. <https://doi.org/10.3389/fspor.2020.604177>

Wilson, G. J., Elliott, B. C., & Kerr, G. K. (1989). Bar path and force profile characteristics for maximal and submaximal loads in the bench press. *Journal of Applied Biomechanics*, *5*(4), 390-402. <https://doi.org/10.1123/ijsb.5.4.390>
