# Supplementary material for: The Applied Sport Science and Medicine of Powerlifting and Para Powerlifting: A Systematic Scoping Review with Recommendations for Future Research
Source: Sports Med. 2025 Sep 9;55(11):2849–77. doi: 10.1007/s40279-025-02305-3 (PMC12559058; doi:10.1007/s40279-025-02305-3)
Supplement: Supplementary file 2 — Supplementary file2 (DOCX 49 KB) [file 40279_2025_2305_MOESM2_ESM.docx]

**Supplementary Table S2.** Characteristics, outcomes measures, and key findings of ‘training’ studies (n = 38)

| **Study** | **Cohort and sample size (n); age; body mass (where specified)** | **Competitive characteristics: para status; division; weight class; age category (where specified)** | **Study aim(s)** | **Outcome measures** | **Key findings** |
| --- | --- | --- | --- | --- | --- |
| Evaluation of training with elastic bands on strength and fatigue indicators in Paralympic powerlifting (Aidar et al., 2021) | 12 Paralympic powerlifters; 28.6 ± 7.6 yrs; 71.8 ± 17.9 kg | Para | To assess the effect of a training session using elastic bands vs with fixed resistance, through evaluation of static and dynamic indicators of force and fatigue | 1RM; maximum isometric force; peak torque; rate of force development; fatigue index; time to maximum isometric force | Training using elastic bends tends to not decrease 1RM, peak torque, maximum isometric force, or rate of force development, but it does increase fatigue and time to maximum isometric force |
| Enhancing post-training muscle recovery and strength in Paralympic powerlifting athletes with cold-water immersion, a cross-sectional study (Aidar et al., 2025) | 11 national or international level male Paralympic powerlifters; 27.1 ± 4.7 yrs; 84.6 ± 18.5 kg | Para | To assess the effects of different post-training recovery methods on muscle damage in Paralympic powerlifting athletes | Blood biochemical markers; maximum isometric force; time to maximum isometric force; rate of force development | Cold-water immersion significantly improved the post-resistance training muscle damage and strength in Paralympic powerlifting athletes |
| Effect of 16 weeks of periodized resistance training on strength gains of powerlifting athletes (Allegretti João et al., 2014) | 9 elite male powerlifters; 34.5 ± 5.0 yrs; 94.4 ± 16.7 kg | Non-para; 67 kg (n = 1), 70-100 kg (n = 6), 100+ kg (n = 2) | To assess the effects of a 16-week linear periodisation resistance training macrocycle on muscular strength gains in elite powerlifting athletes | Squat, bench press, and deadlift 1RM | After 16 weeks of training, there was a significant 33.5% increase in the squat, 30.3% in the bench press, and 76.9% in the deadlift compared to pre-training values |
| Reduced volume ‘daily max’ training compared to higher volume periodized training in powerlifters preparing for competition – a pilot study (Androulakis-Korakakis et al., 2018) | 10 beginner-intermediate male powerlifters; 27 ± 6 yrs; 90.5 ± 16.7 kg | Non-para | To compare the implementation of a ‘daily max’ style training approach to that of a traditional periodised training approach in powerlifting athletes preparing for competition over a 10-week training cycle | Squat, bench press, and deadlift 1RM | Competition performance decreased in 3 out of 5 participants in the ‘daily max’ group, thus this style of training may only be appropriate for certain athletes when preparing for competition |
| The minimum effective training dose required for 1RM strength in powerlifters (Androulakis-Korakakis et al., 2021) | 5 studies: 28 elite powerlifters and experienced coaches, 137 powerlifters and coaches of all levels, 16 intermediate-advanced powerlifters, 9 beginner-intermediate powerlifters, 58 intermediate-advanced powerlifters | Non-para; raw and equipped | 5 studies: to understand the concept of minimum effective training dose, to understand what powerlifters and coaches regard as meaningful strength increases, to explore the effect of two different training protocols on 1RM, to understand the minimum effective does practices of powerlifters | Subjective measures of training practices and perceptions; 1RM | Powerlifters can utilise the minimum effective training dose during periods of limited time availability, deloads, and competition preparation, and doing so may be a useful strategy to manage fatigue and injury risk |
| The effect of resistance training set configuration on strength and muscular performance adaptations in male powerlifters (Arazi et al., 2021) | 24 college-aged powerlifters: cluster set group (23.6 ± 1.3 yrs; 78.6 ± 4.5 kg), traditional set group (23.3 ± 0.9 yrs; 79.2 ± 4.1 kg), control group (23.8 ± 1.3 yrs; 77.2 ± 4.9 kg) | Non-para | To examine the effects of training with cluster sets or traditional sets during an 8-week training period in college-aged male powerlifters | Medicine ball throw: lower body peak power; 1RM back squat, bench press, deadlift; body composition | The cluster set and traditional set groups indicated similar changes in 1RM bench press, back squat, and deadlift following the 8 weeks training intervention |
| Type 1 muscle fiber hypertrophy after blood flow–restricted training in powerlifters (Bjørnsen et al., 2019) | 16 male and 3 female elite Norwegian powerlifters: blood flow restriction group (24 ± 3 yrs; 89 ± 14 kg), conventional group (26 ± 8 yrs; 102 ± 18 kg) | Non-para | To investigate the effects of blood flow-restricted resistance exercise on myofiber areas, number of myonuclei and satellite cells, muscle size and strength in powerlifters | Strength; muscle cross sectional area and thickness; satellite cells and myonuclei; fiber type | Two 1-week blocks with high-frequency low-load blood flow restriction implemented during 6 weeks of periodised strength training induced a significant increase in muscle size and myonuclear addition in elite powerlifters; preferential hypertrophy and myonuclear addition of type I fibres appears to explain most of the overall muscle growth |
| Two days versus four days of training cessation following a step-taper in powerlifters (Burke et al., 2023) | 8 competitive and 4 non-competitive powerlifters (10 males, 2 females); 22.3 ± 2.1 yrs, 92.1 ± 20.4 kg) | Non-para | To complete an evidence-based peaking protocol that included a 1-week planned overreach prior to a 1-week step-taper followed by 2 days or 4 days of training cessation, comparing differences in performance, recovery, and body composition | Squat, bench press, and deadlift 1RM; DOTS score; body composition; subjective recovery and strength state | There were significant increases in bench press, total, and DOTS score after 2 days of cessation; after 4 days of cessation, significant increases were only observed in deadlift along with significant decreases in bench press; there were no significant changes in any other variable |
| Effect of strength training on psychophysiological aspects in Paralympic powerlifting athletes: a pilot study (Chaves Da Silva et al., 2022) | 7 male Paralympic powerlifters; 41.0 ± 10.1 yrs; 84.7 ± 21.1 kg | Para | To evaluate the maximum dynamic strength, mood levels, stress, and recovery over a period of strength training in Paralympic powerlifting athletes | 1RM; subjective measures of stress, recovery and mood; resting heart rate | Increased maximum dynamic strength, positive and negative changes in the dimensions of stress/recovery and mood, and no alteration of resting heart rate during a period of progressive loading |
| Relationship between online coaching characteristics, lifter satisfaction, and performance in powerlifters (Clarke et al., 2025) | 59 male (27.1 ± 8.9 yrs) and 54 female (35.5 ± 10.1 yrs) powerlifters | Non-para | To understand the length, mode, and frequency of online powerlifting coaching delivery and their relationship with lifter satisfaction and powerlifting performance | Subjective measures of online coaching characteristics, lifter satisfaction, and self-reported performance | There was a positive relationship between longer coach-lifter relationships and greater increases in total; higher coach education was associated with increases in total |
| Body composition and maximal strength of powerlifters: a descriptive quantitative and longitudinal study (Ferland et al., 2023) | 21 male (29.7 ± 10.1 yrs; 92.2 ± 15.9 kg) and 13 female (27.4 ± 5.7 yrs; 71.0 ± 19.0 kg) powerlifters | Non-para; classic; -59 kg = 1, -74 kg = 3, -83 kg = 3, -93 kg = 7, -105 kg = 5, -120 kg = 1, 120 kg+ = 1 for men, -52 kg = 3, -57 kg = 1, -63 kg 2, -72 kg = 4, -84 kg+ = 3 for women; juniors = 7, open = 25, masters = 2 | To present relationships between maximal strength and body composition and to conduct yearly follow-ups to present the chronic effects of maximal strength training on body composition | DEXA body composition; squat, bench press, and deadlift 1RM; subjective measures of training practices | Maximal strength can be used to predict bone mineral content and bone mineral density, while lean body mass can be used to predict maximal strength; consistent powerlifting practice can increase bone mineral density in adults |
| Analysis of velocity, power and skin temperature in Paralympic powerlifting athletes with fixed and variable resistance (Filho et al., 2024) | 14 national level Paralympic powerlifters; 28.00 ± 5.64 yrs; 82.00 ± 23.93 kg | Para | To analyse the use of variable resistance training and the traditional method on speed, power, and temperature in a training session | Propulsive mean velocity; power; skin temperatures | Variable resistance training promoted greater muscle fatigue when compared to traditional training |
| Comparison of neuromuscular fatigue in powerlifting Paralympics in different training methods (Getirana-Mota et al., 2024) | 11 national level male Paralympic powerlifters; 31.54 ± 9.72 yrs; 73.63 ± 17.55 kg | Para | To evaluate the neuromuscular fatigue indicators in Paralympic powerlifters using two different training methods: the inertial method and the eccentric/concentric training method incorporating supramaximal loads in the eccentric phase of the adapted bench press | Mean propulsive velocity | There was no significant neuromuscular fatigue for the inertial method, and no significant decline in mean propulsive velocity during the intervention; for the eccentric/concentric training method, there was a significant reduction in mean propulsive velocity before and after training; a difference between the methods after the intervention was identified |
| Effects of a 6-week bench press program using the freak bar in a sample of collegiate club powerlifters (Ghigiarelli et al., 2018) | 7 male and 3 female collegiate powerlifters; freak bar group = 21.0 ± 2.7 yrs; traditional barbell group = 21.8 ± 1.3 yrs | Non-para; raw | To evaluate the effects of a 6-week training program using the freak bar for the bench press exercise in a sample of collegiate powerlifters | Bench press 1RM, maximum isometric force, and peak impulse | The freak bar can be an alternative for improving maximum strength and peak force, but is not significantly better than traditional barbell training when performing the 3-position pause bench press |
| Influence of compressive gear on powerlifting performance: role of blood flow restriction training (Godawa et al., 2012) | 14 male and 4 female collegiate powerlifters; 18-26 yrs | Non-para | To compare differences in performance gains between powerlifters who are both training and competing in equipment, thought to restrict blood flow, with those training and competing without such gear | Training volume; 1RM squat, bench press, deadlift, and total; body composition; blood pressure; heart rate; vascular stiffness | The magnitude of improvements in the squat and total were greater in the compressive gear group, suggesting an ergogenic potential of training with powerlifting gear |
| Impact of low-load high-volume initial sets vs. traditional high-load low-volume bench press protocols on functional and structural adaptations in powerlifters (González-Alcázar et al., 2025) | 26 male powerlifters; low-load high-volume group = 23.2 ± 2.7 yrs, 81.8 ± 9.2 kg; high-load low-volume group = 23.9 ± 3.7 yrs, 80.8 ± 12.7 kg | Non-para | To evaluate the effects of a 12-week low-load high-volume bench press protocol compared to a traditional high-load low-volume approach on key performance and structural outcomes in well-trained powerlifters | Bench press 1RM; mean velocity; arm and chest circumferences | Low-load high-volume resistance training represents a practical and effective alternative to traditional high-load low-volume protocols, yielding comparable functional and structural adaptations in powerlifters |
| Tapering practices of Croatian open-class powerlifting champions (Grgic & Mikulic, 2017) | 6 male (29.9 ± 3.8 yrs; 86.3 ± 16.8 kg) and 4 female (28.3 ± 2.2 yrs; 64.2 ± 9.4 kg) national level Croatian Powerlifting Federation powerlifters | Non-para; raw; open | To investigate tapering practices and types of tapering employed by Croation national powerlifting champions | Subjective measures of training practices | Decreased training volume during the taper; training intensity was maintained or increased; training frequency was reduced or maintained |
| Effect of training phase on physical and physiological parameters of male powerlifters (Hackett et al., 2020) | 9 male competitive powerlifters; median 36.0 yrs; median 87.6 kg | Non-para; tested | To examine the impact of the preparatory and competition phases of training on physical and physiological parameters in male natural powerlifters | Muscular strength, power, and endurance; flexibility; body composition; pulse wave velocity and blood pressure; subjective measures of diet and training practices; blood and urine analysis | Despite modifications in training and dietary practices, it appears that muscle performance, body composition, and health status remain relatively stable between training phases in male natural powerlifters |
| Self-rated accuracy of rating of perceived exertion-based load prescription in powerlifters (Helms et al., 2017) | 9 male (81.9 ± 12.5 kg) and 3 female (59.0 ± 5.8 kg) New Zealander International Powerlifting federation powerlifters | Non-para; tested | To assess nationally qualified male and female powerlifters’ ability to accurately select loads resulting in a target reps-in-reserve-based rating of perceived exertion for a single set in the squat, bench press, and deadlift on hypertrophy-, power-, and strength-type sessions over 3 weeks | Rating of perceived exertion accuracy | Powerlifters can select loads to reach a self-rated target rating of perceived exertion with precision after a familiarisation session |
| Rating of perceived exertion as a method of volume autoregulation within a periodized program (Helms et al., 2018) | 9 male and 3 female competitive powerlifters; 26.3 ± 6.8 yrs; 76.2 ± 15.0 kg | Non-para; tested | To observe the impact of implementing RPE stops on training volume in powerlifters performing the back squat, bench press, and deadlift in 3 weekly sessions: 1 hypertrophy-, 1 strength-, and 1 power-type training day for 3 weeks | Squat, bench press, and deadlift training load | Volume can be effectively autoregulated using RPE stops as a method to dictate number of sets performed |
| Powerlifting training methods for women aged 35-40 years: Organization and structure of the training process for achieving maximum sports results (Hordiienko et al., 2024) | 12 female powerlifters; 35-40 yrs | Non-para | To conduct a comprehensive analysis of the factors influencing powerlifting on the physical and psychological health of women aged 35-40 years, and to develop an author’s method of training to achieve high sports results and prevent injuries during powerlifting | Maximal squat, bench press, and deadlift load and repetitions | The training methodology, developed based on alternating mesocycles of different duration and intensity, is an effective means of preparing female athletes for competitions and achieving maximum sports results |
| Effects of augmented eccentric load bench press training on one repetition maximum performance and electromyographic activity in trained powerlifters (Montalvo et al., 2021) | 5 male and 3 female competitive powerlifters; 26.25 ± 4.13 yrs; 79.09 ± 6.38 kg | Non-para | To determine the effects of a 4-week augmented eccentric load intervention on bench press 1RM, surface EMG activity, and various bar kinetics and kinematics in competitive powerlifters | Bench press 1RM; EMG activity; bar kinetics and kinematics | Incorporating augmented eccentric load bench press training into a 4-week training cycle may be a novel strategy to improve 1RM performance in competitive powerlifters in a short period |
| Tapering practices of New Zealand’s elite raw powerlifters (Pritchard et al., 2016) | 8 male and 3 female elite New Zealand powerlifters; 28.4 ± 7.0 yrs; 91.0 ± 27.4 kg | Non-para | To gain insight into the current tapering strategies of elite powerlifters, why such strategies are used, how these strategies were developed, and how tapering might differ for each lift | Subjective measures of tapering practices | Total training volume peaked 5.2 ± 1.7 weeks from competition while average training intensity (of 1RM) peaked 1.9 ± 0.8 weeks from competition; during tapering, volume was reduced by 58.9 ± 8.4% while intensity was maintained (or slightly reduced) and the final weight training session was performed 3.7 ± 1.6 days out from competition |
| Are strength indicators and skin temperature affected by the type of warm-up in Paralympic powerlifting athletes? (Resende et al., 2021) | 15 national level male Paralympic powerlifters; 28.47 ± 5.79 yrs; 81.75 ± 17.33 kg | Para | To evaluate the effect of different types of warm-ups on the strength and skin temperature of Paralympic powerlifting athletes | Skin temperature at 8 regions of interest; 1RM; mean propulsive velocity; impulse; variability; peak torque | The types of warm-ups studied do not seem to interfere with the performance of Paralympic powerlifting athletes; however, the thermal images showed that traditional warm-up best meets the objectives expected for this preparation phase |
| The influence of warm-up on body temperature and strength performance in Brazilian national-level Paralympic powerlifting athletes (Resende et al., 2020) | 12 male national level Paralympic powerlifters; 24.14 ± 6.21 yrs; 81.67 ± 17.36 kg | Para | To analyse the effect of different types of warm-ups on the physical performance of elite Paralympic powerlifters | 1RM; Maximum isometric force; rate of force development; maximum velocity; fatigue index; tympanic temperature | A significant difference was observed for maximum isometric force with no warm-up compared to traditional and stretching warm-ups; there were no significant differences in rate of force development, fatigue index, or time; there were no significant differences in 1RM or maximum velocity; significant temperature differences were found for the traditional warm-up in relation to the ‘before’ and ‘after’ conditions, and between no warm-up and stretching warm-up in the ‘after’ condition |
| Acute effect of whole-body vibration on power, one-repetition maximum, and muscle activation in power lifters (Rønnestad et al., 2012) | 12 Norwegian national level male powerlifters; 24 ± 5 yrs; 110 ± 24 kg | Non-para; tested | To investigate the effect of whole-body vibration (50 Hz) on peak power and EMG activity in the squat jump with an external load of 65 and 100 kg and on 1RM and EMG activity in the parallel squat and compare them with no-vibration conditions in powerlifters | Squat jump peak power; Parallel squat 1RM; EMG activity | The application of whole-body vibration (50 Hz) acutely increases peak power output during the squat jump in powerlifters; this increase in power was accompanied by an increased EMG activity in the quadriceps muscles; however, in the 1RM parallel squat, there was no difference between whole-body vibration (50 Hz) and no-vibration conditions |
| Assessing the effects of different training programs for physical preparation and sports performance in power lifters with visual impairments of high qualification (Roztorhui et al., 2021) | 16 visually impaired national level powerlifters; 34.50 ± 4.31 yrs | Non-para | To experimentally substantiate the effectiveness of the impact of various training programs on physical fitness and sports performance of powerlifters with visual impairments of high qualification | Maximum push-ups; seated medicine ball throw; sit and reach test; one-leg balancing test; squat, bench press, and deadlift 1RM | The performance of the experimental group (lower volume and intensity) is much higher than among the powerlifters of the control group (higher volume and intensity) |
| Physiological and biochemical evaluation of different types of recovery in national level Paralympic powerlifting (Santos et al., 2021) | 12 national level male Paralympic powerlifters; 25.4 ± 3.3 yrs; 70.3 ± 12.2 kg | Para | To evaluate the effects of different post-training recovery methods on mechanical, biochemical, and pain scales in Paralympic powerlifting athletes | Muscle thickness; blood biochemical indicators; maximum isometric force; subjective measures of pain threshold | Maximal force decreased compared to the pretest value; cold water immersion and dry needling increased Interleukin 2 levels from 24 to 48 h more than that from 2 h to 24 h; after dry needling, muscle thickness did not increase significantly in any of the muscles, and after 2 h, muscle thickness decreased significantly again in the major pectoralis muscle; after cold water immersion, pain pressure stabilised after 15 min and increased significantly again after 2 h for acromial pectoralis |
| Contemporary training practices of Norwegian powerlifters (Shaw et al., 2022) | 66 male and 51 female regional or higher level powerlifters (32.3 ± 11.1 yrs) | Non-para | To extend on the existing powerlifting training literature to provide a broader descriptive overview of the powerlifting training practices of a successful country | Subjective measures of training practices | Norwegian powerlifters’ training differs from practices previously identified in the literature, with a higher prevalence of elastic resistance, particularly for those competing internationally, and a decreased use of strength training exercises at all levels; Norwegian powerlifters train frequently (5 or more times per week) and with submaximal loads |
| Stretching practices of International Powerlifting Federation unequipped powerlifters (Spence et al., 2022) | 240 male and 79 female regional, national, or international level International Powerlifting Federation powerlifters | Non-para; raw tested | To determine how prevalent stretching is among powerlifters and investigate the ways stretching is performed and used | Subjective measures of training practices, injury history, and stretching practices | 52.4% of subjects reported stretching; of those, 84.4% performed static stretches, and 90.4% performed dynamic stretches; stretching was performed before resistance training by 77.8%, after resistance training by 43.7%, and 53.9% stretched independent of resistance training |
| Effect of Paralympic powerlifting training on sleep and its relationship with training load (Stieler et al., 2022) | 9 male and 2 female national level Paralympic powerlifters | Para | To compare the sleep parameters of Paralympic powerlifters with physical disabilities between days with and without training, and to analyse the relationship between training load and sleep on the same day and the relationship between the previous night’s sleep and the training load on the following day | Sleep parameters; subjective measures of chronotype and sleep characteristics; training load and RPE | Athletes show morning and indifferent chronotype and low daytime sleepiness; on training days, sleep onset latency was lower, whereas total sleep time and sleep efficiency were higher compared to non-training days; the total sleep time of the night before the training days correlated positively with the RPE of the following day, and the training volume correlated negatively with the sleep efficiency of the same day |
| Contemporary training practices in elite British powerlifters: survey results from an international competition (Swinton et al., 2009) | 32 international level male powerlifters | Non-para | To investigate the contemporary training practices of elite powerlifters | Subjective measures of training practices | The majority of powerlifters train with the intention to explosively lift maximal and submaximal loads; 39% of the lifters regularly used elastic bands and 57% incorporated chains in their training; 69% of the subjects reported using Olympic lifts or their derivatives |
| Skeletal muscle adaptations and performance outcomes following a step and exponential taper in strength athletes (Travis et al., 2021b) | 14 male and 2 female powerlifters; 24.2 ± 4.0 yrs; 89.8 ± 21.4 kg | Non-para | To compare performance outcomes and skeletal muscle adaptations following a 6-week peaking program using a step or exponential taper in strength athletes | Squat jump performance; isometric squat performance; squat, bench press, and deadlift 1RM; body composition; vastus lateralis cross-sectional area; immunohistochemical analysis; muscle fibre analysis; muscle messenger RNA and mRNA analyses | An overreach followed by a step taper appears to produce a myocellular environment that enhances skeletal muscle adaptations, whereas an exponential taper may favour neuromuscular performance |
| Characterizing the tapering practices of United States and Canadian raw powerlifters (Travis et al., 2021a) | 225 male and 139 female North American powerlifters | Non-para; raw; all weight classes; sub-junior, open, and masters categories | To characterise the tapering practices of powerlifters from the United States and Canada to determine whether tapering practices differed by sex, competition level, and competition lift | Subjective measures of training and tapering practices | The highest training volume most frequently took place 5–8 weeks before competition, whereas the highest training intensity was completed 2 weeks before competition; A step taper was primarily used over 7–10 days while decreasing the training volume by 41–50% with varied intensity; the final heavy back squat and deadlift sessions were completed 7-10 days before competition, whereas the final heavy bench press session was completed <7 days before competition; final heavy lifts were completed at 90.0-92.5% 1RM but reduced to 75-80% 1RM for back squat and bench press and 70-75% for deadlift during the final training session of each lift |
| Endocrine response to high intensity barbell squats performed with constant movement tempo and variable training volume (Wilk et al., 2018) | 28 national or international level powerlifters; 27.8 ± 2.9 yrs; 85.3 ± 3.3 kg | Non-para | To determine the effect of variable volume in squat exercise with constant intensity and constant tempo on post-exercise concentrations of selected anabolic and catabolic hormones and growth factors; to determine the range of training volume which elicited the greatest anabolic hormone secretion while limiting the increase in serum cortisol | Blood concentrations of testosterone, growth hormone, insulin-like growth factor-1, and serum cortisol | In terms of endocrine response, the optimal volume of high intensity strength exercise is 6 sets, and intentionally high volume or low volume are not effective stimuli for endocrine responses of trained individuals; 6 sets of squats seem to drive hormonal responses of growth hormone, serum cortisol, and insulin-like growth factor-1, which may play a significant role in stimulating muscle growth and tissue regeneration |
| Bench press load-velocity profiles and strength after overload and taper microcycles in male powerlifters (Williams et al., 2020) | 12 male powerlifters; 24.2 ± 5.6 yrs; 98.7 ± 14.2 kg | Non-para | To determine the effect of an overload microcycle and taper on bench press mean concentric velocity and 1RM and to determine if the load-velocity relationship can accurately predict free-weight bench press 1RM | Bench press mean concentric velocity and 1RM | The average mean concentric velocity decreased after overload compared with baseline but increased after taper; 1RM increased from overload to taper; predicted 1RM was consistently higher than measured 1RM, but very large to near perfect correlations were observed between predicted 1RM and 1RM; the load-velocity relationship established from submaximal sets did not accurately predict 1RM, but mean concentric velocity was affected by changes in weekly training loads |
| Efficacy of daily one-repetition maximum training in well-trained powerlifters and weightlifters: a case series (Zourdos et al., 2016a) | 2 United States of America Powerlifting powerlifters (28 and 34 yrs; 80.5 and 108.8 kg) and 1 United States of America Weightlifting weightlifter (19 yrs; 64.1 kg) | Non-para; raw | To examine the efficacy of daily 1RM and volume training on the back squat, followed by volume sets of the back squat, for producing 1RM strength enhancement in well-trained competitive powerlifters/weightlifters over 37 consecutive days, and to investigate the effects of this training strategy on muscle hypertrophy | 1RM and rating of perceived exertion; average velocity; Wilks coefficient; body composition; perceived recovery status; muscle thickness; subjective measures of training history | Daily 1RM training effectively produced robust changes in maximal strength in competitive strength athletes in a relatively short training period |
| Modified daily undulating periodization model produces greater performance than a traditional configuration in powerlifters (Zourdos et al., 2016b) | 18 collegiate male powerlifters; 21.1 ± 1.9 yrs; 82.6 ± 11.4 kg | Non-para; raw tested | To compare 2 daily undulating periodisation models on 1RM strength in the squat, bench press, deadlift, total volume lifted, and temporal hormone response | Squat, bench press, and deadlift 1RM; Wilks coefficient; total volume; total repetitions; blood biochemical analysis; subjective measures of physical activity experience; subjective measures of dietary practices; body fat percentage | A hypertrophy-specific, power-specific, strength-specific weekly training order configuration of daily undulating periodisation has enhanced performance benefits compared with a hypertrophy-strength-power weekly training order |

**References**

Aidar, F. J., Clemente, F. M., de Lima, L. F., de Matos, D. G., Ferreira, A. R. P., Marçal, A. C., Moreira, O. C., Bulhões-Correia, A., de Almeida-Neto, P. F., Díaz-de-Durana, A. L., Neves, E. B., Cabral, B. G. A. T., Reis, V. M., Garrido, N. D., Nikolaidis, P. T., & Knechtle, B. (2021). Evaluation of training with elastic bands on strength and fatigue indicators in Paralympic powerlifting. *Sports*, *9*(10), 142-142. <https://doi.org/10.3390/sports9100142>

Aidar, F. J., Santos, W. Y. H. D., Machado, S. D. C., Nunes-Silva, A., Vieira, É. L. M., Valenzuela Pérez, D. I., Aedo-Muñoz, E., Brito, C. J., & Nikolaidis, P. T. (2025). Enhancing post-training muscle recovery and strength in Paralympic powerlifting athletes with cold-water immersion, a cross-sectional study. *International Journal of Environmental Research and Public Health*, *22*(1), Article 122. <https://doi.org/10.3390/ijerph22010122>

Allegretti João, G., Lopes Evangelista, A., Gomes, J. H., Charro, M. A., Bocalini, D., Cardozo, D., Seixas da Silva, D. A. d. C., Simão, R., & Figueira Junior, A. (2014). Effect of 16 weeks of periodized resistance training on strength gains of powerlifting athletes. *Journal of Exercise Physiology Online*, *17*(3), 102-109.

Androulakis-Korakakis, P., Fisher, J. P., Steele, J., Kolokotronis, P., & Gentil, P. (2018). Reduced volume 'daily max' training compared to higher volume periodized training in powerlifters preparing for competition—a pilot study. *Sports*, *6*(3), 86. <https://doi.org/10.3390/sports6030086>

Androulakis-Korakakis, P., Michalopoulos, N., Fisher, J. P., Keogh, J., Loenneke, J. P., Helms, E., Wolf, M., Nuckols, G., & Steele, J. (2021). The minimum effective training dose required for 1RM strength in powerlifters. *Frontiers in Sports and Active Living*, *3*, 713655. <https://doi.org/10.3389/fspor.2021.713655>

Arazi, H., Khoshnoud, A., Asadi, A., & Tufano, J. J. (2021). The effect of resistance training set configuration on strength and muscular performance adaptations in male powerlifters. *Scientific Reports*, *11*(1), 7844. <https://doi.org/10.1038/s41598-021-87372-y>

Bjørnsen, T., Wernbom, M., Kirketeig, A., Paulsen, G., Samnøy, L. E., Bækken, L. V., Cameron-Smith, D., Berntsen, S., & Raastad, T. (2019). Type 1 muscle fiber hypertrophy after blood flow-restricted training in powerlifters. *Medicine and Science in Sports and Exercise*, *51*(2), 288-298. <https://doi.org/10.1249/MSS.0000000000001775>

Burke, B. I., Carroll, K. M., Travis, S. K., Stone, M. E., & Stone, M. H. (2023). Two days versus four days of training cessation following a step-taper in powerlifters. *Journal of Strength and Conditioning Research*, *37*(12), 625-632. <https://doi.org/10.1519/JSC.0000000000004564>

Chaves Da Silva, D., Matos Dos Santos, M. D., Aidar, F. J., Guilherme De Araújo Tinoco Cabral, B., Stieler, E., Alves Resende, R., Pereira De Andrade, A. G., Francisco De Almeida-Neto, P., Bulhões-Correia, A., Carvalho Guerreiro, R., Túlio De Mello, M., & Da Silva, A. (2022). Effect of strength training on psychophysiological aspects in Paralympic powerlifting athletes: A pilot study. *Human Movement*, *23*(3), 150-159. <https://doi.org/10.5114/hm.2022.111391>

Clarke, J. V., Spence, A.-J., Helms, E. R., & Cross, M. R. (2025). Relationship between online coaching characteristics, lifter satisfaction, and performance in powerlifters. *Journal of Strength and Conditioning Research*, *39*(6), 666-671. <https://doi.org/10.1519/JSC.0000000000005099>

Ferland, P.-M., Charron, J., Brisebois-Boies, M., Miron, F. S.-J., & Comtois, A. S. (2023). Body composition and maximal strength of powerlifters: A descriptive quantitative and longitudinal study. *International Journal of Exercise Science*, *16*(4), 828-845.

Filho, G. F. d. S., Aidar, F. J., Getirana-Mota, M., Brito, C. J., Aedo-Muñoz, E., de Almeida Paz, Â., de Souza Leite Júnior, J. A., Vieira, E. L. M., & Nikolaidis, P. T. (2024). Analysis of velocity, power and skin temperature in Paralympic powerlifting athletes with fixed and variable resistance. *Sports*, *12*(9), 250. <https://doi.org/10.3390/sports12090250>

Getirana-Mota, M., Aidar, F. J., Ribeiro Neto, F., Santos, T. P., Almeida-Neto, P. F. d., Cabral, B. G. d. A. T., Vieira-Souza, L. M., D Garrido, N., Reis, V. M., & Souza, R. F. (2024). Comparison of neuromuscular fatigue in powerlifting Paralympics in different training methods. *Sports Medicine International Open*, *8*, a22077922. <https://doi.org/10.1055/a-2207-7922>

Ghigiarelli, J. J., Pelton, L. M., Gonzalez, A. M., Fulop, A. M., Gee, J. Y., & Sell, K. M. (2018). Effects of a 6-week bench press program using the freak bar in a sample of collegiate club powerlifters. *Journal of Strength and Conditioning Research*, *32*(4), 938-949. <https://doi.org/10.1519/JSC.0000000000002430>

Godawa, T. M., Credeur, D. P., & Welsch, M. A. (2012). Influence of compressive gear on powerlifting performance: Role of blood flow restriction training. *Journal of Strength and Conditioning Research*, *26*(5), 1274-1280. <https://doi.org/10.1519/JSC.0b013e3182510643>

González-Alcázar, F. J., Jiménez-Martínez, P., Alix-Fages, C., Ruiz-Ariza, A., Casuso, R. A., Varela-Goicoechea, J., García-Ramos, A., & Jerez-Martínez, A. (2025). Impact of low-load high-volume initial sets vs. traditional high-load low-volume bench press protocols on functional and structural adaptations in powerlifters. *Applied Sciences*, *15*(4), Article 1974. <https://doi.org/10.3390/app15041974>

Grgic, J., & Mikulic, P. (2017). Tapering practices of Croatian open-class powerlifting champions. *Journal of Strength and Conditioning Research*, *31*(9), 2371-2378. <https://doi.org/10.1519/JSC.0000000000001699>

Hackett, D. A., Wilson, G. C., Mitchell, L., Haghighi, M. M., Clarke, J. L., Mavros, Y., O'Connor, H., Hagstrom, A. D., Slater, G. J., Keogh, J., & McLellan, C. (2020). Effect of training phase on physical and physiological parameters of male powerlifters. *Sports*, *8*(8), 106. <https://doi.org/10.3390/sports8080106>

Helms, E. R., Brown, S. R., Cross, M. R., Storey, A., Cronin, J., & Zourdos, M. C. (2017). Self-rated accuracy of rating of perceived exertion-based load prescription in powerlifters. *Journal of Strength and Conditioning Research*, *31*(10), 2938-2943. <https://doi.org/10.1519/JSC.0000000000002097>

Helms, E. R., Cross, M. R., Brown, S. R., Storey, A., Cronin, J., & Zourdos, M. C. (2018). Rating of perceived exertion as a method of volume autoregulation within a periodized program. *Journal of Strength and Conditioning Research*, *32*(6), 1627-1636. <https://doi.org/10.1519/JSC.0000000000002032>

Hordiienko, O. V., Zhamardiy, V. O., Horoshko, V. I., Danylchenko, S. I., & Novopysmennyi, A. M. (2024). Powerlifting training methods for women aged 35-40 years: Organization and structure of the training process for achieving maximum sports results. *Clinical and Preventive Medicine*, *2024*(8), 137-143. <https://doi.org/10.31612/2616-4868.8.2024.16>

Montalvo, S., Gruber, L. D., Gonzalez, M. P., Dietze-Hermosa, M. S., & Dorgo, S. (2021). Effects of augmented eccentric load bench press training on one repetition maximum performance and electromyographic activity in trained powerlifters. *Journal of Strength and Conditioning Research*, *35*(6), 1512-1519. <https://doi.org/10.1519/jsc.0000000000004030>

Pritchard, H. J., Tod, D. A., Barnes, M. J., Keogh, J. W., & McGuigan, M. R. (2016). Tapering practices of New Zealand's elite raw powerlifters. *Journal of Strength and Conditioning Research*, *30*(7), 1796-1804. <https://doi.org/10.1519/JSC.0000000000001292>

Resende, M. d. A., Aidar, F. J., Vasconcelos Resende, R. B., Reis, G. C., de Oliveira Barros, L., de Matos, D. G., Marçal, A. C., de Almeida-Neto, P. F., Díaz-de-Durana, A. L., Merino-Fernández, M., Vilaça-Alves, J., de Araújo Tinoco Cabral, B. G., Neves, E. B., Reis, V. M., Clemente, F. M., & Garrido, N. D. (2021). Are strength indicators and skin temperature affected by the type of warm-up in Paralympic powerlifting athletes? *Healthcare*, *9*(8). <https://doi.org/10.3390/healthcare9080923>

Resende, M. d. A., Vasconcelos Resende, R. B., Reis, G. C., Barros, L. d. O., Bezerra, M. R. S., Matos, D. G. d., Marçal, A. C., Almeida-Neto, P. F. d., Cabral, B. G. d. A. T., Neiva, H. P., Marinho, D. A., Marques, M. C., Reis, V. M., Garrido, N. D., & Aidar, F. J. (2020). The influence of warm-up on body temperature and strength performance in Brazilian national-level Paralympic powerlifting athletes. *Medicina*, *56*(10). <https://doi.org/10.3390/medicina56100538>

Rønnestad, B. R., Holden, G., Samnøy, L. E., & Paulsen, G. (2012). Acute effect of whole-body vibration on power, one-repetition maximum, and muscle activation in power lifters. *Journal of Strength and Conditioning Research*, *26*(2), 531-539. <https://doi.org/10.1519/JSC.0b013e318220d9bb>

Roztorhui, M., Perederiy, A., Khimenes, K., & Tovstonoh, O. (2021). Assessing the effects of different training programs for physical preparation and sports performance in power lifters with visual impairments of high qualification. *SportLogia*, *17*(1), 44-56. <https://doi.org/10.5550/sgia.211701.en.rpkt>

Santos, W., Aidar, F. J., Matos, D. G., Van den Tillaar, R., Marçal, A. C., Lobo, L. F., Marcucci-Barbosa, L. S., Machado, S. D. C., Almeida-Neto, P. F., Garrido, N. D., Reis, V. M., Vieira É, L. M., Cabral, B., Vilaça-Alves, J., Nunes-Silva, A., & Júnior, W. (2021). Physiological and biochemical evaluation of different types of recovery in national level Paralympic powerlifting. *International Journal of Environmental Research and Public Health*, *18*(10). <https://doi.org/10.3390/ijerph18105155>

Shaw, M. P., Andersen, V., Sæterbakken, A. H., Paulsen, G., Samnøy, L. E., & Solstad, T. E. J. (2022). Contemporary training practices of Norwegian powerlifters. *Journal of Strength and Conditioning Research*, *36*(9), 2544-2551. <https://doi.org/10.1519/JSC.0000000000003584>

Spence, A.-J., Helms, E. R., & McGuigan, M. R. (2022). Stretching practices of International Powerlifting Federation unequipped powerlifters. *Journal of Strength and Conditioning Research*, *36*(12), 3456-3461. <https://doi.org/10.1519/jsc.0000000000003800>

Stieler, E., Silva, F. R., Grade, I., Andrade, H. A., Guerreiro, R. C., Resende, R., Andrade, A. G., Gonçalves, D. A., Santos, M. D. M., Silva, A., & Mello, M. T. (2022). Effect of Paralympic powerlifting training on sleep and its relationship with training load. *Motriz. Revista de Educacao Fisica*, *28*. <https://doi.org/10.1590/s1980-657420220016321>

Swinton, P. A., Lloyd, R., Agouris, I., & Stewart, A. (2009). Contemporary training practices in elite British powerlifters: Survey results from an international competition. *Journal of Strength and Conditioning Research*, *23*(2), 380-384. <https://doi.org/10.1519/JSC.0b013e31819424bd>

Travis, S. K., Pritchard, H. J., Mujika, I., Gentles, J. A., Stone, M. H., & Bazyler, C. D. (2021a). Characterizing the tapering practices of United States and Canadian raw powerlifters. *Journal of Strength and Conditioning Research*, *35*, S26-S35. <https://doi.org/10.1519/JSC.0000000000004177>

Travis, S. K., Zwetsloot, K. A., Mujika, I., Stone, M. H., & Bazyler, C. D. (2021b). Skeletal muscle adaptations and performance outcomes following a step and exponential taper in strength athletes. *Frontiers in Physiology*, *12*, 735932. <https://doi.org/10.3389/fphys.2021.735932>

Wilk, M., Petr, M., Krzysztofik, M., Zajac, A., & Stastny, P. (2018). Endocrine response to high intensity barbell squats performed with constant movement tempo and variable training volume. *Neuroendocrinology Letters*, *39*(4), 342-348.

Williams, T. D., Esco, M. R., Fedewa, M. V., & Bishop, P. A. (2020). Bench press load-velocity profiles and strength after overload and taper microcyles in male powerlifters. *Journal of Strength and Conditioning Research*, *34*(12), 3338-3345. <https://doi.org/10.1519/jsc.0000000000003835>

Zourdos, M. C., Dolan, C., Quiles, J. M., Klemp, A., Blanco, R., Whitehurst, M., Jo, E., & Loenneke, J. P. (2016a). Efficacy of daily one-repetition maximum training in well-trained powerlifters and weightlifters: A case series. *Nutrición Hospitalaria*, *33*(2), 437-443.

Zourdos, M. C., Jo, E., Khamoui, A. V., Lee, S.-R., Park, B.-S., Ormsbee, M. J., Panton, L. B., Contreras, R. J., & Kim, J.-S. (2016b). Modified daily undulating periodization model produces greater performance than a traditional configuration in powerlifters. *Journal of Strength and Conditioning Research*, *30*(3), 784-791. <https://doi.org/10.1519/JSC.0000000000001165>
