# Supplementary material for: The Applied Sport Science and Medicine of Powerlifting and Para Powerlifting: A Systematic Scoping Review with Recommendations for Future Research
Source: Sports Med. 2025 Sep 9;55(11):2849–77. doi: 10.1007/s40279-025-02305-3 (PMC12559058; doi:10.1007/s40279-025-02305-3)
Supplement: Supplementary file 3 — Supplementary file3 (DOCX 51 KB) [file 40279_2025_2305_MOESM3_ESM.docx]

**Supplementary Table S3.** Characteristics, outcomes measures, and key findings of ‘competition’ studies (n = 45)

| **Study** | **Cohort and sample size (n); age; body mass (where specified)** | **Competitive characteristics: para status; division; weight class; age category (where specified)** | **Study aim(s)** | **Outcome measures** | **Key findings** |
| --- | --- | --- | --- | --- | --- |
| Age-related declines in anaerobic muscular performance: weightlifting and powerlifting (Anton et al., 2004) | Age-group American records for weightlifting and powerlifting | Non-para; open-69 yrs | To determine the retrospective and interactive effects of age and sex on peak anaerobic muscular performance in weightlifting and powerlifting | Snatch, clean and jerk, squat, bench press, and deadlift American record | Peak anaerobic muscular power decreases progressively with age, this decline is greater in tasks requiring more complex and powerful movements (weightlifting), the decline is greater in females than males in weightlifting only, and upper- and lower-body muscular power decline at a similar rate |
| Comparison of the relative strength among the different weight categories of power lifters (Bal et al., 2010) | 54 male All India Inter-university Power Lifting Championship powerlifters | Non-para; various weight categories | To compare relative strength among the different weight categories of powerlifters | Sum of the best 3 lifts | Athletes in heavier weight classes are stronger |
| Analysis of USA Powerlifting Federation data from January 1, 2012-June 11, 2016 (Ball & Weidman, 2018) | 21953 USA Powerlifting Federation athletes | Non-para; all divisions; all weight classes; all age categories | To compare raw vs. non-raw athletes and men vs. women | Distribution of sex, weight, ages, and totals | There is a large amount of variation in age, weight, and sex of athletes |
| How do intracompetition strategies differ between para powerlifting podium and nonpodium athletes? A retrospective, cross-sectional analysis (Beaumont et al., 2024) | 6628 individual entries; podium: 31.4 ± 8.4 yrs, 62.8 ± 18.4 kg; non-podium: 33.0 ± 9.9 yrs, 60.1 ± 17.3 kg | Para; all divisions; 8 weight classes; all age categories | To investigate the intracompetition strategies used by para powerlifters | Weight selection attempts and success/failure | High-performing para powerlifters tend to choose attempt weights that prioritise the successful completion of all 3 intracompetition lifts |
| System for evaluating powerlifting and other multievent performances (Bishop et al., 2018) | The top 28-50 top scores (weight lifted) in the data set | Non-para; raw | To develop a method of scoring powerlifting which provides equal weighting for all 3 lifts and allows for determination not only of the best lifter among weight classes but also between women and men | Weight lifted in kilograms | Coaches can use z-scores from data to assess and compare athletes |
| Performance of disabled weight lifters vs. training service length (Bolach & Wardega, 2008) | 24 male (15-50 yrs) and 5 female (16-24 yrs) Polish national level powerlifters | Para; all divisions; 4 weight classes; all age categories | To determine correlations between the length of training service and results of the Polish national team of disabled powerlifters from 2004-2006, using Haleczka’s index of actual fitness | Training service length; weight lifted in kilograms; Haleczki Score | A strong and statistically significant correlation between training length and results was observed in lifters with shorter training durations, but this correlation declined with increase of the service length |
| Powerlifting score prediction using a machine learning method (Chau, 2021) | 5500 international level male powerlifters’ results from 1972-2017; 20-35 yrs | Non-para | To study the best performance of powerlifters to develop a model for predicting the best score, based on the age, weight, and field score | Age; body mass; weight class; best squat; best bench press; best deadlift | The proposed method can effectively predict the score of powerlifters, providing a reliable basis for experts to judge the results before the competition |
| A gravitational-double layer extreme learning machine and its application in powerlifting analysis (Chau et al., 2019a) | 1280 international level female powerlifters’ results from 1972-2017 | Non-para | To explore the relationship between age, body mass, and best performance of female powerlifters, and establish a powerlifting results prediction model | Age; body mass; weight class; best squat; best bench press; best deadlift | The algorithm can predict the performance and provide an effective predictive aid for the powerlifting competition |
| The effects of age and body weight on powerlifters: an analysis model of powerlifting performance based on machine learning (Chau et al., 2019b) | 1700 international level male powerlifters’ results from 1972-2017 | Non-para | To analyse the effects of age and body mass on the performance of male powerlifters and explore the relationship between them, and establish a male powerlifting performance analysis model | Age; body mass; best squat | The method can effectively predict the best performance of powerlifters, providing a promising analytical tool for powerlifting |
| Adjusting powerlifting performances for differences in body mass (Cleather, 2006) | 1995-2004 IPF World Championships results (300 males, 270 females) | Non-para; raw tested; all weight classes | To use a large data set to explore the relationship between powerlifting performance and body mass | Body mass; squat, bench press, and deadlift results | The relationship between powerlifting performance and body mass can be modelled by an equation, where the constants are determined by the type of lift and the sex of the lifter, and with no body mass bias |
| Predictors of competitive success of national-level powerlifters: a multilevel analysis (Coker et al., 2018) | USA Powerlifting Raw Nationals Championships results (1759 male and 772 female powerlifters) | Non-para; raw tested; all weight classes | To evaluate to what extent consistency in each given lift as well as differences in body mass may be predictive of competitive success in national-level powerlifters | Body mass; weight class; attempted and successful squat, bench press, and deadlift attempts; total; Wilks score | Increased body mass within a weight class significantly increased Wilks points; the number of successful squats and bench presses were significant positive predictors of Wilks points, but not deadlifts |
| Allometric modelling of the bench press and squat: who is the strongest regardless of body mass? (Dooman & Vanderburgh, 2000) | 30 male powerlifting world record holders | Non-para; raw tested; all weight classes except super-heavyweight; open | To determine whether the allometric method provides an appropriate fit to men’s powerlifting world records | Squat, bench press, and deadlift world records; weight class | The allometric modelling approach appears to be a satisfactory method of determining the strongest body mass-adjusted men in the bench press and squat, but not the deadlift |
| Efficiency of the Wilks and IPF Formulas at comparing maximal strength regardless of bodyweight through analysis of the Open Powerlifting database (Ferland et al., 2020) | 17423 male and 9049 female powerlifters’ best performance | Non-para; 22346 classic, 4126 single-ply | To analyse the Open Powerlifting database in order to compare the efficiency of the already established Wilks Formula to the new IPF Formula in determining the champion of champions for powerlifters of the same sex and division | Best squat, bench press, deadlift, and total; division; weight class; Wilks score; IPF Formula score | The IPF Formula is more efficient at comparing women’s weight classes, while the Wilks Formula is more efficient at comparing men’s weight classes, for both divisions |
| Male powerlifting performance described from the viewpoint of complex systems (García-Manso et al., 2008) | 1812 male International Powerlifting Federation powerlifters’ results from 2003-2005 | Non-para; raw tested; all weight categories | To examine the factors governing performance in powerlifting | Weight class; best squat, bench press, deadlift, and total | Performance in male powerlifting behaves as a self-organised system with non-linear interactions between its components; multiple internal and external elements must condition changes in a competitor’s score, the most important being body mass, body size, the number of practitioners, and the concurrency of favourable factors in one individual |
| The performance of powerlifting athletes during their lifetime (Hernández Ugalde, 2022) | International Powerlifting Federation results from 1972-2020 (sample size NR) | Non-para; tested – equipped, classic, equipped bench press, classic bench press | To discover the performance dynamic during the lifetime of powerlifting and bench press athletes of both genders, as well as for the different competition categories | Wilks score; IPF points; IPF GoodLift Points | The performance for all categories of men and women in powerlifting and bench press starts with exponential growth from youth until close to the third decade, showing a peak performance between 27.67 to 31.50 years of age, and then a linear decrease during maturation and older age |
| Impacts of squat attempt weight selection and success on powerlifting performance (Howells et al., 2022) | 6594 male and 4043 female Powerlifting Australia competition entries | Non-para; raw; 47-120 kg weight classes; open and masters | To analyse powerlifting competition data to assess the relationship between squat attempts, the success of each lift attempt, and weight increase between attempts with winning | Squat attempts, success, winning/losing | A powerlifting athlete’s odds of winning a competition overall are significantly increased by selecting a larger opening squat attempt weight than competitors and completing the attempt successfully; the opening squat may be one of the most important lifts during competition |
| Sex segregation in strength sports: Do equal-sized muscles express the same levels of strength between sexes? (Kataoka et al., 2023) | Study 1: muscle thickness data sets from 454 male and 509 female non-powerlifters, study 2: International Powerlifting Federation World Championship results from 2015-2019 | Non-para; classic; males: 59, 66, 74, 83 kg, females: 57, 63, 72, 84 kg; open | To investigate the possible sex differences in various strength outcomes with pair-matched muscle size, and to compare the performances of the smallest male weight class in the adult division of the International Powerlifting Federation to different weight classes in females | Muscle thickness and strength; best squat, bench press, and deadlift from each competition for males and females of each weight class | Strength assessments were greater in males than females with pair-matched muscle thickness, regardless of contraction types; males in the lightest weight division of the International Powerlifting Federation largely outperformed females in heavier weight divisions |
| Powerlifting junior athletes with physical impairment: performance analysis considering gender, origin of impairment, competitive achievement and impairment type (Kons et al., 2023) | 54 male and 29 female Paralympic powerlifters | Para; all weight classes; junior | To explore whether gender, origin of impairment, and impairment type could influence the performance of junior powerlifters athletes with physical impairment | Competitive results, impairment characteristics | For absolute and relative loads, males were stronger when compared to females; chronological age and performance load were not influenced by origin of impairment, impairment type, or competition achievement |
| Long-term adaptations in the squat, bench press, and deadlift: Assessing strength gain in powerlifting athletes (Latella et al., 2022) | 1270 male and 625 female powerlifters’ results from 407 Powerlifting Australia meets; mean 28 yrs; mean 82.3 kg | Non-para; raw | To investigate longitudinal upper- and lower-body strength adaptations | Highest successful squat, bench press, and deadlift | There were differences in strength gain between upper- and lower-body lifts, but not sex differences in the change in strength; the strongest males likely gain strength more slowly than weaker counterparts |
| Factors affecting powerlifting performance: An analysis of age- and weight-based determinants of relative strength (Latella et al., 2018) | 850 male and 518 female Powerlifting Australia powerlifters’ results; 15-78 yrs | Non-para; all weight classes; all age categories | To analyse relative strength subject to body mass and age both within and between male and female competitive powerlifters | Best squat, bench press, and deadlift | Relative strength was greater for males across all lifts; relative strength tended to decrease with increasing body mass for males and females, and with increasing age for males and females |
| Differences in strength performance between novice and elite athletes: Evidence from powerlifters (Latella et al., 2019) | 2137 local, national, and international level powerlifters’ results; 14-82 yrs; 37.3-201.0 kg | Non-para; all weight classes; all age categories | To evaluate the differences in strength and performance between novice, sub-elite, and elite strength athletes in powerlifting | Highest successful squat, bench press, deadlift, and total | A moderate to large increase in performance was observed for all weight classes between local and national level; no meaningful differences were observed between local and national, and national and international level when compared using age; no meaningful differences were observed between national to international competitions using weight classes |
| Using powerlifting athletes to determine strength adaptations across ages in males and females: A longitudinal growth modelling approach (Latella et al., 2024) | 9259 powerlifters’ results | Non-para; raw tested; all weight classes; all age categories | To identify differences in the rate and magnitude of strength adaptation based on age category and weight class for overall strength (competition total), and the squat, bench press, and deadlift, respectively | Best squat, bench press, deadlift, and total | The greatest strength gains were in the earliest phase of powerlifting participation; females tended to display faster progression, possibly because of lower baseline strength |
| Long-term strength adaptation: A 15-year analysis of powerlifting athletes (Latella et al., 2020) | 1271 male (26.8 ± 8.9 yrs; 88.7 ± 17.3 kg) and 626 female (30.6 ± 10.19 yrs; 69.1 ± 15.2 kg) Powerlifting Australia powerlifters’ results from 2003-2018 | Non-para; raw tested; all weight classes; all age categories | To investigate the rate of strength gain (per day) in both male and female powerlifters over a 15-year period | Highest successful squat, bench press, deadlift, and total | Strength gain/day was similar between sexes; the strongest males showed a lower rate of strength improvement compared with least strong males |
| Changes in steroid hormones during an international powerlifting competition (Le Panse et al., 2012) | 8 male (20.1 ± 0.7 years; 67.0 ± 3.5 kg) and 11 female (19.1 ± 0.8 yrs; 59.0 ± 2.0 kg) elite powerlifters | Non-para | To examine variations in saliva cortisol, DHEA, and testosterone in elite powerlifters during a World Championship and the correlations between hormone and performance parameters in order to determine whether hormone concentrations may be used as performance predicting factors for elite powerlifting athletes | Cortisol, DHEA, and testosterone concentrations; weight lifted in kilograms; Wilks formula performance index; athlete rank. | After the competition, cortisol and DHEA were significantly increased in male and female athletes, whereas testosterone was only significantly increased in men; performance was significantly related to end-competition cortisol levels in women and to end-competition testosterone levels in men |
| Cortisol, DHEA, and testosterone concentrations in saliva in response to an international powerlifting competition (Le Panse et al., 2010) | 13 male (37.7 ± 2.4 yrs; 95.8 ± 9.1 kg) and 13 female (34.2 ± 2.5 yrs; 62.7 ± 6.0 kg) elite powerlifters | Non-para | To examine non-invasively the acute steroid hormone response to the bench press in elite athletes during an official international powerlifting competition and to determine whether salivary hormone concentrations could be accurate predictors of performance | Cortisol, DHEA, and testosterone concentrations; Wilks formula performance index | Increased stress did not predict performance; cortisol levels increased significantly after the bench press attempts; DHEA levels increased significantly after the bench press in women but not in men; no change in testosterone was observed |
| Performance of Paralympic powerlifting records holders: An analysis considering origin of impairment, sex, and competitive level (Lopes-Silva et al., 2023) | 30 male and 30 female current world and Paralympic para powerlifting record holders’ records | Para; 20 junior, 40 senior | To explore the performance differences for current world and Paralympic record holders according to origin of impairment (acquired and congenital) and to examine the relationships between origin of impairment, level of competition, and sex, and to describe the performance progression of Paralympic powerlifting record holders | Records | The origin of impairment did not influence the absolute and relative load of male and female recordists; for males, acquired athletes were older and heavier when compared with congenital athletes |
| Para powerlifting performance: A retrospective analysis considering origin of impairment, sport classification, and sex (Lopes-Silva et al., 2024) | 4613 male and 2178 female Paralympic powerlifters’ results from 2013-2020 | Para | To verify the retrospective performance of para powerlifting athletes over 8 years, considering sex and the origin of impairment (acquired and congenital), and whether the origin of impairment, competition achievement, and sex could be influenced by the chronological age of para powerlifters | Performance | Males were stronger than females throughout the years with acquired impairment being stronger than congenital impairment; there was a significant association between competition achievement and sports class classification, with a higher number of medals for limb deficiency than other sports classes |
| Chronological age and performance in Paralympic powerlifters: differences between sexes, competition, and weight categories (Lopes-Silva et al., 2023a) | 1985 male (33.2 ± 8.6 yrs) and 1122 female (32.2 ± 7.5 yrs) international level Paralympic powerlifters’ results | Para | To quantify the chronological age and performance of Paralympic powerlifters competing at the elite level, and to examine age-related differences between sexes, weight categories, and competition achievements | Results; age; weight class | Males were older and stronger than females; medallists were younger than non-medallists; para powerlifters competing in lighter categories were younger when compared to heavier categories, and their relative load was heavier |
| Is there an optimal interval for medal winning performance in World Para Powerlifting competition? (Lopes-Silva et al., 2023b) | 2993 male and 1683 female World Para Powerlifting powerlifters’ results from 2014-2020 | Para | To determine the optimal interval between competitions to define success in World Para Powerlifting events | Results | The para powerlifting athletes participating in the World Cup events, World Championships, and Paralympic Games share an optimal interval for success of approximately 21–31 weeks and results from the World Championships and Paralympic Games, which can also be optimized with an interval of 40 week or more |
| Modeling the influence of body size on weightlifting and powerlifting performance (Marković & Sekulić, 2006) | 170 International Powerlifting Federation powerlifters’ results from 2000-2003 | Non-para; male weight classes up to 110 kg, female weight classes up to 75 kg | To examine if lifting performance in both weightlifting and powerlifting scale with body mass in line with the theory of geometric similarity, and whether there are any gender differences in the allometric relationship between lifting performance and body size | Allometric and second-order polynomial relationship between lifting performances | Allometric modelling of powerlifting results for body mass does not provide a size-independent index of lifting performance; instead, it exhibits a favourable bias toward middleweight lifters in most lifting data analysed |
| Knee wraps are detrimental to the maximal squat performance of powerlifters competing in lower weight classes (Naylor et al., 2023) | 270268 male and 136530 female powerlifters’ results, 26576 male and 11468 female powerlifters’ results | Non-para; raw; all weight classes | To use Open Powerlifting data to measure the net benefit (weight added to the 1RM squat) of knees wraps for male and female lifters across a standard set of weight classes | Best squat | Only males in the heaviest three weight classes (105, 120, and 120+ kg), as well as females in the heaviest weight class (84+ kg), stand to gain any benefit from the use of knee wraps in competition |
| The rates of null and valid movements in parapowerlifting in international competitions (Ortega et al., 2023) | 2084 Paralympic powerlifters’ results | Para | To examine the null movements magnitude during 12 international events and to investigate if variables such as weight category, gender, or country of origin are related to lifting efficiency | Attempt outcome | There is a high rate of null movements in international Paralympic powerlifting competitions regardless of gender, country, or participants’ category |
| Effect of competition frequency on strength performance of powerlifting athletes (Pearson et al., 2020) | 560 male and 440 female Powerlifting Australia powerlifters’ results in 2017; 30 ± 10 yrs; 81.0 ± 20.3 kg | Non-para; raw | To evaluate the relationship between strength and competition frequency in male and female powerlifting athletes | Best squat, bench press, deadlift, and total | Powerlifting athletes who compete multiple times per year are more likely to achieve higher totals; however, there is an upper limit to the number of competitions (4 per year) that seems to allow a performance increase |
| Sex differences in athletic performance (Ransdell & Wells, 1999) | Natural Athletic Strength Association and US Powerlifting Federation records (sample size NR) | Non-para | To analyse sex differences in performance relative to the human energy system | Squat, bench press, deadlift, and total records | In the teenage division, average bench press performance differences are only about 30%, while those in the open divisions average 45%; when strength differences are examined as weight lifted per kg of body weight, men are able to consistently lift about 11 kg per kg of body weight, whereas the amount lifted by women per kg of body weight varies between 6.75 and 8.93 |
| Expert consensus on classification and performance in Paralympic powerlifting (Rum et al., 2024) | 26 Paralympic powerlifting experts (athletes, coaches, classifier, administrators, medical staff, or referees) | Para | To gather expert opinions on the classification and performance aspects of Paralympic powerlifting through the Delphi technique, laying an objective basis for evaluating the current athlete classification process and providing further indications for performance-oriented research | Subjective measures of opinions on classification and performance issues | Experts agreed that existing classification and competition systems in Paralympic powerlifting do not align with Paralympic standards; impairments from neurological conditions and those causing anthropometric changes were suggested to have opposing performance impacts |
| Peak age and performance trajectories in para powerlifters (Severin et al., 2023) | 2079 elite powerlifters | Para; all divisions; all weight classes; all age categories | To quantify the age-related trajectory of performance in high level para powerlifters | Weight lifted; body weight; bodyweight category; finishing rank; date of birth; competition level and type | Para powerlifters in heavier bodyweight categories were older than those in lighter categories; men typically lifted their heaviest weight at age 36, and women at age 41; high performers reached their peak weight lifted 2.6 years earlier than lower performers; the highest bodyweight category weight lifted was achieved between ages 31 and 35, earlier than the peak weight lifting age for most lifters |
| Peak age and performance progression in world-class weightlifting and powerlifting athletes (Solberg et al., 2019) | Powerlifting World Championships results from 2003-2017 and weightlifting World Championships and Olympic Games results from 1998-2017 (4385 athletes) | Non-para; tested | To quantify peak age and improvements over the preceding years to peak age in elite weightlifting and powerlifting athletes as a function of athlete performance level, sex, and event | Best squat, bench press, and deadlift or snatch and clean and jerk | Peak age was 35 for powerlifters and 26 for weightlifters; men showed a possibly higher peak age than women in weightlifting and a possibly lower peak age in powerlifting |
| Weight selection attempts of elite classic powerlifters (Travis et al., 2021) | 66 male and 43 female World Classic Powerlifting Championships powerlifters’ results from 2012-2019 | Non-para; raw tested; all weight classes; open category | To observe attempts selected by elite male and female classic powerlifters who successfully completed all attempts at an International Powerlifting Federation World Classic Championship, and to compare weight selection attempts between sexes | Competition squat, bench press, and deadlift attempt selection | Selecting an opener of ~91% of the expected third attempt, followed by ~5% increase from first attempt to second attempt, and ~3% increase from second attempt to third attempt represent the typical weight selection attempts used across lifts by elite classic powerlifters competing in the World Championships |
| Paralympians are stronger than you know: a comparison of para and nondisabled powerlifting bench press world records (van den Hoek et al., 2023a) | International Powerlifting Federation (30 males and 30 females) and World Para Powerlifting (24 males and 22 females) world records as of 2021 | Para and non-para; bench press only, raw tested; all weight classes | To explore world records from World Para Powerlifting and International Powerlifting Federation bench press-only competitions | Bench press world records | The physical impairments experienced by World Para Powerlifting world record holders do not appear to compromise bench press strength compared with able-bodied athletes; indeed, World Para Powerlifting world record holders often possess greater relative and absolute strength than their International Powerlifting Federation counterparts |
| Analysis of competition performance leading to success at the International Powerlifting Federation World Championships between 2013 and 2019 (van den Hoek et al., 2023b) | 734 male (29.8 ± 5 yrs; 95.1 ± 28.1 kg) and 578 female (31.8 ± 6.9 yrs; 67.4 ± 20.1 kg) international level powerlifters | Non-para; raw tested; all weight classes; open | To analyse opening attempt weight selections, report absolute maximum successful attempt weights and ranges, and identify trends and differences in the number of successful lifts of winners and non-winners | Number of successful attempts; relative attempt weight selection; maximum successful attempt weight | The odds of winning a competition were increased by 5.9 times for men and by 1.5 times for women when successfully completing 8 or 9 of 9 lift attempts |
| Normative data for the squat, bench press and deadlift exercises in powerlifting: Data from 809,986 competition entries (van den Hoek et al., 2024a) | 571650 male and 238336 female powerlifters’ results from 1968-2022; 30.5 ± 13.3 yrs; 82.4 ± 21.3 kg | Non-para; raw tested | To develop contemporary strength norms for the squat, bench press, and deadlift using powerlifters competing in un-equipped, drug-tested competitions | Squat, bench press, and deadlift results | These findings provide a comprehensive, accurate and precise representation of strength for drug-tested, unequipped powerlifters in each category and serve as a point of reference for other trained population groups |
| Powerlifting participation and engagement across all ages: A retrospective, longitudinal, population analysis with comparison to community strength norms (van den Hoek et al., 2024b) | 14720 male and 6804 female Australian powerlifters’ results from 1968-2022; 28.84 ± 10.26 yrs; 82.59 ± 20.79 kg | Non-para; raw, wraps, and single-ply; all age categories | To examine the longitudinal participation trends of Australian powerlifting, with further analysis to identify age- and sex-based participation trends, and to explore the strength of powerlifting participants within various age classifications compared to reported community-based norms | Squat, bench press, and deadlift results | Exponential growth was seen in competition entries from 115 in 1981, to 759 in 1994, 1014 in 2011, and to 6803 in 2022; at first participation 18–25-year-olds followed by ≥36 years were most represented; strength comparison to available population norms demonstrates superior upper- and lower-body strength |
| What are the odds? Identifying factors related to competitive success in powerlifting (van den Hoek et al., 2022) | 6567 male (28 ± 10 yrs; 89.5 ± 18.3 kg) and 4032 female (31 ± 11 yrs; 69.5 ± 15.7 kg) Powerlifting Australia powerlifters’ results from 2010-2019 | Non-para; classic; all weight classes | To determine differences in weights achieved by winning and non-winning raw powerlifting athletes, and to explore how age, sex, body weight, time competing, and relative opening attempt weight influence the likelihood of successful competition performance | Competition results | Athletes who had larger first lift attempts and competed for a longer period had an increase likelihood of winning; age was associated with increased odds of success per additional year of age for males, but not females |
| Validation of the Wilks powerlifting formula (Vanderburgh & Batterham, 1999) | International Powerlifting Federation male and female world records holders as of 1998 and the top two performers in 1996 and 1997 International Powerlifting Federation World Championships (30 males and 27 females for each event) | Non-para; raw tested | To examine residuals bias to verify that the adjusted Wilks score does, in fact, lead to no systematic bias based on body mass and to apply a more theoretically supportable allometric model to the same data and compare/evaluate residuals bias with that of the Wilks approach | Records and competition results | There is no bias for men’s or women’s bench press and total; there is a favourable bias toward intermediate weight class lifters in the women’s squat with no bias for men’s squat; there is a linear unfavourable bias toward heavier men and women in the deadlift; the allometric approach indicated a bias against light and heavy men and women which may be considered acceptable given that half as many lifters are found in the lightest and heaviest weight classes as in the intermediate weight classes |
| The influence of compressive gear on maximal load lifted in competitive powerlifting (Wilk et al., 2020) | 63 male and 57 female powerlifters’ results from 2013-2019 and raw and equipped world records | Non-para; raw and equipped; males: 52-120+ kg, females: 47-84+ kg | To analyse and compare the results of the raw and equipped powerlifting divisions based on the results of world championships and current world records | Squat, bench press, and deadlift results and world records | There were significantly higher results in the squat and bench press for equipped compared to the raw division; there were no significant differences in the results of world records in the deadlift |

**References**

Anton, M. M., Spirduso, W. W., & Tanaka, H. (2004). Age-related declines in anaerobic muscular performance: Weightlifting and powerlifting. *Medicine and Science in Sports and Exercise*, *36*(1), 143-147. <https://doi.org/10.1249/01.mss.0000106283.34742.be>

Bal, B. S., Yadav, S., & Sinha, A. (2010). Comparison of the relative strength among the different weight categories of power lifters. *Journal of Physical Education and Sport*, *27*(2), 126-130.

Ball, R., & Weidman, D. (2018). Analysis of USA Powerlifting Federation data from January 1, 2012–June 11, 2016. *Journal of Strength and Conditioning Research*, *32*(7), 1843-1851. <https://doi.org/10.1519/JSC.0000000000002103>

Beaumont, P., Garrett, J., & van den Hoek, D. (2024). How do intracompetition strategies differ between para powerlifting podium and nonpodium athletes? A retrospective, cross-sectional analysis. *Journal of Strength and Conditioning Research*, *38*(3), 584-591. <https://doi.org/10.1519/JSC.0000000000004665>

Bishop, P. A., Williams, T. D., Heldman, A. N., & Vanderburgh, P. M. (2018). System for evaluating powerlifting and other multievent performances. *Journal of Strength and Conditioning Research*, *32*(1), 201-204. <https://doi.org/10.1519/JSC.0000000000001808>

Bolach, E., & Wardega, A. (2008). Performance of disabled weight lifters vs. training service length. *Fizjoterapia*, *16*(4), 76-97. <https://doi.org/10.2478/v10109-009-0048-1>

Chau, V. H. (2021). Powerlifting score prediction using a machine learning method. *Mathematical Biosciences and Engineering*, *18*(2), 1040-1050. <https://doi.org/10.3934/mbe.2021056>

Chau, V. H., Vo, A. T., & Le, B. T. (2019a). A gravitational-double layer extreme learning machine and its application in powerlifting analysis. *IEEE Access*, *7*, 143990-143998. <https://doi.org/10.1109/ACCESS.2019.2944877>

Chau, V. H., Vo, A. T., & Le, B. T. (2019b). The effects of age and body weight on powerlifters: An analysis model of powerlifting performance based on machine learning. *International Journal of Computer Science in Sport*, *18*(3), 89-99. <https://doi.org/10.2478/ijcss-2019-0019>

Cleather, D. J. (2006). Adjusting powerlifting performances for differences in body mass. *Journal of Strength and Conditioning Research*, *20*(2), 412-421. <https://doi.org/10.1519/R-17545.1>

Coker, N. A., Varanoske, A. N., Baker, K. M., Hahs-Vaughn, D. L., & Wells, A. J. (2018). Predictors of competitive success of national-level powerlifters: A multilevel analysis. *International Journal of Performance Analysis in Sport*, *18*(5), 796-805. <https://doi.org/10.1080/24748668.2018.1519751>

Dooman, C. S., & Vanderburgh, P. M. (2000). Allometric modeling of the bench press and squat: Who is the strongest regardless of body mass? *Journal of Strength and Conditioning Research*, *14*(1), 32-36.

Ferland, P.-M., Allard, M.-O., & Comtois, A. S. (2020). Efficiency of the Wilks and IPF Formulas at comparing maximal strength regardless of bodyweight through analysis of the Open Powerlifting database. *International Journal of Exercise Science*, *13*(4), 567-582.

García-Manso, J. M., Martín-González, J. M., Da Silva-Grigoletto, M. E., Vaamonde, D., Benito, P., & Calderón, J. (2008). Male powerlifting performance described from the viewpoint of complex systems. *Journal of Theoretical Biology*, *251*(3), 498-508. <https://doi.org/10.1016/j.jtbi.2007.12.010>

Hernández Ugalde, J. A. (2022). The performance of powerlifting athletes during their lifetime. *MHSalud*, *19*(1). <https://doi.org/10.15359/mhs.19-1.4>

Howells, R. J., Spathis, J. G., Pearson, J., Latella, C., Garrett, J. M., Owen, P. J., & van den Hoek, D. J. (2022). Impacts of squat attempt weight selection and success on powerlifting performance. *Journal of Sports Medicine and Physical Fitness*, *62*(4), 476-484. <https://doi.org/10.23736/S0022-4707.21.12140-1>

Kataoka, R., Spitz, R. W., Wong, V., Bell, Z. W., Yamada, Y., Song, J. S., Hammert, W. B., Dankel, S. J., Abe, T., & Loenneke, J. P. (2023). Sex segregation in strength sports: Do equal-sized muscles express the same levels of strength between sexes? *American Journal of Human Biology*, *35*(5), e23862. <https://doi.org/10.1002/ajhb.23862>

Kons, R. L., Franchini, E., & Lopes-Silva, J. P. (2023). Powerlifting junior athletes with physical impairment: Performance analysis considering gender, origin of impairment, competitive achievement and impairment type. *Journal of Science in Sport and Exercise*. <https://doi.org/10.1007/s42978-023-00255-8>

Latella, C., Owen, P. J., Davies, T., Spathis, J., Mallard, A., & Van Den Hoek, D. (2022). Long-term adaptations in the squat, bench press, and deadlift: Assessing strength gain in powerlifting athletes. *Medicine and Science in Sports and Exercise*, *54*(5), 841-850. <https://doi.org/10.1249/MSS.0000000000002858>

Latella, C., Van den Hoek, D., & Teo, W.-P. (2018). Factors affecting powerlifting performance: An analysis of age- and weight-based determinants of relative strength. *International Journal of Performance Analysis in Sport*, *18*(4), 532-544. <https://doi.org/10.1080/24748668.2018.1496393>

Latella, C., van den Hoek, D., & Teo, W.-P. (2019). Differences in strength performance between novice and elite athletes: Evidence from powerlifters. *Journal of Strength and Conditioning Research*, *33 Suppl 1*, S103-S112. <https://doi.org/10.1519/JSC.0000000000002823>

Latella, C., van den Hoek, D., Wolf, M., Androulakis-Korakakis, P., Fisher, J. P., & Steele, J. (2024). Using powerlifting athletes to determine strength adaptations across ages in males and females: A longitudinal growth modelling approach. *Sports Medicine*, *54*(3), 753-774. <https://doi.org/10.1007/s40279-023-01962-6>

Latella, C., Wei-Peng, T., Spathis, J., & van den Hoek, D. (2020). Long-term strength adaptation: A 15-year analysis of powerlifting athletes. *Journal of Strength and Conditioning Research*, *34*(9), 2412-2418. <https://doi.org/10.1519/jsc.0000000000003657>

Le Panse, B., Labsy, Z., Baillot, A., Vibarel-Rebot, N., Parage, G., Albrings, D., Lasne, F., & Collomp, K. (2012). Changes in steroid hormones during an international powerlifting competition. *Steroids*, *77*(13), 1339-1344. <https://doi.org/10.1016/j.steroids.2012.07.015>

Le Panse, B., Vibarel-Rebot, N., Parage, G., Albrings, D., Amiot, V., De Ceaurriz, J., & Collomp, K. (2010). Cortisol, DHEA, and testosterone concentrations in saliva in response to an international powerlifting competition. *Stress*, *13*(6), 528-532. <https://doi.org/10.3109/10253891003743440>

Lopes-Silva, J. P., Franchini, E., & Kons, R. (2023). Performance of Paralympic powerlifting records holders: An analysis considering origin of impairment, sex, and competitive level. *American Journal of Physical Medicine and Rehabilitation*, *102*(11), 1034-1039. <https://doi.org/10.1097/PHM.0000000000002153>

Lopes-Silva, J. P., Franchini, E., & Kons, R. (2024). Para powerlifting performance: A retrospective analysis considering origin of impairment, sport classification, and sex. *American Journal of Physical Medicine and Rehabilitation*, *103*(4), 356-362. <https://doi.org/10.1097/PHM.0000000000002307>

Lopes-Silva, J. P., Richardson, D., & Franchini, E. (2023a). Chronological age and performance in Paralympic powerlifters: Differences between sexes, competition, and weight categories. *Journal of Science in Sport and Exercise*, *5*(1), 53-61. <https://doi.org/10.1007/s42978-021-00149-7>

Lopes-Silva, J. P., Richardson, D., Fukuda, D. H., & Franchini, E. (2023b). Is there an optimal interval for medal winning performance in World Para Powerlifting competition? *American Journal of Physical Medicine and Rehabilitation*, *102*(2), 172-174. <https://doi.org/10.1097/PHM.0000000000001935>

Marković, G., & Sekulić, D. (2006). Modeling the influence of body size on weightlifting and powerlifting performance. *Collegium Antropologicum*, *30*(3), 607-613.

Naylor, A., Ashkanfar, A., Xiaoxiao, L., & English, R. (2023). Knee wraps are detrimental to the maximal squat performance of powerlifters competing in lower weight classes. *Kinesiology*, *55*(2), 282-288. <https://doi.org/10.26582/k.55.2.8>

Ortega, J. A. F., Ramirez, J. J. A., Galvis, R. N., Cuartas, L. A. H., & Sánchez, M. F. M. (2023). The rates of null and valid movements in parapowerlifting in international competitions. *Revista Brasileira de Ciencias do Esporte*, *45*. <https://doi.org/10.1590/rbce.45.e20230008>

Pearson, J., Spathis, J. G., van den Hoek, D. J., Owen, P. J., Weakley, J., & Latella, C. (2020). Effect of competition frequency on strength performance of powerlifting athletes. *Journal of Strength and Conditioning Research*, *34*(5), 1213-1219. <https://doi.org/10.1519/jsc.0000000000003563>

Ransdell, L. B., & Wells, C. L. (1999). Sex differences in athletic performance. *Women in Sport and Physical Activity Journal*, *8*(1), 55-81. <https://doi.org/10.1123/wspaj.8.1.55>

Rum, L., Romagnoli, R., Lazich, A., Sciarra, T., Balletti, N., Piacentini, M. F., Boraschi, A., & Bergamini, E. (2024). Expert consensus on classification and performance in Paralympic powerlifting: A Delphi study. *American Journal of Physical Medicine and Rehabilitation*. <https://doi.org/10.1097/PHM.0000000000002478>

Severin, A. C., Baumgart, J. K., Haugen, T., & Hogarth, L. (2023). Peak age and performance trajectories in para powerlifters. *American Journal of Physical Medicine and Rehabilitation*, *102*(7), 645-652. <https://doi.org/10.1097/PHM.0000000000002051>

Solberg, P. A., Hopkins, W. G., Paulsen, G., & Haugen, T. A. (2019). Peak age and performance progression in world-class weightlifting and powerlifting athletes. *International Journal of Sports Physiology and Performance*, *14*(10), 1357-1363. <https://doi.org/10.1123/ijspp.2019-0093>

Travis, S. K., Zourdos, M. C., & Bazyler, C. D. (2021). Weight selection attempts of elite classic powerlifters. *Perceptual and Motor Skills*, *128*(1), 507-521. <https://doi.org/10.1177/0031512520967608>

van den Hoek, D., Garrett, J., Howells, R., & Latella, C. (2023a). Paralympians are stronger than you know: A comparison of para and nondisabled powerlifting bench press world records. *Journal of Strength and Conditioning Research*, *37*(2), 452-456. <https://doi.org/10.1519/JSC.0000000000004251>

van den Hoek, D., Garrett, J., Travis, S. K., Oxnard, K., Howells, R., Owen, P., & Latella, C. (2023b). Analysis of competition performance leading to success at the International Powerlifting Federation World Championships between 2013 and 2019. *Journal of Strength and Conditioning Research*, *37*(10), e555-e562. <https://doi.org/10.1519/jsc.0000000000004488>

van den Hoek, D. J., Beaumont, P. L., van den Hoek, A. K., Owen, P. J., Garrett, J. M., Buhmann, R., & Latella, C. (2024a). Normative data for the squat, bench press and deadlift exercises in powerlifting: Data from 809,986 competition entries. *Journal of Science and Medicine in Sport*, *27*(10), 734-742. <https://doi.org/10.1016/j.jsams.2024.07.005>

van den Hoek, D. J., Mallard, A., Garrett, J. M., Beaumont, P. L., Howells, R. J., Spathis, J. G., Pearson, J., & Latella, C. (2024b). Powerlifting participation and engagement across all ages: A retrospective, longitudinal, population analysis with comparison to community strength norms. *International Journal of Sports Science and Coaching*. <https://doi.org/10.1177/17479541241244481>

van den Hoek, D. J., Owen, P. J., Garrett, J. M., Howells, R. J., Pearson, J., Spathis, J. G., & Latella, C. (2022). What are the odds? Identifying factors related to competitive success in powerlifting. *BMC Sports Science, Medicine and Rehabilitation*, *14*(1), 1-11. <https://doi.org/10.1186/s13102-022-00505-2>

Vanderburgh, P. M., & Batterham, A. M. (1999). Validation of the Wilks powerlifting formula. *Medicine and Science in Sports and Exercise*, *31*(12), 1869-1875. <https://doi.org/10.1097/00005768-199912000-00027>

Wilk, M., Krzysztofk, M., & Bialas, M. (2020). The influence of compressive gear on maximal load lifted in competitive powerlifting. *Biology of Sport*, *37*(4), 437-441. <https://doi.org/10.5114/biolsport.2021.100145>
