# Supplementary material for: The Applied Sport Science and Medicine of Powerlifting and Para Powerlifting: A Systematic Scoping Review with Recommendations for Future Research
Source: Sports Med. 2025 Sep 9;55(11):2849–77. doi: 10.1007/s40279-025-02305-3 (PMC12559058; doi:10.1007/s40279-025-02305-3)
Supplement: Supplementary file 4 — Supplementary file4 (DOCX 57 KB) [file 40279_2025_2305_MOESM4_ESM.docx]

**Supplementary Table S4.** Characteristics, outcomes measures, and key findings of ‘physical qualities’ studies (n = 48)

| **Study** | **Cohort and sample size (n); age; body mass (where specified)** | **Competitive characteristics: para status; division; weight class; age category (where specified)** | **Study aim(s)** | **Outcome measures** | **Key findings** |
| --- | --- | --- | --- | --- | --- |
| Comparison of isolated lumbar extension strength in competitive and noncompetitive powerlifters, and recreationally trained men (Androulakis-Korakakis et al., 2021) | 10 noncompetitive powerlifters (24 ± 3.5 yrs; 92.39 ± 15.73 kg), 13 national, divisional, or international level powerlifters (31.9 ± 7.6 yrs; 91.75 ± 18.7 kg), 36 recreationally trained males (25 ± 6.5 yrs; 81.6 ± 10 kg) | Non-para | To investigate isolated lumbar extension strength in competitive and noncompetitive powerlifters, in addition to a recreationally resistance-trained population | Squat and deadlift 1RM; subjective measures of powerlifting characteristics; isolated lumbar extension strength index, average torque, and maximum torque | Isolated lumbar extension strength does not differ between competitive and noncompetitive powerlifters, and recreationally trained men, suggesting that powerlifting style training likely does not impact isolated lumbar extension strength |
| The role of FFM accumulation and skeletal muscle architecture in powerlifting performance (Brechue & Abe, 2002) | 20 male US Men’s National Powerlifting Championship powerlifters: 7 heavy-weight (135.1 ± 26.5 kg), 6 middle-weight (78.4 ± 6.7 kg), 7 light-weight (63.9 ± 5.6 kg) | Non-para; 3 weight classes | To determine the distribution and architectural characteristics of skeletal muscle and to investigate their relationship to fat free mass accumulation and powerlifting performance | Fat free mass; limb length; muscle thickness; skeletal muscle distribution; isolated muscle thickness; fascicle pennation angle | Powerlifting performance (squat, bench press, and deadlift) was significantly related to fat free mass; greater fascicle lengths are associated with greater fat free mass accumulation and powerlifting performance |
| The effect of the squat exercise on knee stability (Chandler et al., 1989) | 27 male powerlifters (14 elite or master class), 28 male weightlifters (8 elite or master class), 30 males with little or no weight training experience | Non-para | To determine the relationship between the squat exercise and knee stability | 9 knee ligament arthrometry tests (anterior drawer, compliance index, posterior drawer, maximal manual drawer, quadriceps active drawer) | Powerlifters were significantly tighter than controls on the anterior drawer at 90 degrees of knee flexion; both powerlifters and weightlifters were significantly tighter than controls on the quadriceps active drawer at 90 degrees of knee flexion |
| Limited joint mobility in power lifters (Chang et al., 1988) | 10 male powerlifters (92.5 ± 6.8 kg), 10 age-matched males without lifting experience (72.6 ± 8.0 kg); 21-35 yrs | Non-para | To compare the flexibility of powerlifters to that of nonlifters who were not involved in competitive sports | Behind the back reach test, sit and reach test, goniometric joint range of motion measurements | The behind the back reach test and goniometric measurements of shoulder movement, elbow and wrist flexion, hip flexion and rotation, and knee flexion revealed significant limitations of flexibility for the powerlifters compared to age-matched controls; the sit and reach test was the only measurement in which the flexibility of the powerlifters exceeded that of the nonlifters |
| An evaluation of agonist:antagonist strength ratios and posture among powerlifters (Cutrufello et al., 2017) | 15 male powerlifters (35.3 ± 13.7 yrs, 98.8 ± 19.1 kg), 15 age-matched controls (34.9 ± 9.8 yrs, 96.7 ± 19.1 kg) | Non-para | To examine strength ratios among powerlifters, and the relationship between these strength ratios and posture compared with age-matched controls | Shoulder, hip, knee, and cervical agonist:antagonist strength ratios; pelvic tilt; thoracic kyphosis; lumbar lordosis; pectoralis minor length | Strength imbalances were observed at the shoulder and knee; pectoralis minor length was significantly shorter in powerlifters; there was no significant difference in thoracic kyphosis, pelvic tilt, or lumbar lordosis |
| Comparing thigh muscle cross-sectional area and squat strength among national class Olympic weightlifters, power lifters, and bodybuilders (Di Naso et al., 2013) | 5 national level weightlifters (19.40 ± 2.97; 85.74 ± 7.60), 5 national level powerlifters (33.20 ± 6.38; 87.92 ± 8.66), 5 national level bodybuilders (40.00 ± 7.31 yrs; 83.64 ± 5.47 kg) | Non-para; tested (except 1 powerlifter) | To determine if significant differences exist in the relationship between thigh muscle cross-sectional area and back squat strength among national class athletes from the sports of weightlifting, powerlifting, and bodybuilding | Mid-thigh skinfold and circumference; estimated thigh muscle cross-sectional area; back squat 1RM; relative strength (1RM back squat/body mass) | Thigh cross-sectional area was of relatively minor importance in determining back squat strength for the weightlifting and powerlifting groups, despite these groups being significantly stronger than the bodybuilding group |
| Contractile changes in knee extensor muscles after repetitive maximal isokinetic contractions in male power-lifters and untrained subjects (Ereline et al., 2004) | 11 national level powerlifters (25.5 ± 1.6 yrs; 85.8 ± 5.0 kg), 14 untrained controls (23.3 ± 1.6 yrs; 75.7 ± 3.7 kg) | Non-para | To compare changes in isometric maximal voluntary contraction and electrically evoked submaximal tetanic contraction characteristics of the knee extensor muscles after short-term repetitive maximal isokinetic knee extension exercise in powerlifters and untrained men | Isometric maximal voluntary contraction force; rate of force development; peak force; half-relaxation time of electrically evoked tetanic contraction | Powerlifters produced a significantly greater work output and fatigued faster during repetitive maximal isokinetic knee extensions compared with untrained subjects, and their recovery was more delayed |
| Relationships between anthropometry and maximal strength in male classic powerlifters (Ferland et al., 2020a) | 59 male Quebec Powerlifting Federation powerlifters | Non-para; classic; all weight classes; 1 sub-junior, 18 junior, 40 open | To quantify the relationships between body composition, anthropometric values and physical proportions, and maximal strength in classic powerlifting | Anthropometric measurements; body composition; best absolute and relative squat, bench press, deadlift, and total | There were multiple significant relationships between physical characteristics and relative and maximal strength in the squat, the bench press, the deadlift, and the total |
| The relationship between body composition measured by Dual-Energy X-Ray Absorptiometry (DEXA) and maximal strength in classic powerlifting (Ferland et al., 2020b) | 9 male and 6 female Quebec Powerlifting Federation powerlifters; 27.2 ± 4.2 yrs; 88.4 ± 20.1 kg | Non-para; classic; 3 junior, 12 open | To conduct a detailed body composition analysis using Dual-Energy X-Ray Absorptiometry (DEXA scan) on classic powerlifters to reveal the various relationships that exist between body composition variables and maximal strength measures | Body composition; best absolute and relative squat, bench press, deadlift, and total | Experience in resistance training, lean body tissue, arms mass, legs mass, bone mineral content, and bone mineral density measures were significantly correlated with most absolute and relative maximal strength measures |
| Performance and anthropometrics of classic powerlifters: Which characteristics matter? (Ferrari et al., 2022) | 51 male (18-40 yrs; 65-117 kg) and 23 female (17-41 yrs, 46-82 kg) powerlifters with at least 6 months training experience | Non-para | To provide normative data on performance and selected anthropometric variables in adult powerlifters, determine possible relationships between body dimensions and performance, and develop equations that predict single lifts and overall performance | Subjective measures of powerlifting experience; anthropometric measurements; squat, bench press, and deadlift Wilks score | Stronger males had a significantly larger neck, and flexed and relaxed upper-arm and thigh girths compared to weaker male athletes; stronger females had significantly larger flexed and relaxed upper-arm and chest girths compared to weaker females |
| Muscle fiber characteristics of competitive power lifters (Fry et al., 2003) | 5 national level male powerlifters (31.0 ± 1.5 yrs; 101.8 ± 11.7 kg), 5 recreationally active male controls (27.3 ± 3.3 yrs; 85.2 ± 6.1 kg) | Non-para | To identify the cellular and molecular characteristics of skeletal muscle in national- and international-level powerlifters compared to sedentary controls, and to determine if these fibre characteristics are related to isokinetic squat force and power | Anthropometric measurements; vertical jump height; isokinetic squat peak force and power; fibre-type distribution; percent fibre-type area; myosin heavy chain analysis | The muscle characteristics contributing most to successful powerlifting performance appear to be greater percentage of IIA fibres, percentage area of IIA fibres, and percentage area of IIB fibres |
| Range of motion adaptations in powerlifters (Gadomski et al., 2018) | 15 male powerlifters (35.3 ± 13.7 yrs; 98.8 ± 19.1 kg), 15 age-matched controls with <6 months of weight training experience (34.9 ± 9.8 yrs; 96.7 ± 19.1 kg) | Non-para | To evaluate upper- and lower-extremity passive range of motion in powerlifters using goniometric analysis at the glenohumeral, hip, knee, and ankle joints | Subjective measures of training practices; Wilks score; passive glenohumeral, hip, knee, and ankle joint ranges of motion; Apley scratch test and modified Thomas test for multi-joint range of motion | Passive glenohumeral extension, internal rotation, and external rotation range of motion were significantly decreased in powerlifters; powerlifters displayed decreased range of motion in the Apley scratch test in both arms; knee extension angle was greater in powerlifters |
| Neuromuscular, anaerobic, and aerobic performance characteristics of elite power athletes (Haekkinen et al., 1984) | 4 national level male powerlifters (25.5 ± 5.0 yrs; 89.4 ± 7.3 kg), 7 national level male bodybuilders (25.6 ± 5.2 yrs; 87.6 ± 6.3 kg), 3 national level male wrestlers (24.0 ± 2.0 yrs; 77.5 ± 17.0 kg) | Non-para | To examine the influence of specific long-term exercise stress on various aspects of neuromuscular, anaerobic, and aerobic performances, and of histochemical muscle fibre characteristics in powerlifters, bodybuilders, and wrestlers | Neuromuscular performance; anaerobic performance; aerobic performance; anthropometric measurements; muscle fibre characteristics | Isometric force production was faster for the wrestlers and bodybuilders compared to the powerlifters; maximum oxygen consumption was higher for wrestlers and bodybuilders; no differences were observed in fibre distribution |
| Anthropometric characteristics of Malaysian competitive powerlifters with physical disabilities (Hamid et al., 2019) | 43 male (median 23 yrs; median 66.60 kg) and 9 female (median 28 yrs; median 66.10 kg) state, national, and international level Paralympic powerlifters | Para | To determine the sociodemographic and anthropometric parameters of Paralympic powerlifters in Malaysia | Subjective measures of sociodemographic information, participation history, and medical information; anthropometric measurements; body composition | There was a significant correlation between powerlifting performance and athletes’ age, years of experience, and several anthropometric parameters |
| Endocrine profiles in 693 elite athletes in the postcompetition setting (Healy et al., 2014) | 18 male national or international level powerlifters; 34.9 ± 5.3 yrs; 84.0 ± 19.5 kg | Non-para | To report on the hormonal profile of 693 elite athletes, sampled within 2 hours of a national or international  competitive event | Endocrine profile | 16.5% of men had low testosterone levels, whereas 13.7% of women had high levels with complete overlap between the sexes |
| Comparison of muscle strength imbalance in powerlifters and jumpers (Hui-Ying et al., 2014) | 11 male powerlifters (25 ± 3.29 yrs; 85.08 ± 16.25 kg), 8 male collegiate field jumpers (19.38 ± 1.41 yrs; 73.95 ± 6.32 kg) | Non-para | To determine if a force imbalance exists in dominant and nondominant legs between unilateral predominant and bilateral predominant sports, and to investigate the source of the force imbalance | Subjective measures of training history; body composition; double- and single-leg jump; countermovement vertical jump | The difference in force production between dominant and nondominant limbs is greater in jumpers than in powerlifters; powerlifters produced more force during double leg jumps, while jumpers produced more force during single leg jumps |
| Functional dimorphism and characteristics considering maximal hand grip force in top level athletes in the Republic of Serbia (Ivanović et al., 2009) | 25 powerlifters and 240 other athletes; 22.91 ± 2.87 yrs; 86.33 ± 2.87 kg | Non-para | To define functional dimorphism and basic model characteristics at maximum isometric handgrip force in both hands in top level athletes in the Republic of Serbia | Maximum isometric handgrip force | The highest average maximum isometric handgrip force value for left and right hand is found in powerlifters and the lowest in taekwondo |
| To what extent does sexual dimorphism exist in competitive powerlifters? (Keogh et al., 2008) | 54 male (36.2 ± 12.2 yrs; 94.2 ± 21.7 kg) and 14 female (40.4 ± 13.3 yrs; 65.1 ± 17.3 kg) national or international level New Zealand powerlifters | Non-para | To examine how the absolute and proportional anthropometric profile of successful competitive female and male powerlifters may differ | Anthropometric measurements | Competitive powerlifters exhibit sexual dimorphism for many absolute anthropometric measures like girths, but little dimorphism is found for measures of adiposity and for proportional segment lengths and bone breadths |
| Anthropometric dimensions of male powerlifters of varying body mass (Keogh et al., 2007) | 54 Oceania competitive male powerlifters; lightweight: 35.4 ± 15.0 yrs, 68.9 ± 7.9 kg, middleweight: 37.9 ± 12.8 yrs, 87.7 ± 6.9 kg, heavyweight: 33.4 ± 9.1 yrs, 121.9 ± 17.2 kg | Non-para | To examine how the anthropometry (in particular, the proportionality) of competitive male powerlifters may differ as a function of body mass | Anthropometric measurements | Heavyweights were significantly heavier, had greater muscle and fat mass, were more endo-mesomorphic, and had larger girths and bony breadths than the lighter lifters |
| Can absolute and proportional anthropometric characteristics distinguish stronger and weaker powerlifters? (Keogh et al., 2009) | 17 regional, national, or international level weaker (36.9 ± 15.2 yrs, 88.7 ± 13.9 kg) and 17 stronger (32.8 ± 7.4 yrs, 94.9 ± 23.7 kg) Oceania competitive male powerlifters | Non-para | To compare the anthropometric profile of successful and less successful competitive powerlifters | Anthropometric measurements | Stronger lifters had significantly greater muscle mass and larger muscular girths in absolute terms as well as greater Brugsch Index (chest girth/height) |
| Prediction of 1 repetition maximum in high-school power lifters (Kravitz et al., 2003) | 18 male state level high school powerlifters; 16.3 ± 1.2 yrs; 82.4 ± 21.9 kg | Non-para; high school | To develop prediction equations based on submaximal repetitions that best predict 1RM in squat, bench press, and deadlift exercises for high school powerlifters, and to determine which structural dimension variables best predict 1RM strength for this population | Anthropometric measurements; squat, bench press, and deadlift 1RM; maximum number of repetitions performed at 70%, 80%, and 90% of 1RM for each lift | Estimated 1RM derived from the regression equations using number of repetitions and number of repetitions x repetition weight at the submaximal %1RM as variables in the estimation equation was adequate for estimating 1RM strength for all lifts; no bodily structural dimension variable had a significant correlation with 1RM strength |
| Morphological features of the physical structure of powerlifters of different age and level of sports qualification (Kucherenko, 2020) | 8 novice (17-22 yrs), 8 unqualified (22-42 yrs), and 16 qualified (16-51 yrs) male powerlifters | Non-para | To identify and compare the characteristics of physical development of people engaged in powerlifting, depending on age and level of sportsmanship | Anthropometric measurements; vital capacity of lungs; strength of palm muscles; strength of back muscles; Body Mass Index; Erisman Index; Manouvrier Index; Body Proportionality Index; Pinye Index; Life Index | Differences are present in the physical profiles of skilled powerlifters compared to novices; powerlifting has minimal effect on aerobic capacity; adipose tissue levels were optimal, suggesting differences in physical development were due to variations in bone and muscle mass |
| Anthropometric profile of powerlifters: Differences as a function of bodyweight class and competitive success (Lovera & Keogh, 2015) | 63 male Argentine National Tournament powerlifters | Non-para | To describe the anthropometric characteristics of each male weight class in the Powerlifting Argentine National Tournament population, and to compare the winners of each bodyweight class to all other competitors in their class | Anthropometric measurements; competition results | While powerlifters have unique anthropometric profiles, more successful powerlifters typically have higher degrees of muscle mass, but similar segment lengths and segment length ratios to their less successful peers |
| Isokinetic strength of shoulder rotator muscles in powerlifters: Correlation between isometric and concentric muscle actions (Lucena et al., 2022) | 14 Brazilian powerlifters (28.9 ± 7.2 yrs; 83.9 ± 13.8 kg) and 9 physically active individuals (26.3 ± 7.3 yrs; 83.2 ± 15.5 kg) | Non-para; tested | To assess the shoulder rotators strength in powerlifters based on isokinetic dynamometer, isometric and concentric peak torque, and the isometric and concentric agonist/antagonist ratio | Absolute isometric and concentric peak torque; agonist/antagonist ratio | Isometric shoulder rotators strength was higher in powerlifters than physically active individuals; the bilateral differences in isometric external rotation in powerlifters suggest that exercises for shoulder rotators must be included in training for joint safety and balance between sides |
| Moderate-intensity strength exercise to exhaustion results in more pronounced signaling changes in skeletal muscles of strength-trained compared with untrained individuals (Lysenko et al., 2020) | 8 male powerlifters (median 29 yrs; median 92 kg); 8 recreationally active males (median 27 yrs; median 74 kg) | Non-para | To compare the response pattern of signalling proteins and genes regulating protein synthesis and degradation in skeletal muscle after strength exercise sessions performed to volitional fatigue in strength-trained and untrained males; to compare the expression of the signalling proteins and genes in trained and untrained muscle at rest | Medial quadriceps volume; blood hormone levels; vastus lateralis RNA, polymerase chain reaction, and myosin heavy chain contents, and western blot analysis | Moderate-intensity strength exercise performed to volitional fatigue changed the phosphorylation status of mTORC1 downstream signalling molecules and markers of ubiquitin-proteasome system activation in trained individuals, suggesting activation of protein synthesis and degradation; in contrast to the trained group, signalling responses in the untrained group were considerably less pronounced |
| Myosin heavy chain composition, creatine analogues, and the relationship of muscle creatine content and fast-twitch proportion to Wilks coefficient in powerlifters (Machek et al., 2020) | 6 male and 6 female competitive powerlifters (22.8 ± 3.7 yrs, 74.8 ± 15.9 kg), 5 male and 5 female sedentary controls | Non-para | To compare profiles of myosin heavy chain composition and creatine analogues between competitive powerlifters and controls of both sexes, and to determine whether specific targeted markers are predictors of powerlifting performance via Wilks coefficient | Myosin heavy chain fibre type; creatine analogue profiles | Neither myosin heavy chain IIA content nor muscle total creatine significantly predicted Wilks coefficient, suggesting these characteristics alone do not determine powerlifting skill variation |
| Assessment of training-associated changes of the lumbar back muscle using a multiparametric MRI protocol (Maggioni et al., 2024) | 11 male powerlifters; 26 ± 2 yrs | Non-para | To extract quantitative values of water, fat fraction, and Intra Voxel Incoherent Motion diffusion parameters in the lumbar back muscle | Relaxation parameters (water T_2_ and T_1_); fat fraction; Intra Voxel Incoherent Motion parameter values | The strength-trained athletes showed lower axial and higher radial diffusion components compared to the endurance-trained cohort, which may indicate muscle hypertrophy |
| Relationships of body dimensions to strength performance in novice adolescent male powerlifters (Mayhew et al., 1993) | 99 adolescent novice male powerlifters; 16.6 ± 1.0 yrs; 74.1 ± 16.5 kg | Non-para; adolescent | To determine the relationships between structural dimensions and strength performance among novice male high school athletes during a powerlifting competition | Anthropometric measurements; drop distance; pull distance; bench press and deadlift 1RM | Body mass was the most obvious factor contributing to the amount of weight lifted by adolescent athletes performing the bench press and deadlift; smaller athletes had greater relative strength performance |
| A comparison of strength and power characteristics between power lifters, Olympic lifters, and sprinters (McBride et al., 1999) | 8 national level powerlifters (24.1 ± 1.2 yrs; 78.2 ± 3.7 kg), 6 national level weightlifters, 6 national level sprinters, 8 controls | Non-para | To compare groups of athletes known to perform distinct styles of training in order to compare three protocols of exercise: high force, slow velocity; high force, high velocity; and low force, high velocity | Body composition; vertical jump performance; Smith machine squat 1RM; Smith machine squat jump biomechanics | The powerlifting group was significantly higher in peak force and peak power for jump trials at various loads in comparison to the control group |
| Characteristics of titin in strength and power athletes (McBride et al., 2003) | 5 powerlifters; 23.4 ± 1.0 yrs; 77.7 ± 4.8 kg | Non-para; tested | To see if any unique characteristics of titin could be identified in athletes with increased levels of strength or power | Smith machine squat 1RM, CMJ peak power; VL muscle biopsy: titin-1 %, titin-2 % | Titin protein band expression unique to the athletes coincided with superior performance in jumping and maximal dynamic strength; titin protein band expression patterns may correspond to patterns of muscular performance but cannot be confirmed by this study |
| Detection of exercise load-associated differences in hip muscles by texture analysis (Nketiah et al., 2015) | Female high-impact loading (triple jump: n = 9, high jump: n = 10), odd-impact loading (soccer: n = 9, squash: n = 10), high-magnitude loading (powerlifting: n = 17; 27.5 ± 6.3 yrs), low-impact loading (endurance running: n = 18), non-impact loading (swimming: n = 18), 20 nonathletes | Non-para | To identify whether there are significant differences in MRI-based hip muscle texture between athlete groups representing distinct sport-specific loading and a control group of habitually physically active nonathletes | Hip muscle MRI texture analysis | No significant difference in muscle texture was found between the high-magnitude (powerlifters) and the control group |
| Urinary incontinence in elite female powerlifters aged 20-30: Correlating musculoskeletal exam data with incontinence severity index and survey data (Nutt et al., 2024) | 31 female international level powerlifters; mean 24.32 yrs | Non-para | To correlate somatic dysfunctions identified during musculoskeletal screening with urinary incontinence severity, and to provide survey data representative of elite female powerlifters who experience urinary incontinence | Osteopathic musculoskeletal examination; subjective measures of urinary incontinence | Incontinence severity was found to be associated with certain somatic dysfunctions; the survey results display a positive relationship between age, history of pelvic floor examination, and engaging in leakage prevention practices with Incontinence Severity Index |
| The diagnostic value of attributes from an optimum set of variables for explaining an outcome in powerlifting at the junior level (Płóciennik et al., 2017) | 30 powerlifters; 19.38 ± 0.84 yrs | Non-para; junior | To find the optimum set of predictors and on that basis to build a biometric model of regression of young athletes’ sport result in powerlifting | Anthropometric measurements; biological maturity; aerobic and anaerobic capacity; muscle power; special physical fitness; technique; subjective measures of personality; reaction time | An optimum set of explanatory variables of the sport consists of 9 features; the axillary chest circumference at maximum inhalation has the highest information content on an outcome in powerlifting at the junior level |
| Body composition of male and female Chilean powerlifters of varying body mass (Palma-Lafourcade et al., 2019) | 40 male (30.3 ± 7.4 yrs; 84.9 ± 16.1 kg) and 16 female (27.4 ± 5.3 yrs; 56.5 ± 10.2 kg) national level Chilean powerlifters | Non-para; 47-120 kg weight class; open | To examine how the body composition of competitive male and female Chilean powerlifters may differ as a function of body mass and sex | Anthropometric measurements; squat, bench press, and deadlift 1RM | Heavier male lifters had significantly greater lean mass than lighter athletes; therefore, powerlifting performance was affected by anthropometric measures, as corroborated by 1RM scores; however, there was a general lack of differences in body composition between female weight classes, and, as a result, a lack of differences in 1RM performance |
| Relationships between body dimensions and strength abilities in experienced Olympic weightlifters, powerlifter and bodybuilders (Panayotov, 2020) | 14 national level bodybuilders (24.70 ± 4.97 yrs); 11 national level powerlifters (23.64 ± 3.14 yrs); 17 national level weightlifters (21.29 ± 2.97 yrs) | Non-para | To study differences in strength aspects of physical fitness and their relationships with body dimensions in competitive bodybuilders, weightlifters, and powerlifters | Anthropometric measurements; barbell back squat 1RM; barbell deadlift 1RM; standing bilateral long jump | Powerlifters were the strongest and weightlifters were the most explosive; strong relationships were found between body dimensions and strength in bodybuilders and weightlifters but not in powerlifters |
| Prediction of bench press performance in powerlifting: The role of upper limb anthropometry (Pasini et al., 2023) | 47 male Italian Powerlifting Federation powerlifters; 30.7 ± 8.8 yrs; 94.0 ± 19.2 kg | Non-para; raw | To evaluate which anthropometric and body composition variables are the most correlated with performance in bench press and whether the novel anthropometric indices can successfully predict 1RM bench press | Anthropometric measurements; bench press 1RM | The upper arm cross muscular area and fat-free mass were positively correlated with Wilks points, whereas the arm fat index was negatively correlated with 1RM bench press |
| A morphometric analysis of human muscle fibers with relation to fiber types and adaptations to exercise (Prince et al., 1981) | 2 international level powerlifters, 3 long distance runners, 9 active controls | Non-para | To examine the volume percent composition of central mitochondria and lipid and Z-line width of fibres from male subjects involved in different activities, to estimate the values of the above parameters for the specific fibre types, and characterise the adaptability of fibre types in response to endurance and strength activity | Vastus lateralis muscle fibre volume % mitochondria, volume % lipid, Z-line width | The high-strength anaerobic activity of the powerlifters was reflected by the low mitochondrial volume percent of many fast-twitch fibres and the decreased lipid stores in all fibres |
| Human muscle fiber types in power lifters, distance runners and untrained subjects (Prince et al., 1976) | 4 male powerlifters, 3 male distance runners, 5 male controls | Non-para | To attempt to determine the number of fibre types, based on ATPase and SDH activities, found in human muscle and compare the fibre types as well as cross-sectional areas in powerlifters, distance runners, and control subjects | Vastus lateralis muscle fibre type | The major characteristics of the lifters were a decrease in the percentage of fast oxidative glycolytic fibres and a hypertrophy of fast oxidative glycolytic and fast glycolytic fibres |
| Diagnostic sources of information on sports result determinants in young powerlifting athletes (Rygula et al., 2016) | 30 elite or sub-elite powerlifters; 19.4 ± 0.7 yrs; 83.4 ± 11.12 kg | Non-para | To find the optimal set of predictors and, on this basis, to build a biometric model of regression of young athletes’ sports result in powerlifting | Anthropometric measurements; maturity status; aerobic and anaerobic capacity; muscle power indices; special physical fitness; movement technique indices; subjective measures of personality; reaction time; haemodynamic parameters | The optimal set of variables predicting sports results in junior powerlifting consists of nine features; it was confirmed that body composition, special physical fitness, and the technique of movement would constitute the optimum combination of explanatory variables of the model |
| Influence of lumbopelvic stability on deadlift performance in competitive powerlifters (Sakakibara et al., 2014) | 8 national level male powerlifters (39.9 ± 8.8 yrs; 83.4 ± 27.3 kg), 8 regional level male powerlifters (34.6 ± 7.0 yrs; 74.4 ± 10.3 kg) | Non-para | To compare transversus abdominis muscle contractile rates between national and regional level powerlifters while they perform the deadlift using ultrasound imaging to investigate the influence of lumbopelvic stability on deadlift performance | Transversus abdominis muscle thickness and contractile rate | Compared with the control group, the elite powerlifters showed a higher transversus abdominis contractile rate when the weight was at knee level; there were no significant differences between the transversus abdominis contractile rates in both groups when the weight was at the floor and top level |
| Force capacity of trunk muscle extension and flexion in healthy inactive, endurance and strength-trained subjects—a pilot study (Schönau & Anders, 2024) | 13 powerlifters (23.5 ± 1.8 yrs; 90.3 ± 13.9 kg), 13 endurance trained athletes, 12 physically inactive people | Non-para | To compare the maximal strength capacity of trunk muscles between physically inactive individuals and ambitious recreational endurance and strength athletes | Trunk muscles maximum voluntary contraction and torque (isometric flexion and extension) | Powerlifters show higher functional force capacity values for flexion compared to the other groups; for extension, strength-trained and endurance-trained did not differ |
| Why do endocrine profiles in elite athletes differ between sports? (Sönksen et al., 2018) | 18 male national or international level powerlifters; 34.9 ± 5.3 yrs; 84.0 ± 19.5 kg | Non-para | To examine the differences in endocrine profiles discovered by Healy et al. in more detail and attempt to interpret some of the findings | Endocrine profile | The testosterone concentrations in the powerlifters are on average remarkably small and 8 of the remaining sports had significantly larger values |
| Range of motion is not reduced in national-level New Zealand female powerlifters (Spence et al., 2021) | 12 female national level New Zealand Powerlifting Federation powerlifters, 12 recreationally trained controls; 26.3 ± 6.6 yrs; 65.9 ± 9.9 kg | Non-para | To determine active single-joint range of motion in female powerlifters and to determine whether single-joint range of motion can be used to predict strength levels in female powerlifters | Shoulder, hip, and knee ranges of motion | Powerlifters had significantly greater shoulder horizontal abduction on the right side, but no other ranges were significantly different between groups, and no ranges were significantly related to strength |
| Range of motion predicts performance in national-level New Zealand male powerlifters (Spence et al., 2023) | 12 male national level New Zealander International Powerlifting Federation powerlifters (27.1 ± 4.5 yrs; 86.6 ± 16.3 kg); 12 recreationally strength trained controls (27.1 ± 5.1 yrs; 90.0 ± 10.1 kg) | Non-para; raw | To determine if shoulder, hip, and knee range of motion were different in male powerlifters compared with recreationally strength-trained men, and to see if there were any relationships between range of motion and strength or average eccentric velocity at submaximal loads | Squat and bench press 1RM; shoulder, hip, and knee ranges of motion; squat barbell average eccentric velocity | Powerlifters had significantly less shoulder extension and horizontal abduction, and hip flexion, extension, and adduction than recreationally strength-trained men; significant negative relationships were found between 2-lift Wilks score and shoulder extension and horizontal abduction, as well as hip flexion and extension; in powerlifters, significant negative relationships were found between competition Wilks score, shoulder extension, and hip flexion |
| The relationship between powerlifting performance and hand grip strength among female athletes (Suazo & DeBeliso, 2021) | 31 female powerlifters; 28.9 ± 5.5 yrs; 83.1 ± 28.2 kg | Non-para; raw | To investigate the relationship between hand grip strength and powerlifting performance among female athletes | Hand grip strength; squat, bench press, deadlift, and total results | Back squat, deadlift, and total scores had moderately significant relationships with hand grip strength; bench press performance had a low but significant association with hand grip strength; all relative scores had significant moderate-high relationships with hand grip strength |
| Locked and loaded: Divergent handgrip tests as surrogate measures for one-repetition maximal strength (Travis et al., 2025) | 22 male powerlifters; 22.55 ± 3.32 yrs; 94.35 ± 20.43 kg | Non-para; raw tested | To investigate hand grip strength as a potential surrogate measure for 1RM performances in key compound lifts via back squat, bench press, deadlift, and total, while accounting for variations in testing posture | Standing handgrip strength; seated handgrip strength | Seated handgrip strength was most closely associated with bench press 1RM, with a strong relationship with total |
| Lean body mass, muscle architecture and powerlifting performance during preseason and in competition (Tromaras et al., 2024) | 8 male (31.7 ± 9.8 yrs; 99.2 ± 14.6 kg) and 3 female (32.7 ± 16.3 yrs; 66.6 ± 20.9 kg) competitive powerlifters | Non-para | To investigate the relationship between training-induced changes in lean body mass and muscle architecture and the changes in performance in well-trained powerlifters preparing for a competition | Squat, bench press, and deadlift 1RM; body composition; quadriceps cross-sectional area; handgrip strength; countermovement jump performance; modified Wingate test performance | Significant increases were found after the training period in the squat, bench press, and deadlift; significant correlations were found between the 1RM and lean body mass before and after the training period; the changes in the 1RM after the training intervention correlated with the changes in the total lean body mass, suggesting that individual changes in lean body mass due to systematic resistance training for a competition may dictate increases in the 1RM strength in powerlifters |
| Urinary incontinence in competitive women powerlifters: A cross-sectional survey (Wikander et al., 2021) | 480 local, national, or international level female powerlifters; 35 ± 10 yrs; 74 ± 18 kg | Non-para | To determine if load, body position, and fatigue influenced the likelihood of urinary incontinence in competitive women powerlifters during training and competition; to determine the prevalence of urinary incontinence in competitive women powerlifters and the relationship between risk factors and their Incontinence Severity Index score; to determine participants’ confidence in performing a pelvic floor contraction; to identify activities that provoked urinary incontinence and the self-care practices they engaged in to manage leakage of urine during training and competition | Subjective measures of urinary incontinence experience | The prevalence of urinary incontinence in this cohort was at the upper limit experienced by women in the general population; women who had undergone a pelvic floor examination or were confident in correctly performing pelvic floor exercises experienced less severe urinary incontinence |

**References**

Androulakis-Korakakis, P., Gentil, P., Fisher, J. P., & Steele, J. (2021). Comparison of isolated lumbar extension strength in competitive and noncompetitive powerlifters, and recreationally trained men. *Journal of Strength and Conditioning Research*, *35*(3). <https://doi.org/10.1519/JSC.0000000000002722>

Brechue, W. F., & Abe, T. (2002). The role of FFM accumulation and skeletal muscle architecture in powerlifting performance. *European Journal of Applied Physiology*, *86*(4), 327-336. <https://doi.org/10.1007/s00421-001-0543-7>

Chandler, T. J., Wilson, G. D., & Stone, M. H. (1989). The effect of the squat exercise on knee stability. *Medicine and Science in Sports and Exercise*, *21*(3), 299-303.

Chang, D. E., Buschbacher, L. P., & Edlich, R. F. (1988). Limited joint mobility in power lifters. *American Journal of Sports Medicine*, *16*(3), 280-284. <https://doi.org/10.1177/036354658801600315>

Cutrufello, P. T., Gadomski, S. J., & Ratamess, N. A. (2017). An evaluation of agonist: Antagonist strength ratios and posture among powerlifters. *Journal of Strength and Conditioning Research*, *31*(2), 298-304. <https://doi.org/10.1519/JSC.0000000000001724>

Di Naso, J. J., Pritschet, B. L., Emmett, J. D., Owen, J. D., Willardson, J. M., Beck, T. W., DeFreitas, J. M., & Fontana, F. E. (2013). Comparing thigh muscle cross-sectional area and squat strength among national class Olympic weightlifters, power lifters, and bodybuilders. *International SportMed Journal*, *14*(1), 1-10.

Ereline, J., Gapeyeva, H., & Päsuke, M. (2004). Contractile changes in knee extensor muscles after repetitive maximal isokinetic contractions in male power-lifters and untrained subjects. *Medicina dello Sport*, *57*(1), 29-39.

Ferland, P.-M., Laurier, A., & Comtois, A. S. (2020a). Relationships between anthropometry and maximal strength in male classic powerlifters. *International Journal of Exercise Science*, *13*(4), 1512-1531.

Ferland, P.-M., St-Jean Miron, F., Laurier, A., & Comtois, A. S. (2020b). The relationship between body composition measured by dual-energy X-ray absorptiometry and maximal strength in classic powerlifting. *Journal of Sports Medicine and Physical Fitness*, *60*(3), 407-416. <https://doi.org/10.23736/S0022-4707.19.09996-1>

Ferrari, L., Colosio, A. L., Teso, M., & Pogliaghi, S. (2022). Performance and anthropometrics of classic powerlifters: Which characteristics matter? *Journal of Strength and Conditioning Research*, *36*(4), 1003-1010. <https://doi.org/10.1519/JSC.0000000000003570>

Fry, A. C., Webber, J. M., Weiss, L. W., Harber, M. P., Vaczi, M., & Pattison, N. A. (2003). Muscle fiber characteristics of competitive power lifters. *Journal of Strength and Conditioning Research*, *17*(2), 402-410.

Gadomski, S. J., Ratamess, N. A., & Cutrufello, P. T. (2018). Range of motion adaptations in powerlifters. *Journal of Strength and Conditioning Research*, *32*(11), 3020-3028. <https://doi.org/10.1519/JSC.0000000000002824>

Haekkinen, K., Alen, M., & Komi, P. V. (1984). Neuromuscular, anaerobic, and aerobic performance characteristics of elite power athletes. *European Journal of Applied Physiology and Occupational Physiology*, *53*(2), 97-105. <https://doi.org/10.1007/BF00422570>

Hamid, M. S. A., Shariff-Ghazali, S., & Abdul Karim, S. (2019). Anthropometric characteristics of Malaysian competitive powerlifters with physical disabilities. *Journal of Health and Translational Medicine*, *22*(2), 49-55. <https://doi.org/10.22452/jummec.vol22no2.8>

Healy, M.-L., Gibney, J., Pentecost, C., Wheeler, M. J., & Sonksen, P. H. (2014). Endocrine profiles in 693 elite athletes in the postcompetition setting. *Clinical Endocrinology*, *81*(2), 294-305. <https://doi.org/10.1111/cen.12445>

Hui-Ying, L., Winter, C., O'Neill, E., & Thompson, B. A. (2014). Comparison of muscle strength imbalance in powerlifters and jumpers. *Journal of Strength and Conditioning Research*, *28*(1), 23-27. <https://doi.org/10.1519/jsc.0b013e318295d311>

Ivanović, J., Koropanovski, N., Vučković, G., Janković, R., Miljuš, D., Marinković, B., Atanasov, D., Blagojević, M., & Dopsaj, M. (2009). Functional dimorphism and characteristics considering maximal hand grip force in top level athletes in the Republic of Serbia. *Gazzetta Medica Italiana Archivio per le Scienze Mediche*, *168*(5), 297-310.

Keogh, J. L., Hume, P., Pearson, S., & Mellow, P. (2008). To what extent does sexual dimorphism exist in competitive powerlifters? *Journal of Sports Sciences*, *26*(5), 531-541. <https://doi.org/10.1080/02640410701644034>

Keogh, J. W., Hume, P. A., Pearson, S. N., & Mellow, P. (2007). Anthropometric dimensions of male powerlifters of varying body mass. *Journal of Sports Sciences*, *25*(12), 1365-1376. <https://doi.org/10.1080/02640410601059630>

Keogh, J. W. L., Hume, P. A., Pearson, S. N., & Mellow, P. J. (2009). Can absolute and proportional anthropometric characteristics distinguish stronger and weaker powerlifters? *Journal of Strength and Conditioning Research*, *23*(8), 2256-2265. <https://doi.org/10.1519/JSC.0b013e3181b8d67a>

Kravitz, L., Akalan, C., Nowicki, K., & Kinzey, S. J. (2003). Prediction of 1 repetition maximum in high-school power lifters. *Journal of Strength and Conditioning Research*, *17*(1), 167-172.

Kucherenko, G. (2020). Morphological features of the physical structure of powerlifters of different age and level of sports qualification. *Slobozhanskyi Herald of Science and Sport*, *2020*(5), 38-44. <https://doi.org/10.15391/snsv.2020-5.006>

Lovera, M., & Keogh, J. (2015). Anthropometric profile of powerlifters: Differences as a function of bodyweight class and competitive success. *Journal of Sports Medicine and Physical Fitness*, *55*(5), 478-487.

Lucena, E. G., Ferland, P.-M., Ahmadi, S., Teixeira, L. F., Comtois, A. S., & Uchida, M. C. (2022). Isokinetic strength of shoulder rotator muscles in powerlifters: Correlation between isometric and concentric muscle actions. *Journal of Sports Medicine and Physical Fitness*, *62*, 170-176. <https://doi.org/10.23736/S0022-4707.21.11921-8>

Lysenko, E. A., Popov, D. V., Vepkhvadze, T. F., Sharova, A. P., & Vinogradova, O. L. (2020). Moderate-intensity strength exercise to exhaustion results in more pronounced signaling changes in skeletal muscles of strength-trained compared with untrained individuals. *Journal of Strength and Conditioning Research*, *34*(4). <https://doi.org/10.1519/JSC.0000000000002901>

Machek, S. B., Hwang, P. S., Cardaci, T. D., Wilburn, D. T., Bagley, J. R., Blake, D. T., Galpin, A. J., & Willoughby, D. S. (2020). Myosin heavy chain composition, creatine analogues, and the relationship of muscle creatine content and fast-twitch proportion to Wilks coefficient in powerlifters. *Journal of Strength and Conditioning Research*, *34*(11), 3022-3030. <https://doi.org/10.1519/jsc.0000000000003804>

Maggioni, M. B., Sibgatulin, R., Krämer, M., Güllmar, D., & Reichenbach, J. R. (2024). Assessment of training-associated changes of the lumbar back muscle using a multiparametric MRI protocol. *Frontiers in Physiology*, *15*, 1408244. <https://doi.org/10.3389/fphys.2024.1408244>

Mayhew, J. L., McCormick, T. P., Piper, F. C., Kurth, A. L., & Arnold, M. D. (1993). Relationships of body dimensions to strength performance in novice adolescent male powerlifters. *Pediatric Exercise Science*, *5*(4), 347-356. <https://doi.org/10.1123/pes.5.4.347>

McBride, J. M., Triplett-McBride, T., Davie, A., & Newton, R. U. (1999). A comparison of strength and power characteristics between power lifters, Olympic lifters, and sprinters. *Journal of Strength and Conditioning Research*, *13*(1), 58-66.

McBride, J. M., Triplett-McBride, T., Davie, A. J., Abernethy, P. J., & Newton, R. U. (2003). Characteristics of titin in strength and power athletes. *European Journal of Applied Physiology*, *88*(6), 553-557. <https://doi.org/10.1007/s00421-002-0733-y>

Nketiah, G., Savio, S., Dastidar, P., Nikander, R., Eskola, H., & Sievänen, H. (2015). Detection of exercise load-associated differences in hip muscles by texture analysis. *Scandinavian Journal of Medicine and Science in Sports*, *25*(3), 428-434. <https://doi.org/10.1111/sms.12247>

Nutt, K. D., Carnes, M., Griffin, L., & Rivin, G. (2024). Urinary incontinence in elite female powerlifters aged 20-30: Correlating musculoskeletal exam data with incontinence severity index and survey data. *Journal of Osteopathic Medicine*, *125*(7), 341-349. <https://doi.org/10.1515/jom-2024-0079>

Palma-Lafourcade, P., Cisterna, D., Hernandez, J., Ramirez-Campillo, R., Alvarez, C., & Keogh, J. W. (2019). Body composition of male and female Chilean powerlifters of varying body mass. *Motriz. Revista de Educacao Fisica*, *25*(1). <https://doi.org/10.1590/s1980-6574201900010018>

Panayotov, V. (2020). Relationships between body dimensions and strength abilities in experienced Oympic weightlifters, powerlifters and bodybuilders. *Series on Biomechanics*, *34*(4), 52-58.

Pasini, A., Caruso, L., Bortolotto, E., Lamberti, N., Toselli, S., Manfredini, F., Zaccagni, L., & Rinaldo, N. (2023). Prediction of bench press performance in powerlifting: The role of upper limb anthropometry. *Journal of Human Sport and Exercise*, *18*(2), 484-500. <https://doi.org/10.14198/jhse.2023.182.18>

Płóciennik, Ł., Ryguła, I., Dancewicz, T., & Łukaszuk, K. (2017). The diagnostic value of attributes from an optimum set of variables for explaining an outcome in powerlifting at the junior level. *Baltic Journal of Health and Physical Activity*, *9*(4), 32-43. <https://doi.org/10.29359/BJHPA.09.4.03>

Prince, F. P., Hikida, R. S., & Hagerman, F. C. (1976). Human muscle fiber types in power lifters, distance runners and untrained subjects. *Pflugers Archiv European Journal of Physiology*, *363*(1), 19-26. <https://doi.org/10.1007/BF00587397>

Prince, F. P., Hikida, R. S., Hagerman, F. C., Staron, R. S., & Allen, W. H. (1981). A morphometric analysis of human muscle fibers with relation to fiber types and adaptations to exercise. *Journal of the Neurological Sciences*, *49*(2), 165-179. <https://doi.org/10.1016/0022-510x(81)90076-9>

Rygula, I., Plociennik, L., & Lipinska, P. (2016). Diagnostic sources of information on sports result determinants in young powerlifting athletes. *Human Movement*, *17*(3), 168-175. <https://doi.org/10.1515/humo-2016-0027>

Sakakibara, N., Shin, S., Watanabe, T., & Matsuoka, T. (2014). Influence of lumbopelvic stability on deadlift performance in competitive powerlifters. *SportLogia*, *10*(2), 89-95. <https://doi.org/10.5550/sgia.141002.en.005S>

Schönau, T., & Anders, C. (2024). Force capacity of trunk muscle extension and flexion in healthy inactive, endurance and strength-trained subjects—a pilot study. *German Journal of Exercise and Sport Research*, *54*(2), 213-220. <https://doi.org/10.1007/s12662-023-00904-8>

Sönksen, P. H., Holt, R. I. G., Böhning, W., Guha, N., Cowan, D. A., Bartlett, C., & Böhning, D. (2018). Why do endocrine profiles in elite athletes differ between sports? *Clinical Diabetes and Endocrinology*, *4*, 1-16. <https://doi.org/10.1186/s40842-017-0050-3>

Spence, A.-J., Helms, E. R., & McGuigan, M. R. (2021). Range of motion is not reduced in national-level New Zealand female powerlifters. *Journal of Strength and Conditioning Research*, *35*(10), 2737-2741. <https://doi.org/10.1519/jsc.0000000000004117>

Spence, A.-J., Helms, E. R., Sousa, C. A., & McGuigan, M. R. (2023). Range of motion predicts performance in national-level New Zealand male powerlifters. *Journal of Strength and Conditioning Research*, *37*(1), 123-128. <https://doi.org/10.1519/jsc.0000000000004205>

Suazo, N., & DeBeliso, M. (2021). The relationship between powerlifting performance and hand grip strength among female athletes. *Turkish Journal of Kinesiology*, *7*(4), 112-122.

Travis, S. K., Schwarz, A. V., & Burke, B. I. (2025). Locked and loaded: Divergent handgrip tests as surrogate measures for one-repetition maximal strength. *Biomechanics*, *5*(1), Article 16. <https://doi.org/10.3390/biomechanics5010016>

Tromaras, K., Zaras, N., Stasinaki, A.-N., Mpampoulis, T., & Terzis, G. (2024). Lean body mass, muscle architecture and powerlifting performance during preseason and in competition. *Journal of Functional Morphology and Kinesiology*, *9*(2), 89. <https://doi.org/10.3390/jfmk9020089>

Wikander, L., Kirshbaum, M. N., Waheed, N., & Gahreman, D. E. (2021). Urinary incontinence in competitive women powerlifters: A cross-sectional survey. *Sports Medicine - Open*, *7*(1), 1-11. <https://doi.org/10.1186/s40798-021-00387-7>
