# Supplementary material for: The Applied Sport Science and Medicine of Powerlifting and Para Powerlifting: A Systematic Scoping Review with Recommendations for Future Research
Source: Sports Med. 2025 Sep 9;55(11):2849–77. doi: 10.1007/s40279-025-02305-3 (PMC12559058; doi:10.1007/s40279-025-02305-3)
Supplement: Supplementary file 5 — Supplementary file5 (DOCX 35 KB) [file 40279_2025_2305_MOESM5_ESM.docx]

**Supplementary Table S5.** Characteristics, outcomes measures, and key findings of ‘injury’ studies (n = 18)

| **Study** | **Cohort and sample size (n); age; body mass (where specified)** | **Competitive characteristics: para status; division; weight class; age category (where specified)** | **Study aim(s)** | **Outcome measures** | **Key findings** |
| --- | --- | --- | --- | --- | --- |
| A descriptive analysis of functional impairments and patho-anatomical findings in eight powerlifters (Aasa & Berglund, 2020) | 8 Swedish Powerlifting Federation powerlifters; 35-48 yrs | Non-para; tested; 59-120+ kg | To describe functional impairments (physical and psychological) and patho-anatomical findings in powerlifters with and without low back pain | Questionnaires; anthropometry; patho-anatomical findings; physical performance testing | The powerlifters with and without lower back pain showed similar functional impairments and patho-anatomical findings, but the pain-free controls tended to have a more ideal squatting technique |
| Medical history associated with adolescent powerlifting (Brown & Kimball, 1983) | 71 male powerlifters; 14-19 yrs | Non-para; all weight classes; teenage category | To construct a profile of the medical history of adolescent powerlifters in order to comprehend more fully the potential for injury in the sport | Subjective measures of training characteristics and medical history | The low back region was the site with the greatest number of injuries |
| Pain and injury associated with powerlifting training in visually impaired athletes (Haykowsky et al., 1999) | 9 male and 2 female Canadian Blind Sports Association National Powerlifting Championships powerlifters; 22-75 yrs (37 yrs average) | Non-para | To assess the occurrence and perceived level of pain and injury history associated with powerlifting training in visually impaired athletes. | Perceived level of pain and injuries | Powerlifting training as performed by visually impaired athletes is associated with an elevated occurrence of pain in the shoulder, elbow, lower back, and knee regions that is subjectively rated as "mild to severe" |
| Sports injuries at the Rio de Janeiro 2016 Summer Paralympic Games: Use of diagnostic imaging services (Jarraya et al., 2021) | 20 Paralympic powerlifters (plus athletes of other sports) | Para; tested | To describe the frequencies of sports-related bone, muscle, tendon, and ligament injuries in para-athletes that underwent imaging and to document the use of imaging with radiography, ultrasound, and MRI during the Rio 2016 Summer Paralympic Games | Imaging utilisation and findings | The highest imaging utilisation rates were found among para-athletes competing in Judo, sitting volleyball, powerlifting, and football; the disciplines most prone to muscle injuries were athletics and powerlifting; tendon injuries were most commonly found in powerlifting; powerlifting injuries affected the upper limbs only |
| Retrospective injury epidemiology of one hundred one competitive Oceania power lifters: The effects of age, body mass, competitive standard, and gender (Keogh et al., 2006) | 82 male and 19 female competitive powerlifters; 36.6 ± 12.4 yrs; 88 ± 24 kg | Non-para; tested | To investigate the injury epidemiology of competitive powerlifters | Subjective measures of anthropometric, demographic, training, and injury information | The most commonly injured body regions were the shoulder, lower back, elbow, and knee; injuries tended to be acute rather than chronic |
| Lumbopelvic movement control in powerlifters with and without low back pain (Larsson et al., 2024) | 12 powerlifters with (27.5 ± 4.8 yrs; 84.8 ± 15.0 kg) and 28 powerlifters without (25.2 ± 4.3 yrs; 84.9 ± 15.5 kg) lower back pain | Non-para | To investigate whether there is a difference between powerlifters with lower back pain and powerlifters without lower back pain in a test battery score for movement control of the lumbopelvic region and in individual movement control tests | Lumbopelvic movement control; subjective measures of training and injury history | The lack of significant differences between groups indicates that performance in lumbopelvic movement control tests might not be associated with lower back pain in powerlifters |
| Role of the flexion relaxation phenomenon in the analysis of low back pain risk in the powerlifter: a proof-of-principle study (Marotta et al., 2024) | 10 powerlifters without lower back pain history (28.4 ± 12.1 yrs; 86.00 ± 5.05 kg) and 8 powerlifters with lower back pain history (27.4 ± 3.1 yrs; 86.56 ± 6.85 kg) | Non-para | To evaluate the risk of lower back pain in powerlifting athletes through the electromyographic analysis of the flexion relaxation phenomenon | Bench press arch; flexion relaxation ratio; EMG signal | Flexion relaxation ratio could be considered as a useful parameter for studying the risk of lower back pain in powerlifting; the size of the arch may not be a determining factor in the occurrence of lower back pain |
| Development of a comprehensive clinical assessment protocol for low back and hip pain in powerlifters: A feasibility study (Olofsson et al., 2024) | 5 male (mean 24 yrs, 93 kg) and 3 female (mean 26 years, 66 kg) powerlifters | Non-para | To develop a comprehensive clinical assessment protocol for pain and function in powerlifters with low back and/or hip pain and evaluate its feasibility for use in further studies aiming to describe prevalence of different pain problems/injury types, evaluate treatments, or investigate the risk of injuries in powerlifting | Subjective and physical examination | This study showed that it was feasible to use the novel comprehensive clinical assessment protocol to formulate a clinical diagnosis of low back and/or hip pain in powerlifters including neurophysiological pain mechanisms and impairments in body functions |
| Injury epidemiology and preparedness in powerlifting at the Rio 2016 Paralympic Games: An analysis of 1410 athlete-days (Ona Ayala et al., 2019) | 180 Paralympic powerlifters | Para | To quantify the injury incidence rate and injury incidence proportion among Para powerlifters during the 3-day pre-competition and 7-day competition period at Rio 2016 Summer Paralympic Games | Subjective measures of injury incidence | Most injuries were from chronic overuse; the most commonly injured anatomical region was the shoulder; there were no significant differences in injury patterns between males and females, and the highest injury incidence rate was found in the oldest age group; there was no difference between lighter and heavier athletes |
| Prevalence and incidence of health problems and their characteristics in Brazilian para athletes: a one-season single-center prospective pilot study (Pinheiro et al., 2024) | 10 male and 2 female Paralympic powerlifters (36.2 ± 8.6 yrs; 78.4 ± 20.2 kg), 23 other para athletes | Para | To describe the characteristics of injuries and illnesses and establish the prevalence, incidence, and severity of health problems of para athletics, para powerlifting, and para swimming athletes of one of the Brazilian Paralympic Reference Centres during a sports season | Subjective measures of health problems | Para powerlifting had the highest prevalence of all and substantial health problems |
| Injury incidence and prevalence among elite weight and power lifters (Raske & Norlin, 2002) | 1995: 50 male and 5 female powerlifters, 50 male and 5 female weightlifters, 50 non-elite weightlifting controls, 2000: 50 male and 10 female powerlifters, 50 male and 5 female weightlifters | Non-para | To investigate the incidence and prevalence of injuries among elite Swedish weightlifters and powerlifters, with a special focus on shoulder injuries and possible injury-provoking exercises | Subjective measures of injury incidence | In 1995 and again in 2000, the athletes sustained, on average, 2.6 injuries per 1000 hours of activity; most common in 1995 were low back injuries, with an injury rate of 0.43 per 1000 hours, and shoulder injuries, with a rate of 0.42 per 1000 hours; shoulder injuries dominated in 2000, with an injury rate of 0.51 per 1000 hours of activity; there was a difference in injury pattern between weightlifters, who mostly sustained low back and knee injuries, and powerlifters, in whom shoulder injuries were most common; no correlation was found between shoulder injuries and any specific exercise |
| Incidence and characteristics of acute and overuse injuries in elite powerlifters (Reichel et al., 2019) | 54 male and 3 female powerlifters; 33.4 ± 11.2 yrs; 96.7 ± 23.5 kg | Non-para; tested | To examine possible relations between sport-specific equipment in competitive  powerlifting and resulting acute or chronic overuse injuries, and to examine the influence of different preventive and regenerative strategies like stretching, warming up, sauna,  training intensity, and training volume on injury incidence | Subjective measures of training practices and injury history | With 224 total injuries, a mean incidence of 1.51 per 1000 hours or 0.49 per year was calculated; most injuries affected the lower back (20.5%), elbow (11.2%), pelvic  region (10.3%), and the shoulder (9.8%) |
| Comprehensive 1-year multilevel study of sports injuries in para athletes: Impact of season timing, years of sports experience, impairment and sports type (Resende et al., 2025) | 11 male and 3 female Paralympic powerlifters (mean 32.5 yrs, 63.9 kg), 88 other para athletes | Para | To investigate the longitudinal associations between season timing, years of sports experience, impairment type, and sport type with injury occurrence and time loss over one year | Subjective measures of health problems | Para powerlifters had 66% lower odds of experiencing sudden-onset injuries compared with athletes in para athletics, possibly due to the more predictable nature of para powerlifting training |
| Effect of the athletes’ origin and type of impairment on participation and the likelihood of winning a medal in the Paralympic games (Severin et al., 2025) | 21235 para athletes’ results (powerlifting sample size NR) | Para | To chart the proportions of athletes with each origin and type of impairment that participates in the different sports, and to examine if the likelihood of winning a medal is higher in athletes with a given impairment | Sport; podium results; origin and type of impairment | Athletes with an acquired spinal cord injury win proportionally fewer medals in para powerlifting |
| Injuries and overuse syndromes in powerlifting (Siewe et al., 2011) | 219 male and 26 female competitive and elite powerlifters; 37.8 ± 14.3 yrs; 89.1 ± 17.1 kg | Non-para | To identify problem zones during workouts, rates of injury, as well as interacting factors, to offer advice to both athletes and sports medicine practitioners for injury prevention and accelerated rehabilitation in the sport of powerlifting | Subjective measures of training practices and pain and injury history | 43.3% of powerlifters complained of problems during routine workouts; injury rate was calculated as 0.3 injuries per lifter per year; most commonly injured body regions were the shoulder, lower back and the knee; the use of weight belts increased the injury rate of the lumbar spine; rate of injury to the upper extremities was significantly increased based on age > 40 years and female gender |
| Content validity index and reliability of a new protocol for evaluation of lifting technique in the powerlifting squat and deadlift (Sjöberg et al., 2020) | 8 powerlifting experts (researchers, coaches, and competitors), 30 male and 10 female regional, national, or international level powerlifters; powerlifters = 18-61 yrs | Non-para; 57-120+ kg weight class | To examine aspects of squatting and deadlifting technique considered to be associated with risk of injury and to investigate the content validity and reliability of these aspects | Injury risk test battery results during squats and deadlifts | The final protocols included 17 aspects of squat technique and 10 aspects of deadlift technique that showed good to excellent Content Validity Index and percentage of agreement between 64 and 100% |
| Prevalence and consequences of injuries in powerlifting (Strömbäck et al., 2018) | 51 male and 53 female Swedish Powerlifting Federation powerlifters; 28.3 ± 7.6 yrs; 79.9 ± 17.6 kg | Non-para; classic; open category | To investigate the prevalence, localisation, and characterisation of injuries among Swedish sub elite classic powerlifters, with an emphasis on differences between men and women, and to investigate whether training and lifestyle factors are associated with an injury | Subjective measures of competition experience, training practices, injury history, and lifestyle characteristics | Injuries are very common in sub elite powerlifters; men and women report similar injury frequencies but different anatomic locations; these injuries do not prevent powerlifters from training and competing, but they may change the content of training sessions; why powerlifters develop injuries is still unclear; however, it is likely that the management of training loads and optimisation of the lifting technique during the squat, bench press, and deadlift are of importance |
| The epidemiology of injuries in powerlifting at the London 2012 Paralympic Games: An analysis of 1411 athlete-days (Willick et al., 2016) | 98 male and 65 female London 2012 Paralympic Games powerlifters | Para; tested | To describe the incidence, types, and risk factors for injury in powerlifters during the London 2012 Paralympic Games | Injury incidence rate; injury incidence proportion | 38 injuries were powerlifters; the overall injury incidence rate was 33.3 injuries per 1000 athlete-days and the overall injury incidence proportion was 23.3 injuries per 100 athletes; the majority of injuries were chronic overuse injuries; the most commonly injured anatomical region was the shoulder/clavicle, followed by the chest and elbow |

**References**

Aasa, U., & Berglund, L. (2020). A descriptive analysis of functional impairments and patho-anatomical findings in eight powerlifters. *Journal of Sports Medicine and Physical Fitness*, *60*(4), 582-593. <https://doi.org/10.23736/S0022-4707.19.10201-0>

Brown, E. W., & Kimball, R. G. (1983). Medical history associated with adolescent powerlifting. *Pediatrics*, *72*(5), 636-644. <https://doi.org/10.1542/peds.72.5.636>

Haykowsky, M. J., Warburton, D. E. R., & Quinney, H. A. (1999). Pain and injury associated with powerlifting training in visually impaired athletes. *Journal of Visual Impairment and Blindness*, *93*(4), 236-241. <https://doi.org/10.1177/0145482x9909300406>

Jarraya, M., Blauwet, C. A., Crema, M. D., Heiss, R., Roemer, F. W., Hayashi, D., Derman, W. E., & Guermazi, A. (2021). Sports injuries at the Rio de Janeiro 2016 Summer Paralympic Games: Use of diagnostic imaging services. *European Radiology*, *31*(9), 6768-6779. <https://doi.org/10.1007/s00330-021-07802-3>

Keogh, J., Hume, P. A., & Pearson, S. (2006). Retrospective injury epidemiology of one hundred one competitive Oceania power lifters: The effects of age, body mass, competitive standard, and gender. *Journal of Strength and Conditioning Research*, *20*(3), 672-681. <https://doi.org/10.1519/R-18325.1>

Larsson, H., Strömbäck, E., Schau, M., Johansson, S., & Berglund, L. (2024). Lumbopelvic movement control in powerlifters with and without low back pain. *Physical Therapy in Sport*, *65*, 74-82. <https://doi.org/10.1016/j.ptsp.2023.11.006>

Marotta, N., de Sire, A., Bartalotta, I., Sgro, M., Zito, R., Invernizzi, M., Ammendolia, A., & Iona, T. (2024). Role of the flexion relaxation phenomenon in the analysis of low back pain risk in the powerlifter: A proof-of-principle study. *Journal of Sport Rehabilitation*, *33*(5), 333-339. <https://doi.org/10.1123/jsr.2023-0244>

Olofsson, P., Aasa, U., & Berglund, L. (2024). Development of a comprehensive clinical assessment protocol for low back and hip pain in powerlifters: A feasibility study. *Pilot and Feasibility Studies*, *10*(1), 150. <https://doi.org/10.1186/s40814-024-01579-0>

Ona Ayala, K. E., Li, X., Huang, P., Derman, W. E., Kissick, J., Webborn, N., Blauwet, C., Stomphorst, J., & Tuakli‐Wosornu, Y. A. (2019). Injury epidemiology and preparedness in powerlifting at the Rio 2016 Paralympic Games: An analysis of 1410 athlete‐days. *Translational Sports Medicine*, *2*(6), 358-369. <https://doi.org/10.1002/tsm2.107>

Pinheiro, L. S. P., Silva, A., Madaleno, F. O., Verhagen, E., de Mello, M. T., Ocarino, J. M., & Resende, R. A. (2024). Prevalence and incidence of health problems and their characteristics in Brazilian para athletes: A one-season single-center prospective pilot study. *Disability and Health Journal*, *17*(1), 101511. <https://doi.org/10.1016/j.dhjo.2023.101511>

Raske, A., & Norlin, R. (2002). Injury incidence and prevalence among elite weight and power lifters. *American Journal of Sports Medicine*, *30*(2), 248-256. <https://doi.org/10.1177/03635465020300021701>

Reichel, T., Mitnacht, M., Fenwick, A., Meffert, R., Hoos, O., & Fehske, K. (2019). Incidence and characteristics of acute and overuse injuries in elite powerlifters. *Cogent Medicine*, *6*(1), 1588192. <https://doi.org/10.1080/2331205X.2019.1588192>

Resende, R., Madaleno, F., Verhagen, E., Wezenbeek, E., de Mello, M. T., Chagas, M. H., Gonçalves, D., Silva, A., Pinheiro, L., Ocarino, J., & Witvrouw, E. (2025). Comprehensive 1-year multilevel study of sports injuries in para athletes: Impact of season timing, years of sports experience, impairment and sports type. *BMJ Open Sport and Exercise Medicine*, *11*(2), e002474. <https://doi.org/10.1136/bmjsem-2025-002474>

Severin, A. C., Kinderen, A., & Baumgart, J. K. (2025). Effect of the athletes' origin and type of impairment on participation and the likelihood of winning a medal in the Paralympic games. *American Journal of Physical Medicine and Rehabilitation*, *104*(2), 184-192. <https://doi.org/10.1097/PHM.0000000000002548>

Siewe, J., Rudat, J., Röllinghoff, M., Schlegel, U. J., Eysel, P., & Michael, J. W. P. (2011). Injuries and overuse syndromes in powerlifting. *International Journal of Sports Medicine*, *32*(9), 703-711. <https://doi.org/10.1055/s-0031-1277207>

Sjöberg, H., Aasa, U., Rosengren, M., & Berglund, L. (2020). Content validity index and reliability of a new protocol for evaluation of lifting technique in the powerlifting squat and deadlift. *Journal of Strength and Conditioning Research*, *34*(9), 2528-2536. <https://doi.org/10.1519/JSC.0000000000002791>

Strömbäck, E., Aasa, U., Gilenstam, K., & Berglund, L. (2018). Prevalence and consequences of injuries in powerlifting: A cross-sectional study. *Orthopaedic Journal of Sports Medicine*, *6*(5), 1-1. <https://doi.org/10.1177/2325967118771016>

Willick, S. E., Cushman, D. M., Blauwet, C. A., Emery, C., Webborn, N., Derman, W., Schwellnus, M., Stomphorst, J., & Van de Vliet, P. (2016). The epidemiology of injuries in powerlifting at the London 2012 Paralympic Games: An analysis of 1411 athlete-days. *Scandinavian Journal of Medicine and Science in Sports*, *26*(10), 1233-1238. <https://doi.org/10.1111/sms.12554>
