# Supplementary material for: The Applied Sport Science and Medicine of Powerlifting and Para Powerlifting: A Systematic Scoping Review with Recommendations for Future Research
Source: Sports Med. 2025 Sep 9;55(11):2849–77. doi: 10.1007/s40279-025-02305-3 (PMC12559058; doi:10.1007/s40279-025-02305-3)
Supplement: Supplementary file 6 — Supplementary file6 (DOCX 28 KB) [file 40279_2025_2305_MOESM6_ESM.docx]

**Supplementary Table S6.** Characteristics, outcomes measures, and key findings of ‘psychology’ studies (n = 8)

| **Study** | **Cohort and sample size (n); age; body mass (where specified)** | **Competitive characteristics: para status; division; weight class; age category (where specified)** | **Study aim(s)** | **Outcome measures** | **Key findings** |
| --- | --- | --- | --- | --- | --- |
| The significance of ‘situated learning’ for doping in an elite sports community: an interview study of AAS-using powerlifters (Hoff, 2021) | 10 male former competitive powerlifters | Non-para | To analyse situated learning in relation to doping in an elite sport environment, specifically in the powerlifting community | Subjective measures of doping experiences | The informants described how their doping behaviours emerged in relation to what could be interpreted as a ‘doping culture’ in their sports community |
| The impact of competitive trait anxiety on collegiate powerlifting performance (Judge et al., 2016) | 26 male and 10 female collegiate powerlifters; 19.9 ± 1.5 yrs; 81.4 ± 21.0 kg | Non-para; 47.6 kg: 1, 51.7 kg: 1, 54.9 kg: 1, 59.8 kg: 3, 67.1 kg: 2, 74.8 kg: 7, 82.1 kg: 4, 89.8 kg: 9, 99.8 kg: 5, and unlimited or super heavy weight: 3 | To determine whether a relationship exists between competitive trait anxiety measures and powerlifting performance. | Subjective measures of competitive trait anxiety (Sport Competition Anxiety Test) and personal characteristics; competitive results | Competitive trait anxiety may have negatively impacted performance, and some powerlifters may benefit from interventions aimed at decreasing anxiety before and during performance |
| Gender aspects of confounding factors in the preparation of powerlifters (Ljdokova et al., 2015) | 80 male and 80 female powerlifters; 16-49 yrs | Non-para | To analyse the first three factors that are most significant for men and women in the course of training and competitive activities | Subjective measures of confounding factors for training | The results of the study do not reveal fundamental difference in defining significant confounding factors by male and female powerlifters |
| Confounding factors in sport activities of powerlifters (Ljdokova et al., 2014) | 80 male and 80 female powerlifters; 16-49 yrs | Non-para | To determine the importance of confounding factors in training and competitive activities of athletes involved in powerlifting | Subjective measures of confounding factors for training | The confounding factors are largely interrelated; all environmental aspects of life, including lifestyle, training methodology, family relationships, how to find training partners, pre-competitive mode, and behaviour in everyday life affect competitive performance |
| Expectancy effects and strength training: Do steroids make a difference? (Maganaris et al., 2000) | 11 national level male powerlifters; 18-24 yrs; 79.4-93.4 kg | Non-para | To evaluate the degree to which performance improvements may be due to expectancies about anabolic steroids without producing any pharmacological effects | Squat, bench press, and deadlift 1RM | Notable improvements in performance associated with the belief that anabolic steroids had been administered largely dissipated when athletes were informed as to the true nature of the drug |
| Differences in motivation during the bench press movement with progressive loads using EEG analysis (Maszczyk et al., 2019) | 8 elite (22.7 ± 3.2 yrs; 81.8 ± 3.2 kg) and 8 novice powerlifters (22.4 ± 2.8 years; 79.2 ± 2.6 kg) | Non-para | To identify the patterns of motivation activity of the prime movers by alpha frequency band analysis (named as alpha motivation values) for each 35-100% 1RM during the flat bench press, and to identify differences in brain activity between novice and expert weightlifters with different workloads | Electroencephalographic recordings of frequency power over left and right frontal regions during bench press at 35-95% 1RM | Novices applied significantly more motivation at initial loads than elite powerlifters; the most effective motivation for novices was at 35% 1RM, 50% 1RM and 90% 1RM with significant differences between groups; for 95% 1RM and 100% 1RM a significant decrease of alpha values was recorded for novice powerlifters (decreased motivation); elite powerlifters varied their motivation more to optimise performance in the final attempts with max loads |
| Attentional style and powerlifting performance (McGowan et al., 1990) | 78 male and 31 female National Collegiate Powerlifting Championships powerlifters | Non-para | To examine whether the subscales included in the abbreviated Test of Attentional and Interpersonal Style discriminated between successful and less successful performers when attentional demands and arousal levels were known | Subjective measures of attentional style | Less successful lifters scored higher on the Narrowing and Broad Internal subscales than the successful lifters |
| The impact of EEG biofeedback training on the athletes’ motivation and bench press performance (Prończuk et al., 2024) | 9 advanced powerlifters (82 ± 2.512 kg) and 9 intermediate (81 ± 1.821 kg) powerlifters | Non-para | To determine the impact of electroencephalography-biofeedback training on the motivation and efficiency of powerlifters during bench press exercises in relation to the external load and training level | Electroencephalography measurements; bench press performance | The repeated measures ANOVA showed intra-group differences due to external loading for the Frontal Alpha Asymmetry obtained in the electroencephalography both before and after biofeedback training; there was significant differences between 65%1RM and 35%1RM in the advanced group, and between 35%1RM, 50%1RM, 65%1RM, and 80%1RM in the intermediate group |

**References**

Hoff, D. (2021). The significance of ‘situated learning’ for doping in an elite sports community: An interview study of AAS-using powerlifters. *Sport in Society*, *25*(1), 197-216. <https://doi.org/10.1080/17430437.2020.1779222>

Judge, L. W., Urbina, L. J., Hoover, D. L., Craig, B. W., Judge, L. M., Leitzelar, B. M., Pearson, D. R., Holtzclaw, K. A., & Bellar, D. M. (2016). The impact of competitive trait anxiety on collegiate powerlifting performance. *Journal of Strength and Conditioning Research*, *30*(9), 2399-2405. <https://doi.org/10.1519/JSC.0000000000001363>

Ljdokova, G. M., Ismailova, N. I., Panfilov, A. N., & Farhatovich, K. A. (2015). Gender aspects of confounding factors in the preparation of powerlifters. *Biosciences Biotechnology Research Asia*, *12*(1), 393-399. <https://doi.org/10.13005/bbra/1678>

Ljdokova, G. M., Razzhivin, O. A., & Volkova, K. R. (2014). Confounding factors in sport activities of powerlifters. *Life Science Journal*, *11*(8), 410-413.

Maganaris, C. N., Collins, D., & Sharp, M. (2000). Expectancy effects and strength training: Do steroids make a difference? *Sport Psychologist*, *14*(3), 272-278. <https://doi.org/10.1123/tsp.14.3.272>

Maszczyk, A., Dobrakowski, P., Żak, M., Gozdowski, P., Krawczyk, M., Małecki, A., Stastny, P., & Zajac, T. (2019). Differences in motivation during the bench press movement with progressive loads using EEG analysis. *Biology of Sport*, *36*(4), 351-356. <https://doi.org/10.5114/biolsport.2019.88757>

McGowan, R. W., Talton, B. J., & Tobacyk, J. J. (1990). Attentional style and powerlifting performance. *Perceptual and Motor Skills*, *70*(3 II), 1253-1257. <https://doi.org/10.2466/pms.1990.70.3c.1253>

Prończuk, M., Chamera, T., Markowski, J., Pilch, J., Smólka, W., Zajac, A., & Maszczyk, A. (2024). The impact of EEG biofeedback training on the athletes' motivation and bench press performance. *Biology of Sport*, *41*(3), 97-104. <https://doi.org/10.5114/biolsport.2024.127065>
