# Supplementary material for: The Applied Sport Science and Medicine of Powerlifting and Para Powerlifting: A Systematic Scoping Review with Recommendations for Future Research
Source: Sports Med. 2025 Sep 9;55(11):2849–77. doi: 10.1007/s40279-025-02305-3 (PMC12559058; doi:10.1007/s40279-025-02305-3)
Supplement: Supplementary file 7 — Supplementary file7 (DOCX 40 KB) [file 40279_2025_2305_MOESM7_ESM.docx]

**Supplementary Table S7.** Characteristics, outcomes measures, and key findings of ‘nutrition and supplementation’ studies (n = 25)

| **Study** | **Cohort and sample size (n); age; body mass (where specified)** | **Competitive characteristics: para status; division; weight class; age category (where specified)** | **Study aim(s)** | **Outcome measures** | **Key findings** |
| --- | --- | --- | --- | --- | --- |
| Evaluation of ibuprofen use on the immune system indicators and force in disabled Paralympic powerlifters of different sport levels (Aidar et al., 2022) | 10 national level (32.50 ± 3.00 yrs; 84.00 ± 17.63 kg) and 10 regional level (30.75 ± 5.32 yrs; 74.50 ± 33.88 kg) male Paralympic powerlifters | Para | To analyse the use of ibuprofen on strength indicators and immunological blood biomarkers in Paralympic powerlifting at national and regional levels in the post-workout recovery period | Isometric peak torque; rate of torque development; fatigue index; immunological blood biomarkers | The use of ibuprofen had a positive effect on isometric strength, fatigue, and rate of torque development; there were no differences in blood indicators related to the immune system, except for basophils, indicating that this indicator was higher in regional athletes who used the placebo; the level of training tends to influence the indicators related to the immune system, being, together with the use of ibuprofen, an important ally in post-workout recovery |
| Effects of ibuprofen use on lymphocyte count and oxidative stress in elite Paralympic powerlifting (Aidar et al., 2021) | 10 male Paralympic powerlifters; 27.13 ± 5.57 yrs; 79.25 ± 25.51 kg | Para | To analyse the effects of the use of ibuprofen on performance parameters, cell count, and oxidative stress in national level Paralympic powerlifters in the period of resisted post-training recovery | Isometric peak torque; rate of torque development; fatigue index; blood cell and leukocyte count; oxidative stress markers | The ibuprofen had a positive effect on strength indicators, with decreased fatigue and increased lymphocyte count; there were no differences in oxidative stress |
| A comparison of rapid weight loss practices within international, national and regional powerlifters (Campbell et al., 2023) | 36 male and 33 female British powerlifters (22 international level, 32 national level, 15 regional level); 29.7 ± 7.4 yrs | Non-para; 43 classic and 26 equipped, tested; males: 59kg=1, 66kg=3, 74kg=7, 83kg=2, 93kg=12, 105kg=8, 120kg=3, and females: 47kg=1, 52kg=5, 57kg=6, 63kg=9, 72kg=8, 84kg= 4 | To describe the rapid weight loss practices of International Powerlifting Federation powerlifters from Great Britain and to compare the rapid weight loss practices of those competing at international, national, and regional level | Subjective measures of rapid weight loss practices and experiences | The prevalence of rapid weight loss is high among competitive powerlifters, with many competitors attributing negative perceptions during the weight-cutting process; rapid weight loss within regional lifters was beyond where performance decrements are commonly seen |
| A study of steroid use among athletes: knowledge, attitude, and use (Chng & Moore, 1990) | 116 male and 106 female athletes (32 powerlifters); median 24 yrs | Non-para | To provide preliminary data on the relationship among knowledge, attitude, and prevalence of steroid use for a sample of athletes and non-athletes | Subjective measures of steroid use, attitudes, and knowledge | Powerlifters and bodybuilders were more likely to use steroids than non-lifters; steroid users were more knowledgeable and had more positive attitudes towards steroids; greater knowledge was correlated with more positive attitudes |
| Qualitative description of the prevalence and use of anabolic androgenic steroids by United States powerlifters (Curry & Wagman, 1999) | 15 elite American powerlifters; 23-45 yrs | Non-para | To elicit yes/no responses and open reflective commentary from United States powerlifters on issues related to the use of anabolic androgenic steroids and the effectiveness of doping controls | Subjective measures of anabolic androgenic steroid use and perceptions | 10 United States Powerlifting Team members admitted to using anabolic androgenic steroids, and 5 athletes admitted to beating the International Olympic Committee’s doping control procedures |
| Effects of ibuprofen intake in muscle damage, body temperature and muscle power in Paralympic powerlifting athletes (Fraga et al., 2020) | 8 Paralympic powerlifters; 27.0 ± 5.3 yrs; 79.9 ± 25.3 kg | Para | To analyse the effect of ibuprofen on post-workout recovery in Paralympic powerlifting athletes | Upper limb maximal isometric force and rate of force development; thermography; serum biochemical analyses of creatine kinase, lactate dehydrogenase, aspartate aminotransferase, and alanine aminotransferase | The results indicate some positive effects of ibuprofen use but do not enable a clear statement regarding its positive effects on muscle function and muscle damage; ibuprofen seems to have caused a delay in the anti-inflammatory response following exercise |
| Effect of oral creatine supplementation on near-maximal strength and repeated sets of high-intensity bench press exercise (Kelly & Jenkins, 1998) | 18 male powerlifters; 26.8 ± 6.1 yrs; 84.83 ± 15.79 kg | Non-para | To examine the effect of a relatively high initial dose of creatine supplementation followed by a lower dose on near-maximal bench press, and changes in body composition in competitive powerlifters | Near-maximal muscular strength; body mass; body fat percentage | It appears that 26 days of creatine supplementation significantly improves muscular strength and repeated near-maximal bench press performance, and induces changes in body composition |
| The general nutrition practices of competitive powerlifters vary by competitive calibre and sex, weight, and age class (King et al., 2023) | 240 male and 65 female competitive powerlifters | Non-para; 19 sub-junior, 118 junior, 149 open, 19 masters | To survey the nutrition practices of competitive powerlifters around the competitive cycle, and individual training sessions | Subjective measures of nutritional practices | Flexible dieting is commonly followed by powerlifters to support performance and body composition goals; females seemed to report more often restricting energy and dieting for body composition reasons than males; powerlifters tailor their energy intake on harder training days to the higher training demands but refrain from reducing energy intake on rest/easier training days |
| Fueling for and recovering from resistance training: The periworkout nutrition practices of competitive powerlifters (King et al., 2024) | 240 male and 65 female competitive powerlifters | Non-para; 19 sub-junior, 118 junior, 149 open, 19 masters | To survey actively competing powerlifters on their current acute nutrition practices around training | Subjective measures of nutritional practices | The periworkout nutrition practices used by competitive powerlifters followed current sport nutrition guidelines, by using carbohydrate sources to fuel for training and ensuring the provision of protein postexercise |
| An assessment of the consumption of dietary supplements by players of selected sports (Kozirok et al., 2013) | 100 male powerlifters and 55 male and 61 female volleyball players; 16-30 yrs | Non-para | To assess the use of dietary supplements by players of selected sports with consideration given to sex, age, education, level of physical activity and training experience | Subjective measures of nutritional and supplementary practices | Powerlifters were the most frequent users of creatine, protein/carbohydrate preparations, BCAAs, and caffeine, while volleyball players most frequently used vitamin and mineral preparations, creatine, and L-carnitine |
| Dehydration and other strategies of rapid weight loss in young powerlifters (Kruszewski et al., 2022) | 98 youth class 1 and 2 powerlifters; 14-18 years; Group 1 – up to 75 kg (n=50), Group 2 – above 75 kg (n=48) | Non-para; up to 75 kg (n = 50) and above 75 kg (n = 48); youth class 1 and 2 | To assess practices and knowledge about rapid weight loss methods and their potential detrimental effects on health in young powerlifters | Subjective measures of rapid weight loss methods | Dehydration was the most common rapid weight loss method reported among young powerlifters, with high weight class athletes using it more than low weight class athletes; diet and diuretics were commonly recognised rapid weight loss methods; aerobic exercise and fluid restriction were rarely known as rapid weight loss methods; the diuretic effects of vitamin C and potassium were less known; syncope and muscle cramps were the most commonly known negative effects of dehydration |
| Prevalence, magnitude, and methods of weight cutting used by world class powerlifters (Kwan & Helms, 2022) | 42 male and 22 female elite powerlifters; 26.2 ± 6.5 yrs; 72.2 ± 16.1 kg | Non-para; raw; all weight classes except 120 kg for males and 84 kg for females; all age categories | To examine the prevalence of weight cutting practices at the elite level and to assess whether there were any associations between weight cutting practices, competitive success, and mood state and perceived stress | Subjective measures of rapid weight loss strategies and perceived psychological states | There was a high prevalence of weight cutting, with 83% of athletes using a combination of rapid weight loss and gradual dieting; athletes who performed a weight cut were more likely to win medals; medallists had a higher average rapid weight loss score than non-medallists; the average rapid weight loss was 2.9% and the average rapid weight gain was 2.6%; the most common rapid weight loss methods were water loading and cutting, and gradual dieting; increased anger, fatigue, and anxiety were reported from weight cutting |
| Trends in dietary supplement use among athletes selected for doping controls (Lauritzen & Gjelstad, 2023) | Powerlifters (sample size NR) | NR | To examine the use of dietary supplements among athletes who have participated in doping tests by using information derived from doping control forms collected by Anti-Doping Norway from 2015-2019 | Doping control form assessing the year, gender, age group, athlete level, sport discipline, type of test, nationality, and reported use of dietary supplements during the seven days preceding the doping control | The highest prevalence of dietary supplement use was in powerlifting (79%); the highest average supplement use per doping control form was in powerlifting; common supplements used in powerlifting were sports products (48%), ergogenic substances (48%), medical supplements (43.1%), and mixed products (27%) |
| Nutrition knowledge and dietary adequacy in powerlifters (Leonhardt et al., 2024) | 13 male and 10 female regional, provincial, national, or international level powerlifters; 31 ± 11 yrs; 91.0 ± 30.2 kg | Non-para | To assess the effect of nutrition knowledge, sex, and time (off-season versus pre-competition, when athletes often cut weight) on dietary adequacy in powerlifters | Subjective measures of nutritional practices | Nutrition knowledge, sex, and time affect dietary intake of powerlifters; a higher nutrition knowledge predicts a higher intake of many foods and nutrients important for maintenance of health and performance |
| Micronutrient supplement intakes among collegiate and masters athletes: A cross-sectional study (Nichols et al., 2023) | 93 male and 105 female collegiate (22.07 ± 2.06 yrs; 71.52 ± 13.38 kg) or masters (40.19 ± 11.05 yrs; 72.86 ± 13.38 kg) athletes (4 powerlifters) | NR | To compare the differences in vitamin and mineral supplementation between the sexes and among various sport classifications in collegiate athletes and masters athletes | Subjective measures of micronutrient supplementation consumption | There were significant differences among male and female collegiate and masters athletes, and between collegiate and masters athletes; masters powerlifters had significantly greater zinc intakes compared to some other athletes |
| Self-reported prevalence, magnitude, and methods of rapid weight loss in male and female competitive powerlifters (Nolan et al., 2022) | 141 male and 92 female powerlifters; 28.5 ± 8.5 yrs; 80.0 ± 17.2 kg | Non-para; raw tested; all weight classes | To provide data on the self-reported prevalence, magnitude, and methods of rapid weight loss used by competitive powerlifters | Subjective measures of rapid weight loss practices | The magnitude of rapid weight loss is typically ~3% of body mass (largely similar across sex, weight category, and athlete calibre); gradual dieting along with body water manipulation through water-loading and fluid-restriction are the most commonly used methods of rapid weight loss by powerlifters; coaches and online resources were cited as the major influences on the rapid weight loss practices of these athletes |
| Ammonia inhalant & stimulant use among powerlifters: Results from an international survey (Pritchard et al., 2014) | 256 male powerlifters; 101.7 ± 21.2 kg | NR | To determine whether the anecdotal use of stimulants by powerlifters is prevalent within the sport, as well as whether the users of these stimulants believe them to be effective and safe | Subjective measures of stimulant use and powerlifting experience | Nearly half of the surveyed powerlifters responded affirmatively to having used ammonia inhalants in competition; the majority of those who used ammonia inhalants mainly do so on the deadlift compared to the squat or bench press |
| The effect of creatine monohydrate loading on maximal intermittent exercise and sport-specific strength in well trained power-lifters (Rossouw et al., 2000) | 14 male and 2 female competitive powerlifters | Non-para | To investigate the effect of creatine supplementation on the performance of well-trained power athletes performing intermittent bouts of high intensity exercise in a laboratory environment as well as performing a sport-specific strength feat under field conditions | Anthropometric measurements; maximal unilateral knee extension peak torque, explosive power, total work, average power, work in first and last 5 repetitions; deadlift 1RM | Values for peak torque, average power, total work and work output during the first five sample repetitions in the creatine group increased significantly and in a relatively constant fashion in all subjects after five supplementation days; there was also a significant increase in the deadlift lifting volume after six days of creatine supplementation |
| Can creatine supplementation interfere with muscle strength and fatigue in Brazilian national level Paralympic powerlifting (Soares Freitas Sampaio et al., 2020) | 8 national level Paralympic powerlifters | Para | To analyse the effects of creatine supplementation on indicators of torque, force, time, and fatigue index in Paralympic powerlifting athletes | Muscle strength; fatigue index; peak torque; force; rate of force development; time to maximum isometric force | Creatine supplementation did not show effects on the variables of muscle force, peak torque, rate of force development, and time to maximum isometric force; however, the fatigue index was higher for placebo after 7 days |
| Creatine monohydrate use among elite Australian power lifters (Stanton & Abt, 2000) | 50 national level Powerlifting Australia powerlifters; 33.5 ± 7.9 yrs | Non-para | To determine the prevalence, perceived advantages and disadvantages, side effects, and methods of creatine monohydrate use among elite Australian powerlifters | Subjective measures of supplementation practices | 96% of respondents were aware of creatine; 74% identified themselves as current or former users; powerlifters agreed that creatine improved the quality of their training and competitive performance; an increase in body mass was the most common side effect; 70% of users reported a cyclic method of intake, 14% reported a regular intake, and 16% reported an intermittent intake |
| The prevalence of doping in Flanders in comparison to the prevalence of doping in international sports (Van Eenoo & Delbeke, 2003) | 258 drug testing samples for powerlifting from 1993-2000 and drug testing samples from other sports | NR | To investigate the prevalence of prohibited substances in sports to illustrate trends and raise awareness on the needs to eventually implement new doping control methods | Drug testing results | The use of doping among male athletes is significantly higher than for female athletes; bodybuilding and powerlifting had the highest incidence of positive cases in Flanders |
| Distribution of caffeine levels in urine in different sports in relation to doping control before and after the removal of caffeine from the WADA doping list (Van Thuyne & Delbeke, 2006) | 4633 urine samples for powerlifting and other sports from 1993-2002 and 2004 | NR | To monitor caffeine concentrations in 4633 urine samples tested for doping control after the removal of caffeine from the doping list in 2004 | Caffeine testing results | Comparison of the most frequently tested sports in 2004 demonstrated that caffeine concentrations in samples originating from powerlifters are significantly higher in comparison to urine samples taken in other sports |
| An investigation into anabolic androgenic steroid use by elite U.S. powerlifters (Wagman et al., 1995) | 15 international level powerlifters | Non-para; tested | To investigate previous and current anabolic androgenic steroid use by elite powerlifters at national/international competitions where International Olympic Committee standards for doping controls were implemented | Subjective measures of anabolic androgenic steroid use and perceptions | Two-thirds of the respondents had used anabolic androgenic steroids, and 90% of the users indicated that cycling off anabolic androgenic steroids between 1 and 3 months prior to competition was the most effective method for passing doping controls |
| Quantifying frequency of use of methods of body mass loss in competing UK powerlifters (Wood et al., 2022) | 18 male and 19 female competitive powerlifters | Non-para; all weight classes; junior and open categories | To assess the frequency with which certain rapid weight loss methods are adopted by male and female powerlifting athletes in the United Kingdom during competition preparation | Subjective measures of rapid weight loss practices | Commonly reported methods of weight loss were gradual dieting (49%), fluid restriction (46%), and water loading (51%); differences between powerlifting category and adopting rapid weight loss were observed; powerlifting category was a predictor of undertaking rapid weight loss while biological sex was a predictor of timeframe of undertaking rapid weight loss |
| Self-reported use of anabolic-androgenic steroids by elite power lifters (Yesalis III et al., 1988) | 45 national level powerlifters | Non-para; tested | To measure the rate of previous steroid use and to characterise the self-reported user-group based on attitudes, patterns of use, and associated health effects | Subjective measures of anabolic androgenic steroid use, attitudes, and associated health effects | Of the 45 who responded to the survey, 15 admitted having used steroids; in a follow-up telephone interview of 20 of the competitors, 11 reported previous steroid use; the reason given most often for using steroids was improved athletic performance; the most common side effects reported were heightened libido, acne, and increased body hair; the small number of admitted users suggests that underreporting took place; this level of use probably represents the lower bound of steroid use among powerlifters |

**References**

Aidar, F. J., Fraga, G. S., Getirana-Mota, M., Marçal, A. C., Santos, J. L., de Souza, R. F., Ferreira, A. R. P., Neves, E. B., Zanona, A. d. F., Bulhões-Correia, A., de Almeida-Neto, P. F., Fernandes, T. L. B., Garrido, N. D., Cirilo-Sousa, M. d. S., Merino-Fernández, M., Díaz-de-Durana, A. L., Murawska-Ciałowicz, E., Cabral, B. G. d. A. T., & Clemente, F. M. (2021). Effects of ibuprofen use on lymphocyte count and oxidative stress in elite Paralympic powerlifting. *Biology*, *10*(10), 986. <https://doi.org/10.3390/biology10100986>

Aidar, F. J., Fraga, G. S., Getirana-Mota, M., Marçal, A. C., Santos, J. L., de Souza, R. F., Vieira-Souza, L. M., Ferreira, A. R. P., de Matos, D. G., de Almeida-Neto, P. F., Garrido, N. D., Díaz-de-Durana, A. L., Knechtle, B., de Araújo Tinoco Cabral, B. G., Murawska-Ciałowicz, E., Nobari, H., Silva, A. F., Clemente, F. M., & Badicu, G. (2022). Evaluation of ibuprofen use on the immune system indicators and force in disabled Paralympic powerlifters of different sport levels. *Healthcare*, *10*(7), 1331. <https://doi.org/10.3390/healthcare10071331>

Campbell, P., Martin, D., Bargh, M. J., & Gee, T. I. (2023). A comparison of rapid weight loss practices within international, national and regional powerlifters. *Nutrition and Health*, 2601060231201892. <https://doi.org/10.1177/02601060231201892>

Chng, C. L., & Moore, A. (1990). A study of steroid use among athletes: Knowledge, attitude and use. *Health Education*, *21*(6), 12-17. <https://doi.org/10.1080/00970050.1990.10614585>

Curry, L. A., & Wagman, D. F. (1999). Qualitative description of the prevalence and use of anabolic androgenic steroids by United States powerlifters. *Perceptual and Motor Skills*, *88*(1), 224-233. <https://doi.org/10.2466/pms.1999.88.1.224>

Fraga, G. S., Aidar, F. J., Matos, D. G., Marçal, A. C., Santos, J. L., Souza, R. F., Carneiro, A. L., Vasconcelos, A. B., Da Silva-Grigoletto, M. E., van den Tillaar, R., Cabral, B. T., & Reis, V. M. (2020). Effects of ibuprofen intake in muscle damage, body temperature and muscle power in Paralympic powerlifting athletes. *International Journal of Environmental Research and Public Health*, *17*(14). <https://doi.org/10.3390/ijerph17145157>

Kelly, V. G., & Jenkins, D. G. (1998). Effect of oral creatine supplementation on near-maximal strength and repeated sets of high-intensity bench press exercise. *Journal of Strength and Conditioning Research*, *12*(2), 109-115.

King, A., Kwan, K., Jukic, I., Zinn, C., & Helms, E. (2023). The general nutrition practices of competitive powerlifters vary by competitive calibre and sex, weight, and age class. *European Journal of Nutrition*, *62*(8), 3297-3310. <https://doi.org/10.1007/s00394-023-03233-6>

King, A., Kwan, K., Jukic, I., Zinn, C., & Helms, E. (2024). Fueling for and recovering from resistance training: The periworkout nutrition practices of competitive powerlifters. *Nutrition*, *122*, 112389. <https://doi.org/10.1016/j.nut.2024.112389>

Kozirok, W., Babicz-Zielińska, E., & Krzebietke, B. (2013). An assessment of the consumption of dietary supplements by players of selected sports. *Polish Journal of Sport and Tourism*, *20*(2), 123-128. <https://doi.org/10.2478/pjst-2013-0012>

Kruszewski, M., Pągowski, Ł., Kruszewski, A., Tabęcki, R., & Merchelski, M. (2022). Dehydration and other strategies of rapid weight loss in young powerlifters. *Journal of Kinesiology and Exercise Sciences*, *32*(98), 11-20. <https://doi.org/10.5604/01.3001.0015.8565>

Kwan, K., & Helms, E. (2022). Prevalence, magnitude, and methods of weight cutting used by world class powerlifters. *Journal of Strength and Conditioning Research*, *36*(4), 998-1002. <https://doi.org/10.1519/JSC.0000000000004199>

Lauritzen, F., & Gjelstad, A. (2023). Trends in dietary supplement use among athletes selected for doping controls. *Frontiers in Nutrition*, *10*, 1143187. <https://doi.org/10.3389/fnut.2023.1143187>

Leonhardt, T. P. M., Chilibeck, P. D., Ko, J., & Zello, G. A. (2024). Nutrition knowledge and dietary adequacy in powerlifters. *Journal of Strength and Conditioning Research*, *38*(11), 1924-1932. <https://doi.org/10.1519/JSC.0000000000004887>

Nichols, Q. Z., Ramadoss, R., Stanzione, J. R., & Volpe, S. L. (2023). Micronutrient supplement intakes among collegiate and masters athletes: A cross-sectional study. *Frontiers in Sports and Active Living*, *5*, 854442. <https://doi.org/10.3389/fspor.2023.854442>

Nolan, D., Lynch, A. E., & Egan, B. (2022). Self-reported prevalence, magnitude, and methods of rapid weight loss in male and female competitive powerlifters. *Journal of Strength and Conditioning Research*, *36*(2), 405-410. <https://doi.org/10.1519/JSC.0000000000003488>

Pritchard, H. J., Stannard, S. R., & Barnes, M. J. (2014). Ammonia inhalant & stimulant use among powerlifters: Results from an international survey. *Journal of Australian Strength and Conditioning*, *22*(5), 52-54.

Rossouw, F., Krüger, P. E., & Rossouw, J. (2000). The effect of creatine monohydrate loading on maximal intermittent exercise and sport-specific strength in well trained power-lifters. *Nutrition Research*, *20*(4), 505-514. <https://doi.org/10.1016/S0271-5317(00)00142-1>

Soares Freitas Sampaio, C. R., Aidar, F. J., Ferreira, A. R. P., Santos, J. L. d., Marçal, A. C., Matos, D. G. d., Souza, R. F. d., Moreira, O. C., Guerra, I., Fernandes Filho, J., Marcucci-Barbosa, L. S., Nunes-Silva, A., Almeida-Neto, P. F. d., Cabral, B. G. A. T., & Reis, V. M. (2020). Can creatine supplementation interfere with muscle strength and fatigue in Brazilian national level Paralympic powerlifting? *Nutrients*, *12*(9), 2492. <https://doi.org/10.3390/nu12092492>

Stanton, R., & Abt, G. A. (2000). Creatine monohydrate use among elite Australian power lifters. *Journal of Strength and Conditioning Research*, *14*(3), 322-327.

Van Eenoo, P., & Delbeke, F. T. (2003). The prevalence of doping in Flanders in comparison to the prevalence of doping in international sports. *International Journal of Sports Medicine*, *24*(8), 565-570. <https://doi.org/10.1055/s-2003-43269>

Van Thuyne, W., & Delbeke, F. T. (2006). Distribution of caffeine levels in urine in different sports in relation to doping control before and after the removal of caffeine from the WADA doping list. *International Journal of Sports Medicine*, *27*(9), 745-750. <https://doi.org/10.1055/s-2005-872921>

Wagman, D. F., Curry, L. A., & Cook, D. L. (1995). An investigation into anabolic androgenic steroid use by elite U.S. powerlifters. *Journal of Strength and Conditioning Research*, *9*(3), 149-154.

Wood, T. J., Wilson, L. J., & Curtis, C. (2022). Quantifying frequency of use of methods of body mass loss in competing UK powerlifters. *Performance Enhancement and Health*, *10*(2). <https://doi.org/10.1016/j.peh.2022.100221>

Yesalis III, C. E., Herrick, R. T., Buckley, W. E., Friedl, K. E., Brannon, D., & Wright, J. E. (1988). Self-reported use of anabolic-androgenic steroids by elite power lifters. *The Physician and Sportsmedicine*, *16*(12), 91-100. <https://doi.org/10.1080/00913847.1988.11709666>
